# Bisspirooxindole synthesis: a comprehensive review of starting material-based strategies (2006–2026)†

**DOI:** 10.1039/d6ra07632g

**Published:** 2026-11-11

**Authors:** Fereshteh Ahmadi, Zahra Abdi, Mohammad Javadi

**Affiliations:** a Department of Chemistry, K. N. Toosi University of Technology Tehran Iran f.ahmadi@kntu.ac.ir

## Abstract

Bisspirooxindole frameworks constitute an important class of heterocyclic compounds because of their unique three-dimensional architectures, the presence of two spiro carbon centers, and widespread presence in biologically active natural products, pharmaceuticals, and functional materials. Owing to their structural complexity and significant pharmacological potential, the development of efficient and selective synthetic methodologies for the construction of bisspirooxindole scaffolds has attracted continuous attention in modern organic synthesis. Despite the remarkable advances achieved over the past two decades, a comprehensive review that systematically categorizes the synthetic strategies toward bisspirooxindole and highlights recent advances has not yet been presented. This review, provides the first extensive and strategy-oriented overview of synthetic methodologies for the synthesis of bisspirooxindole scaffolds reported between 2006 and 2026. The discussed approaches are systematically categorized according to the nature and structural characteristics of the starting materials, including azomethine ylide-based strategies, transition-metal-catalysted transformations, Isatin-derived Morita–Baylis–Hillman (MBH) carbonate annulations, various 3-functionalized oxindole precursors, and aniline-derived starting materials. Particular attention is devoted to reaction mechanisms, stereoselective and enantioselective synthesis, substrate scope, regioselectivity, functional-group tolerance, and synthetic efficiency. The advantages and limitations of the various methodologies are critically evaluated in terms of reaction mechanisms, substrate scope, stereoselectivity, regioselectivity, functional-group tolerance, synthetic efficiency, and their potential for environmentally benign, scalable, and highly selective synthesis. The concluding sections focus on recent advances in modern bisspirooxindole synthesis, with particular emphasis on innovative reaction designs and newly developed synthetic methodologies. In addition, synthetic approaches to dispirooxindole frameworks featuring two spirooxindole units linked through a molecular spacer are comprehensively summarized and critically discussed.

## Abstract 2

This review surveys synthetic strategies for bisspirooxindole scaffolds, highlighting reaction mechanisms, stereoselectivity, substrate scope, and recent advances.

## Graphical abstract

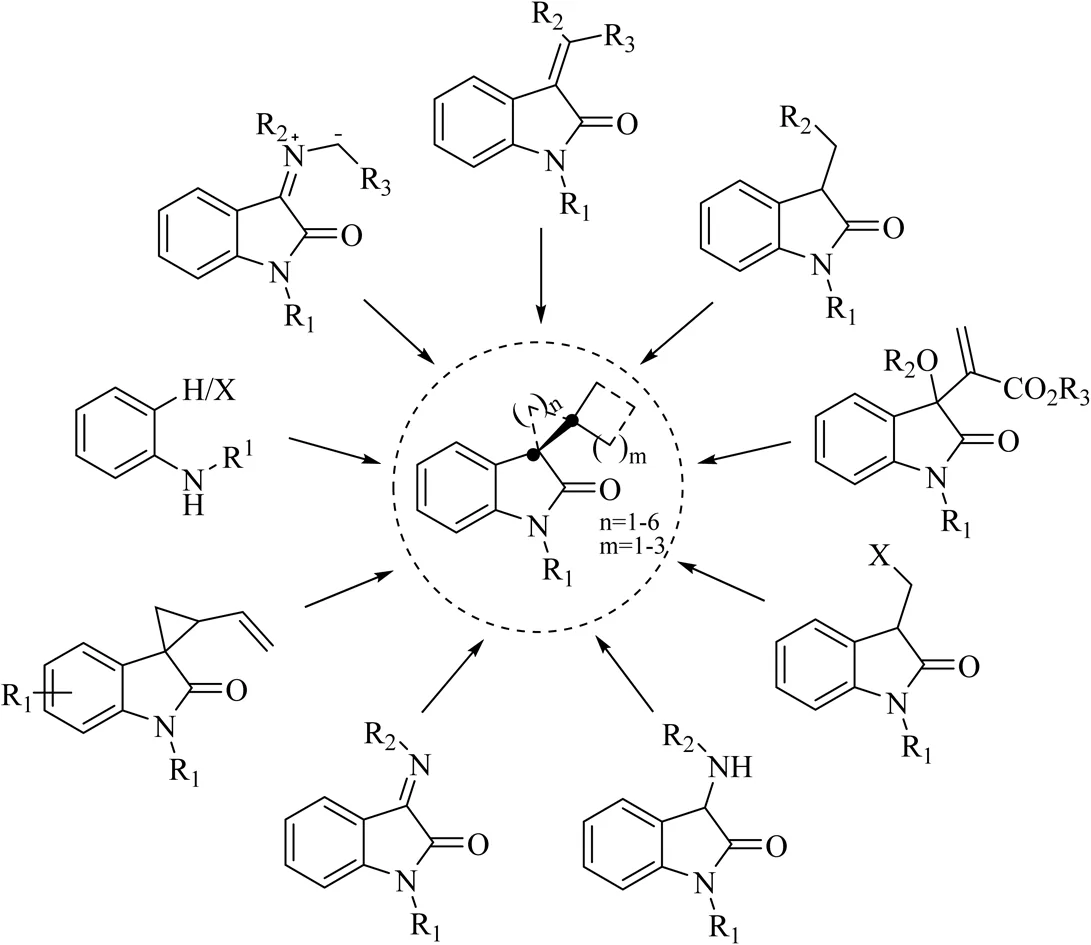

## Introduction

Isatin, also known as indoline-2,3-dione or 1H-indole-2,3-dione, is a naturally occurring compound found in plants of the *genus Isatis* and in *Couroupita guianensis Aubl*.^1,2^ It was first discovered in the 19th as an oxidation product of indigo, and its current structure was proposed by kekule.^3^ The remarkable dual reactivity of isatin, which allows it to participate in reactions as both an electrophilic and nucleophilic partner, has made it a valuable and versatile motif in organic synthesis. Isatin has been widely employed as an important substrate for the synthesis of biologically significant heterocyclic frameworks, including indoles, quinolines, pyrrolidines, 2-oxindoles, and β-lactams.^4^ The chemistry of isatin was first investigated by Sumpter^5^ and later updated by Poppand^6^ and Silva *et al.*^1^ Recent studies have demonstrated that isatin and its derivatives exhibit a broad range of biological activities, including antiviral,^7^ anti-HIV,^8^ anticonvulsant^9^ and antitumor^10^ properties, which have stimulated growing interest in their application in numerous synthetic transformations.

One of the most fascinating applications of isatin in the synthesis of organic compounds is certainly the high reactivity, particularly at the prochiral C-3 carbonyl group, enables nucleophilic additions and spiroannulation reactions, leading to the formation of 2-oxindole derivatives.^4^ The oxindole scaffold represents an important class of aromatic bicyclic heterocycles consisting of a benzene ring fused to a pyrrole moiety with a carbonyl group at its C-2 position.^11^ Due to its diverse biological and pharmacological activities, particularly α-glucosidase inhibition 1,^12^ rheumatoid arthritis 2,^13^ intraocular pressure reducing abilities 3,^14^ tyrosinase inhibition 4,^15^ and antiviral 5 (ref. 16) properties, oxindole has gained considerable prominence among nitrogen- and oxygen-containing heterocyclic compounds (Fig. 1).

**Fig. 1:**
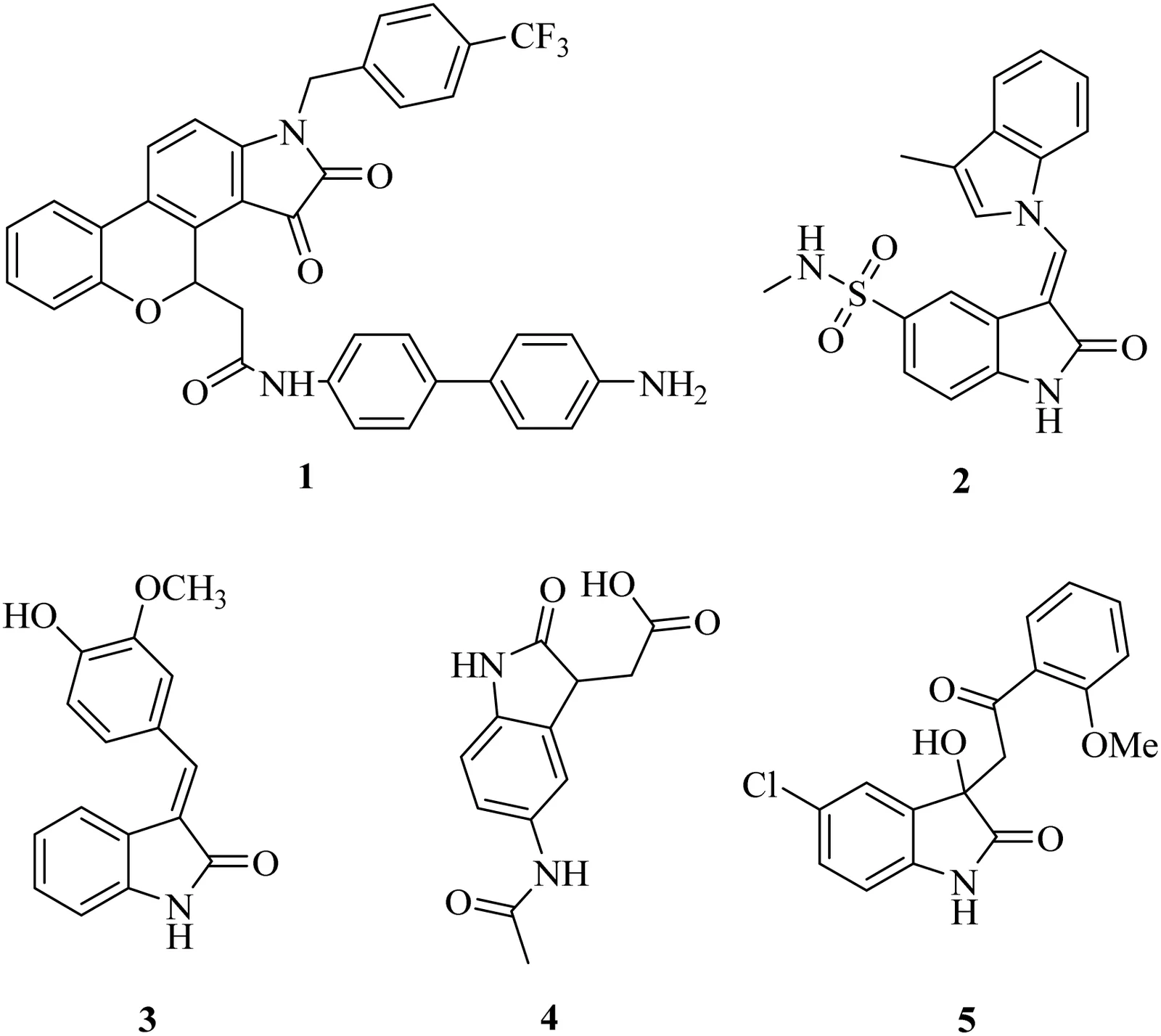
Chemical structures of several biologically active oxindole-based structure.

Oxindoles are heteroaromatic organic compounds found in mammalian tissues and fluids, as well as in various plants.^17,18^ The first naturally occurring oxindole derivative was identified as an alkaloid from *Uncaria tomentosa* (commonly known as the cat's claw plant), native to the tropical rainforests of the Amazon and other regions of Central and South America. In addition to natural methods for obtaining the oxindole nucleus, various synthetic approaches have also been developed to access this valuable skeleton.^11^ The earliest synthetic methods utilized isatin as a substrate, which were introduced by Bayer and Knop,^19^ as early as 1866. They reduced isatin 6 with sodium amalgam, followed by acidic stannum (II) treatment to produce the first synthetic oxindole 8 (Scheme 1, Path A).

**Scheme 1:**
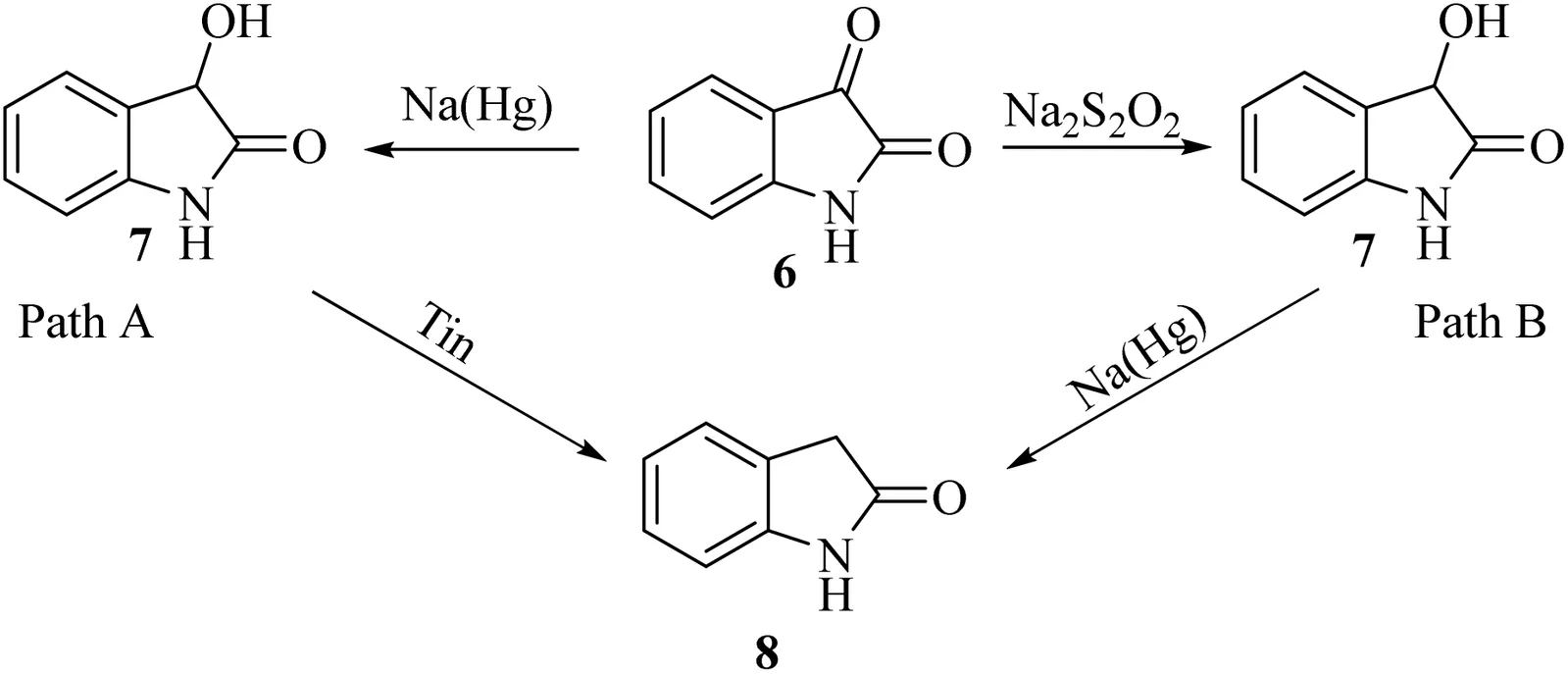
Pioneering reports on the synthesis of oxindole from isatin.

At the same time, Marschalk achieved oxindole synthesis *via* sodium hydrosulfite reduction of isatin. In 1890 Curtius and Thun further refined the process using hydrazine as a reducing agent (Scheme 1, Path B). These early methodologies established the foundation for the widespread exploration of oxindole derivatives in organic synthesis and medicinal chemistry.^20^ Oxindole-based frameworks are found in numerous commercially relevant drugs, such as ziprasidone 9 (ref. 21) and ropinirole 10, (ref. 22) as well as naturally occurring compounds, such as neolaugerine 11 (ref. 23) and (E)-Isatis,^24^ highlighting their widespread occurrence and biological significance (Fig. 2).

**Fig. 2:**
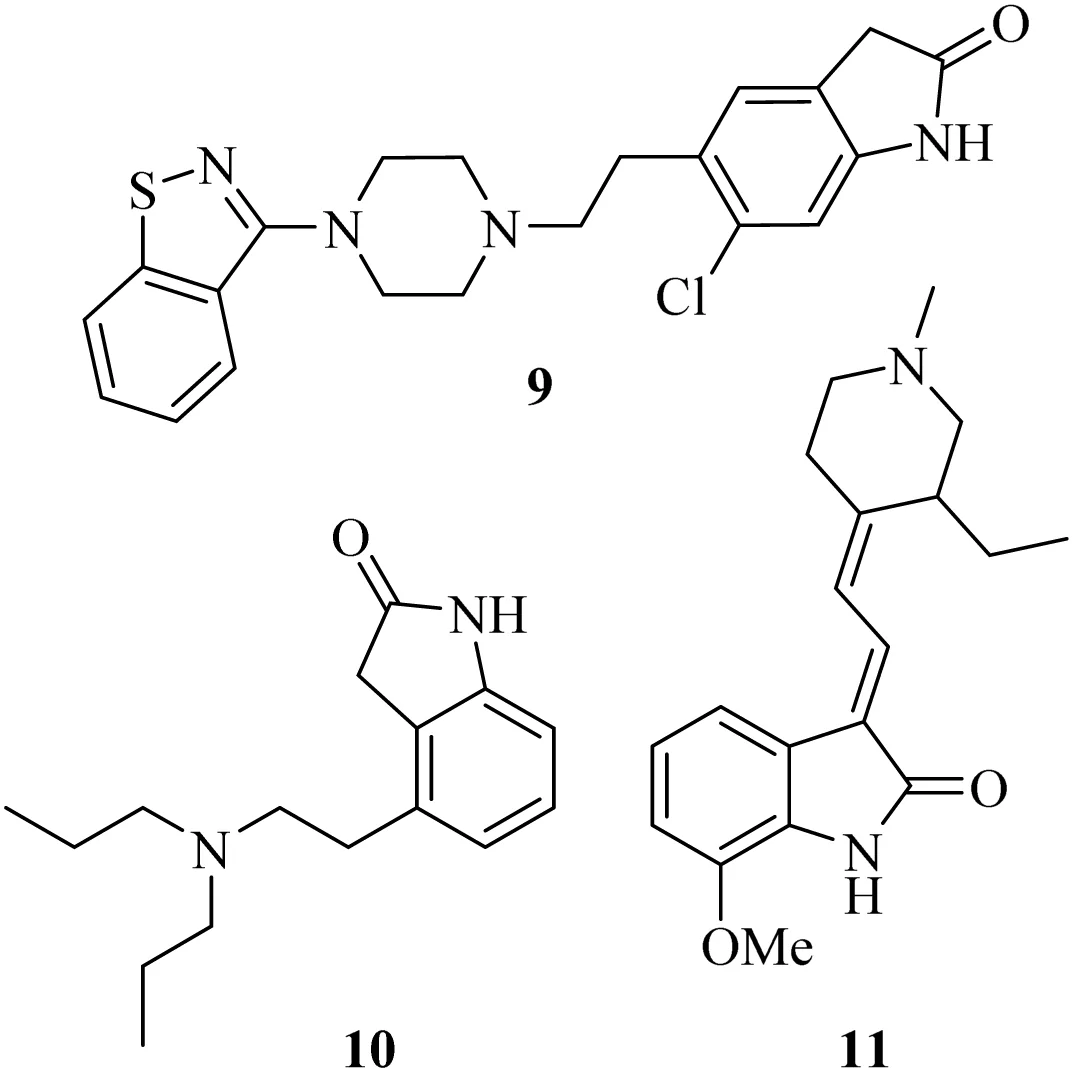
Oxindole-based marketed drugs.

The structure of oxindoles, particularly those that are spiro-fused to other cyclic frameworks, has attracted significant attention from researchers in the fields of organic synthesis and medicinal chemistry.^25^

Compounds containing at least two rings that share a single atom are called spiro compounds, which can be either carbocyclic or heterocyclic. The spiro atom refers to a single shared atom that links two rings within a spirocyclic framework.^4^ Spirooxindole compounds are a diverse class of heterocyclic compounds characterized by a spiro junction involving an oxindole moiety. These compounds have attracted significant interest owing to their structural complexity and wide-ranging applications.^26^

Spirooxindoles exhibit a variety of biological activities, including anticancer,^27^ antimicrobial 12,^28^ antitumor 13,^29^ and antifungal 14 (ref. 30) properties (Fig. 3). Their biological efficacy is often attributed to their ability to interact with diverse biological targets, such as enzymes, receptors, and DNA, making them valuable in drug discovery and development.^31^ Furthermore, their unique stereochemistry and pharmacophore diversity enable researchers to fine-tune their properties for specific therapeutic applications.^32^

**Fig. 3:**
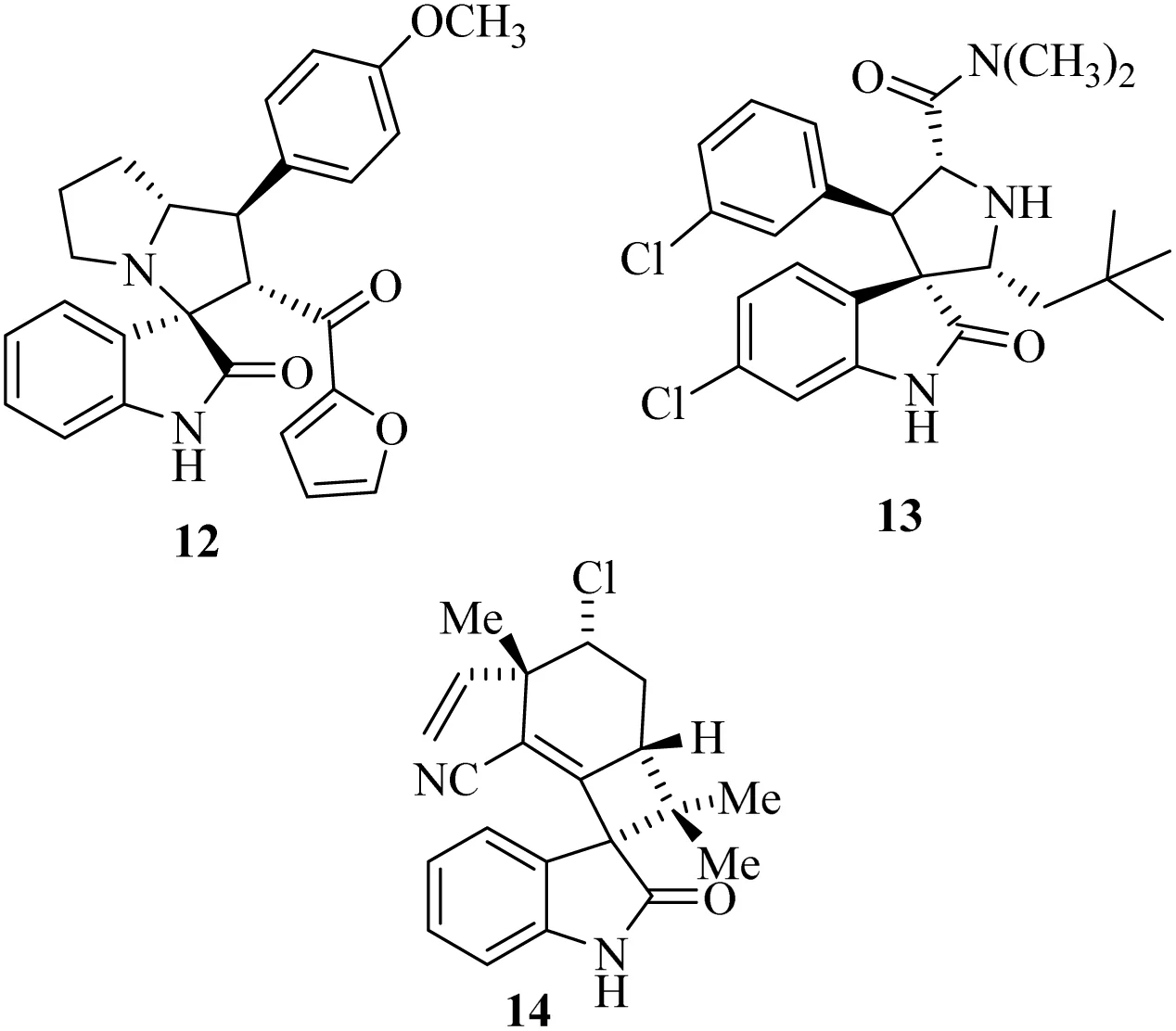
Biologically active spirooxindole chemical structures.

The presence of multiple quaternary stereocenters within bisspiro systems leads to highly complex and conformationally constrained structures. This distinct spatial arrangement not only increases molecular diversity but also plays a crucial role in modulating interactions with biological targets. As a result, bisspiro compounds have emerged as valuable motifs in medicinal chemistry and drug discovery, frequently exhibiting promising pharmacological profiles, including anticancer, antimicrobial, and antiviral activities.^33–36^

Among the numerous reported bisspiro architectures, bisspirooxindole derivatives are especially noteworthy. From a synthetic perspective, the construction of bisspirooxindole frameworks remains a challenging yet highly active area of research. The simultaneous formation of multiple stereocenters and spiro junctions often requires the development of efficient and selective synthetic methodologies. Consequently, various strategies, such as cycloaddition reactions, cascade cyclizations, and transition-metal-catalyzed transformations have been developed to access these structurally complex architectures.

The growing interest in bisspirooxindole chemistry is reflected in the increasing number of publications devoted to the development of synthetic methods for these valuable heterocyclic scaffolds. As illustrated in Fig. 4, the number of publications reporting synthetic approaches to bisspirooxindoles has varied over the period 2006–2026, highlighting sustained research interest in the synthesis and functionalization of this these important structural motifs. Accordingly, this review provides an overview of the reported synthetic methods for the preparation of bisspirooxindole frameworks, with particular emphasis on their reaction patterns, substrate scope, and synthetic advantages and limitations.

**Fig. 4:**
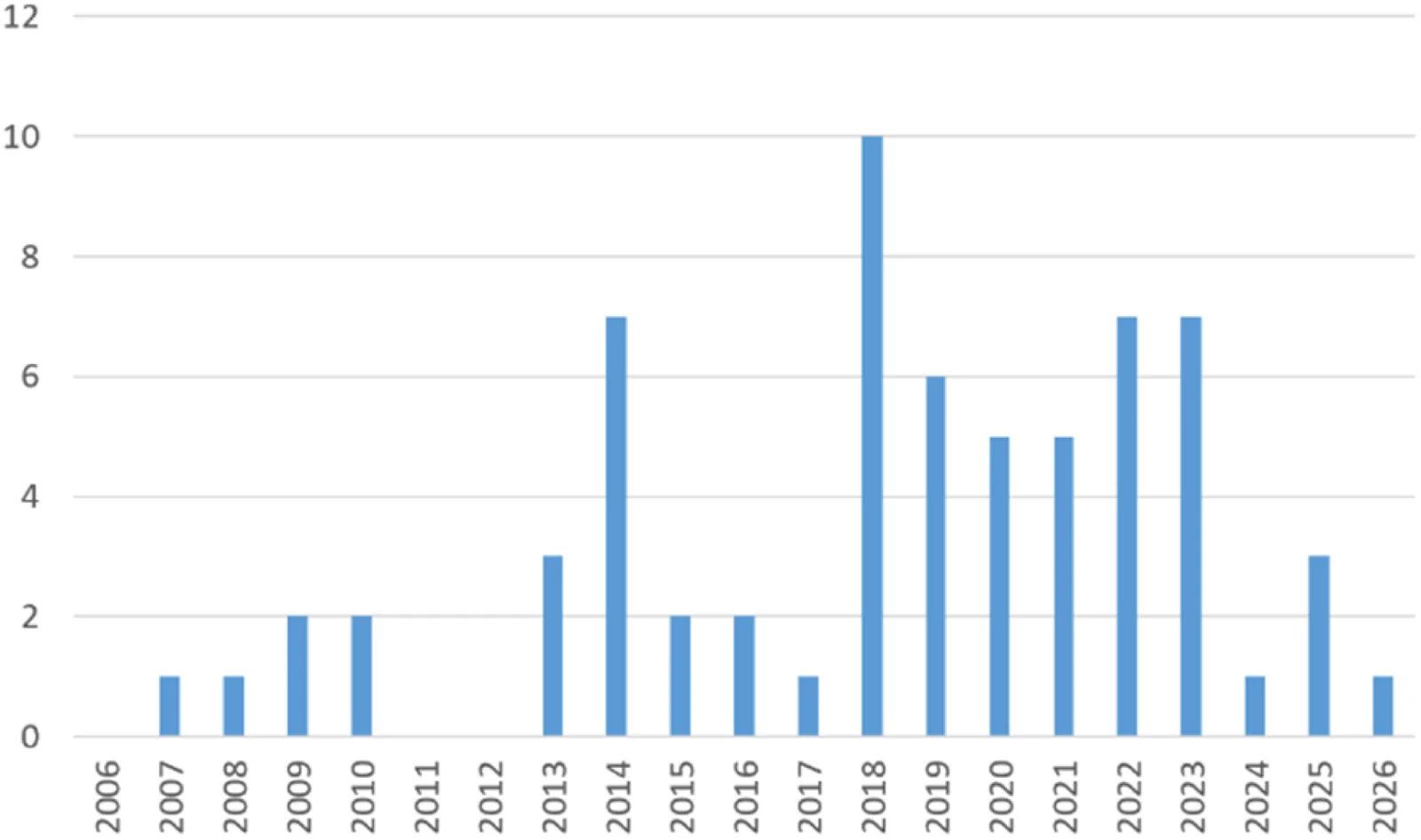
Number of publications on the synthesis of bissoxindoles from 2006 to 2026.

Although several reviews have addressed the synthesis of spirooxindoles from different perspectives, including stereoselective synthesis, transition-metal catalysis, and reaction-based approaches, bisspirooxindoles have generally been discussed only as selected examples or as a subset of the broader spirooxindole family. Consequently, a dedicated analysis of the synthetic approaches developed for the construction of bisspirooxindole frameworks remains lacking. This gap is particularly relevant given the structural complexity of bis-spirooxindoles, which commonly contain two spiro units, multiple rings and stereogenic centers, requiring diverse and strategically distinct synthetic approaches. To address this gap, the present review provides, to the best of our knowledge, the first dedicated and systematic overview focused specifically on the synthesis of bisspirooxindoles. In contrast to previous reviews that have primarily organized spirooxindole chemistry according to reaction type, catalytic system, stereochemical outcome, or ring size, the present review adopts a starting-material-oriented classification specifically designed to elucidate the synthetic approaches used for constructing bisspirooxindole frameworks (Table 1).^37–41^ The reported methodologies are categorized according to the nature and structural role of the starting materials and discussed in terms of their underlying synthetic strategies, reaction scope, stereochemical features, structural diversity, and, where appropriate, their advantages and limitations. This organization provides a unified framework for comparing otherwise dispersed synthetic methodologies and highlights the diverse strategies that have been developed for the construction of bisspirooxindole architectures.

**Table 1:** Overview of representative reviews on spirooxindole chemistry and comparison of their scope with the present systematic review of bisspirooxindole synthesis

Previous review	Year	Main focus	Treatment of bis-spirooxindoles	Distinctive feature of the present review
Ball–Jones *et al.*	2012	Enantioselective synthesis	Selected examples	Dedicated focus on bis-spirooxindoles and diverse synthetic strategies
Boddy & Bull	2021	Stereoselective synthesis	Part of broader spirocyclic oxindole chemistry	Exclusive focus on bis-spirooxindole synthesis
Saranya *et al.*	2021	Transition-metal catalysis	Within broader catalytic spirooxindole chemistry	Metal- and metal-free approaches classified by starting materials
Liandi *et al.*	2024	Green synthesis	Selected examples	Comprehensive coverage beyond green-synthesis approaches
Rostami *et al.*	2025	Recent synthetic advances	Within broader spirooxindole scope	Starting-material- and strategy-based analysis of bis-spirooxindoles
Present review	2026	Bis-spirooxindole synthesis	Dedicated and comprehensive	Starting-material-oriented classification + synthetic-strategy analysis

As shown in Fig. 5, the reported synthetic approaches to bisspirooxindoles are distributed among several classes of starting materials. Among these, azomethine ylide-based strategies represent the largest proportion of the reported studies, followed by 3-alkenyl oxindole annulation strategies and isatin-derived MBH carbonate approaches. This distribution suggests that certain classes of starting-material have received greater attention in the development of bisspirooxindole synthesis. The individual synthetic strategies associated with each class are discussed in the following sections.

**Fig. 5:**
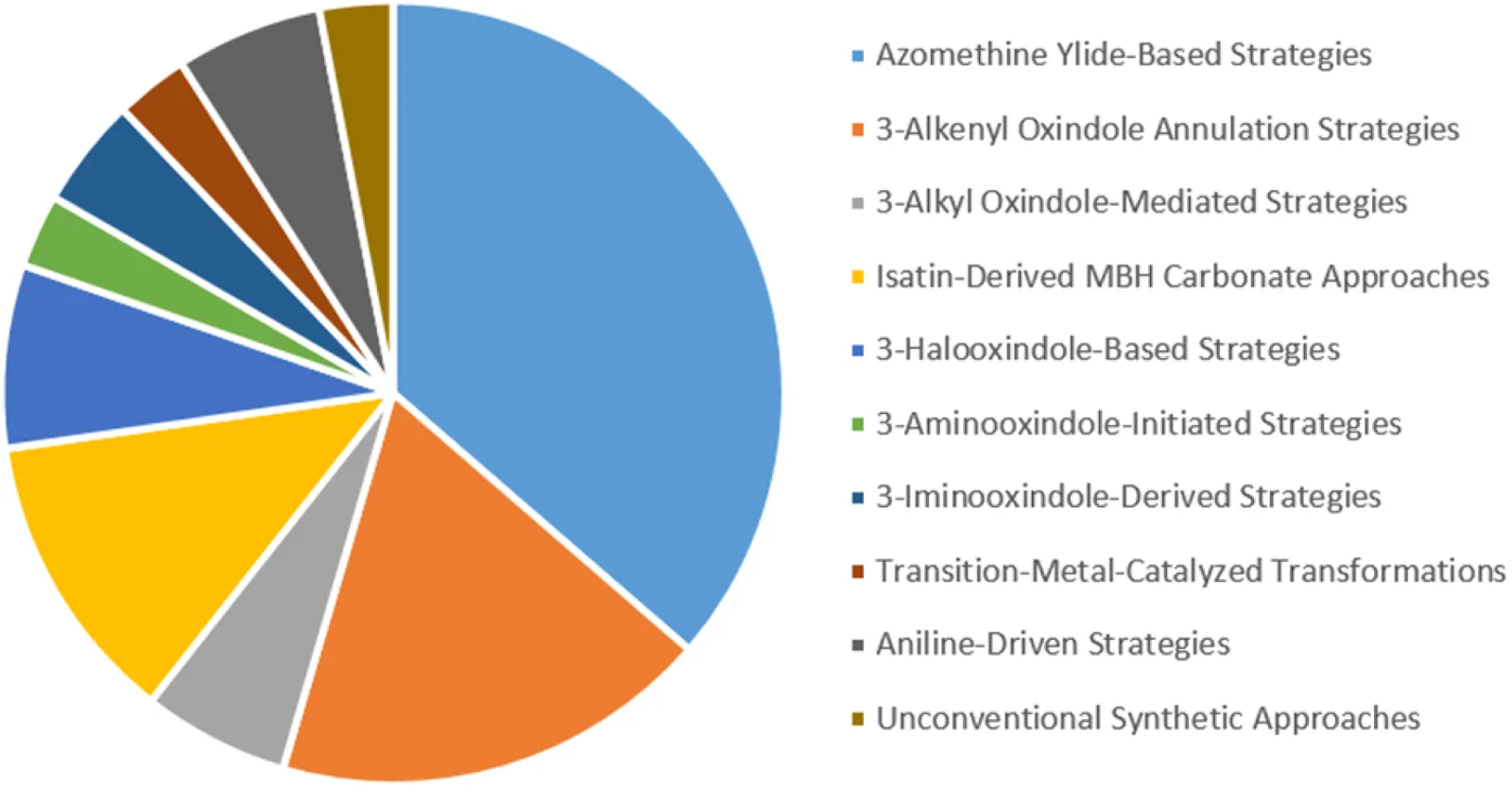
Distribution of publications by synthetic strategy for bisspirooxindole synthesis.

## Azomethine ylide-based strategies

The synthesis of bisspirooxindole derivatives from isatin and amino acides/amines represents one of the most versatile and widely explored approaches for the construction of the bisspirooxindole framework. The broad availability of amino acides and amines, together with their ability to generate reactive intermediates, has enabled the development of numerous efficient synthetic methodologies. Owing to their operational simplicity, substrate diversity, and generally high synthetic efficiency, azomethine ylide-based strategies have attracted considerable attention and continue to serve as valuable tools in modern bisspirooxindole synthesis.

Very recently, an efficient three-component domino reaction of isatin 15, pipecolic acid 16, and alkyl 2-(1-methyl-2-oxoindolin-3-ylidene) acetate 17 for the synthesis of bisspirooxindole–hexahydroindolizines 18 as potential anticancer agent reported by our research group (Scheme 2).^42^ This strategy provided a simple and convenient route to synthesis of novel methyl-2,2″-dioxo-1′,5′,6′,7′,8′,8a′-hexahydrodispiro[indoline-3,2′-indolizine-3′,3″-indoline]-1′-carboxylate derivatives. In this strategy, the azomethine ylide, generated *in situ* from the condensation of an amino acide 16 with isatin, immediately reacts with alkyl 2-(1-methyl-2-oxoindolin-3-ylidene)acetate 17 to afford the desired product without isolation of the intermediate. Initial biological evaluation revealed that the synthesized compounds exhibited significant cytotoxic activity against MCF-7 breast cancer cells. The docking study suggested that these compounds can act as an estrogen receptor α (ER-α) modulator, and can be considered a promising lead compound.

**Scheme 2:**
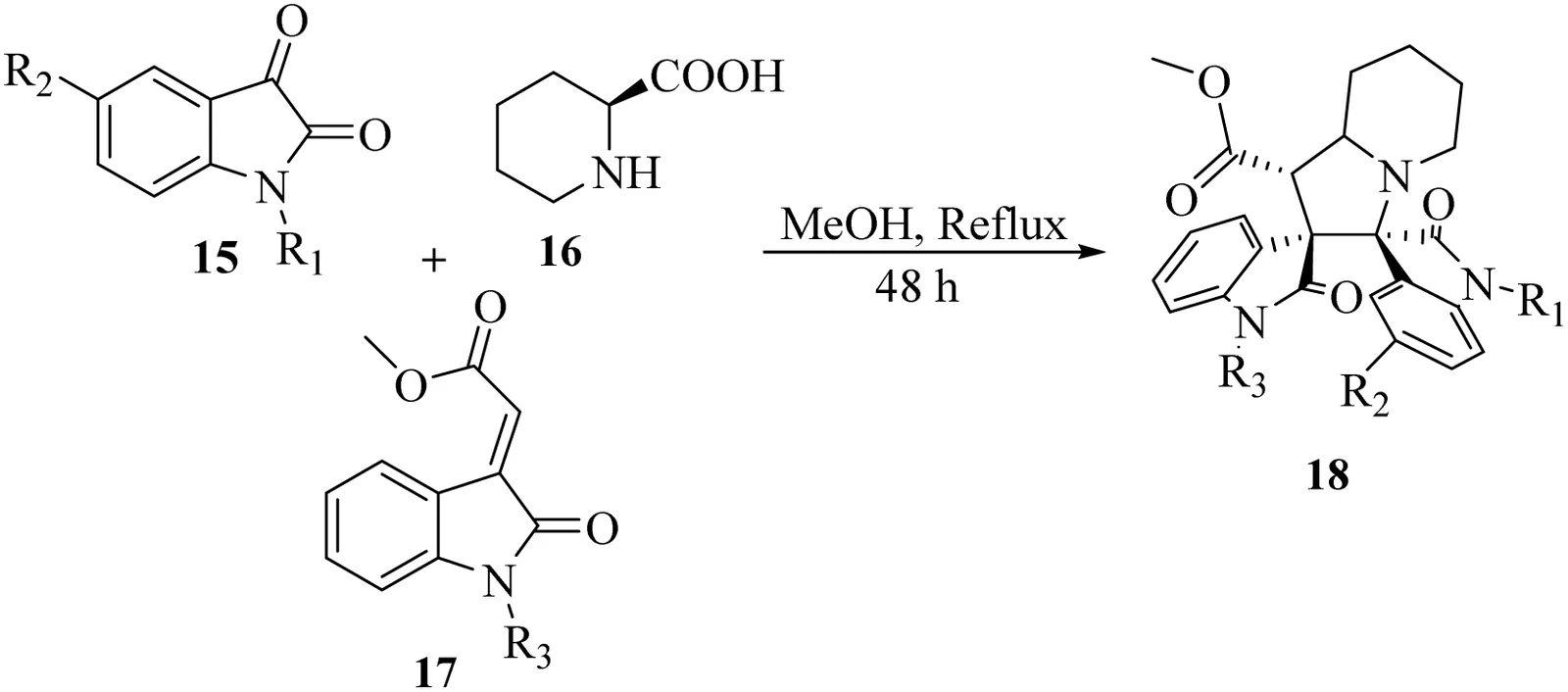
Regio- and diastereoselective synthesis of bisspirooxindole-hexahydroindolizines as potential anticancer agents.

Recently, Pronina *et al.* developed a three-component [3+2]-cycloaddition reaction of azomethine ylides, derived *in situ* from isatins 15 and azetidine-2-carboxylic acid 19, with different maleimides 20 and itaconimides 22, enabling one-pot access to spiro[1-azabicyclo[3.2.0]heptane] frameworks 21, 23 (Scheme 3).^43^ The protocols possess a broad substrate scope and good diastereo- and regioselectivity. DFT calculations revealed that 1,3-dipolar cycloaddition of azomethine ylides to maleimides and itaconimides proceeds through a single-step, concerted mechanism. The remarkable regio- and diastereoselectivity observed in these reactions was attributed to secondary orbital interactions based on DFT calculations. Moreover, these new polyheterocyclic systems demonstrated the antiproliferative effects against the K-562 and HeLa cancer cell lines.

**Scheme 3:**
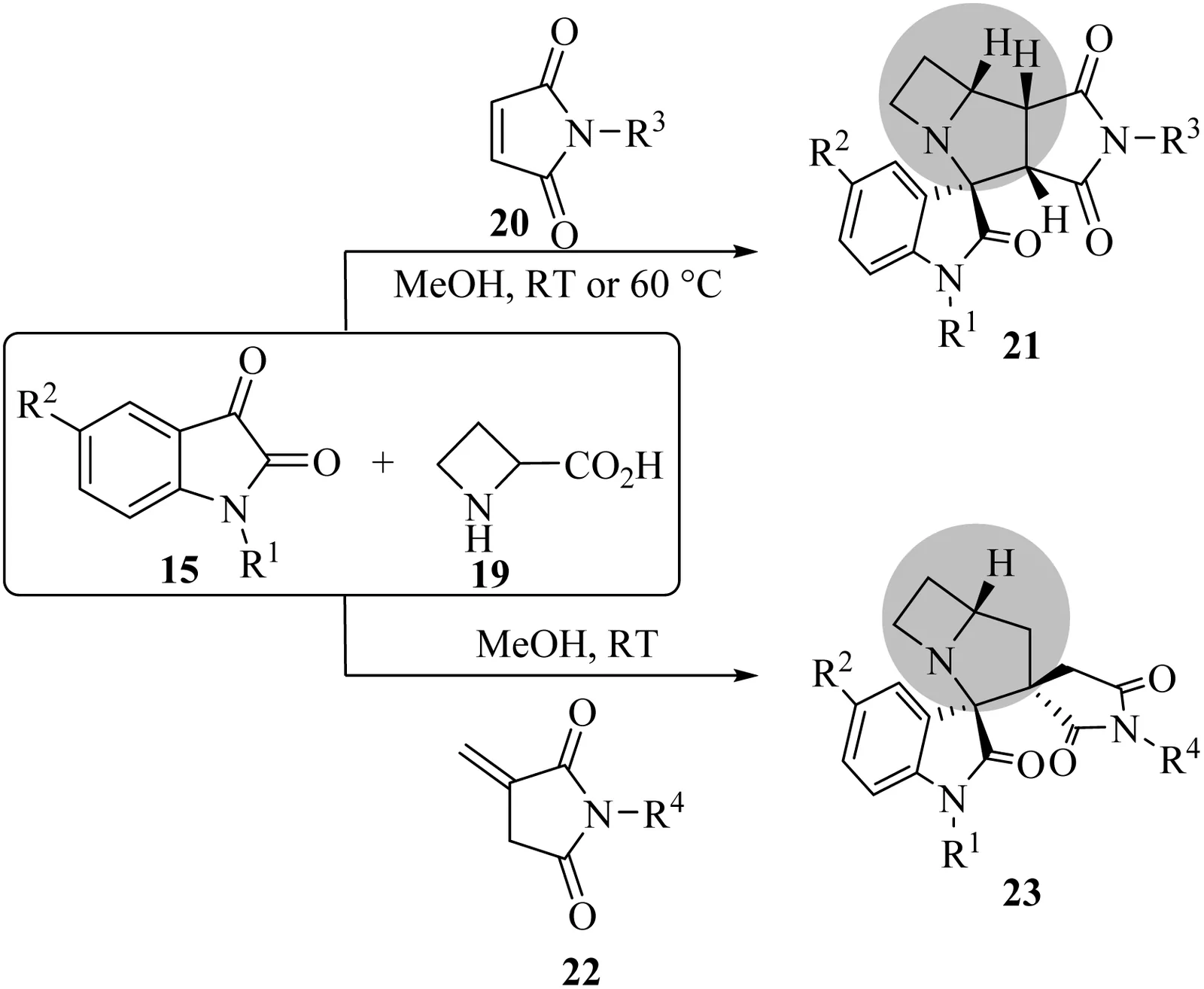
A three-component [3+2]-cycloaddition reaction for the synthesis of spiro[1-azabicyclo[3.2.0]heptane] frameworks.

In 2023, singh *et al.* extracted *parthenin*24 from *Parthenium hysterophorus* as a source of a double bond for the synthesis of bisspiro compounds.^44^ This natural compound possesses a range of valuable biological activities, including antileishmanial, anticancer, anti-inflammatory, antibacterial, antiviral, and trypanocidal activities.^45–47^*Parthenin*, which contains two double bonds, was utilized as a dipolarophile in the reaction with isatin 25 and l-proline 26. Initially, l-proline reacted with isatin to generate an azomethine ylide, which subsequently underwent a 1,3-dipolar cycloaddition reaction with the exocyclic double bond of *parthenin*, affored to a bisspiro-pyrrolizidino-oxindole 27. The reaction progressed under mild conditions in a chloroform/methanol solvent system and exhibited regio- and diastereoselectivity. Furthermore, the choice of amino acid influenced the reaction pathway, with l-proline and l-pipecolinic acid 16 favoring α-addition, while thioproline 29 and sarcosine 31 led to β-addition (Scheme 4).

**Scheme 4:**
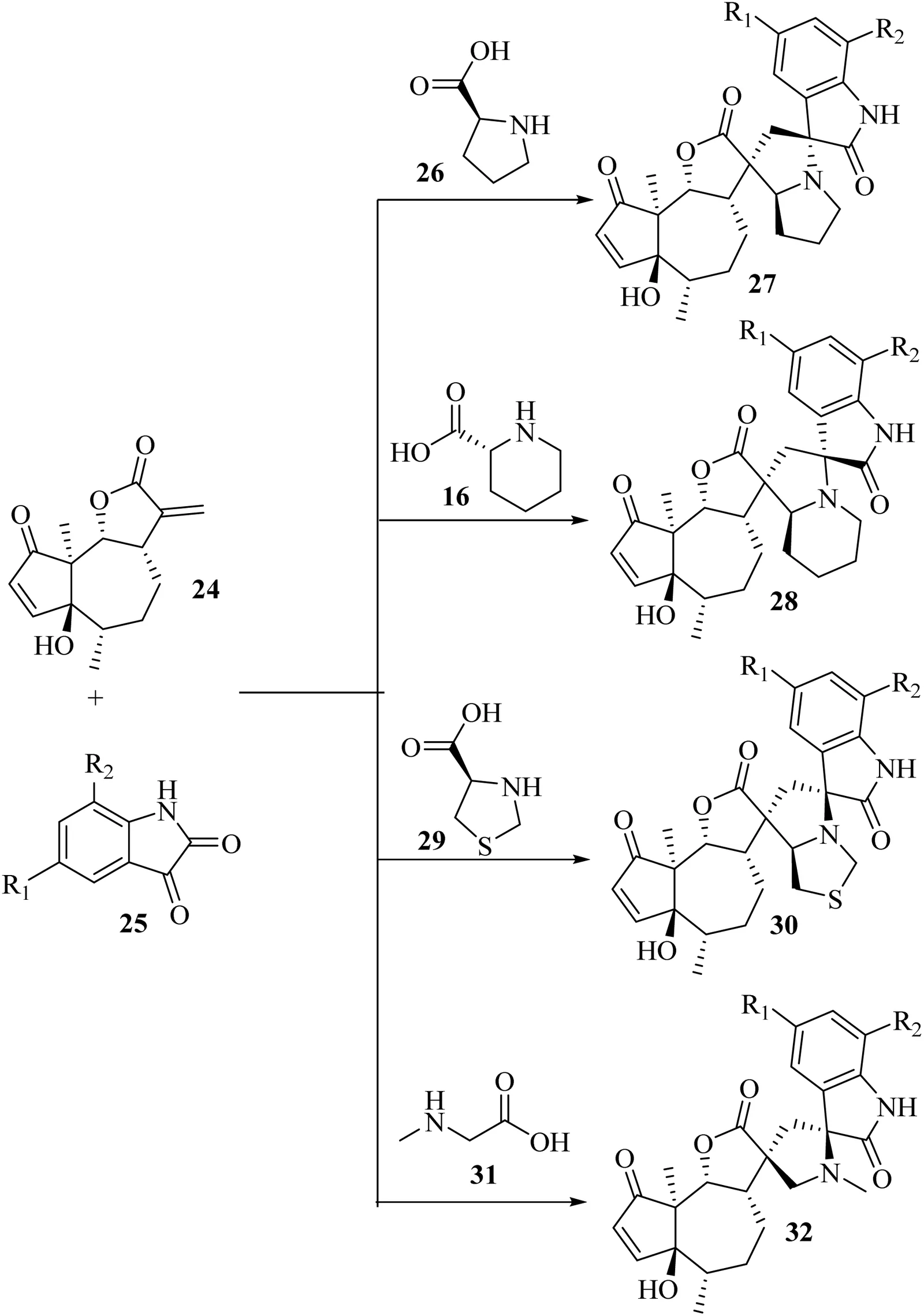
Synthesis of novel bisspirooxindole hybrids of parthenin.

In 2022, De Silva, Maniam, and colleagues aimed to synthesize bisspirooxindole derivatives that could inhibit amyloid plaques formation, exhibit low toxicity, and efficiently penetrate the brain.^48^ Previous studies have shown that spirooxindole derivatives act as acetylcholineesterase inhibitors, a key therapeutic target in Alzheimer's disease. Inspired by Liu *et al.*^49^ who extracted spiropyrrolizidine oxindole alkaloids derivatives from leaves of *Isatis indigotica* and demonstrated 72–86% inhibition of Aβ fibril formation, the researchers in this project^48^ designed bisspirooxindole pyrrolizidine derivatives with high brain permeability and topological polar surface area below 90 Å^2^. The synthesis was performed using a one-pot, microwave-assisted 1,3-dipolar cycloaddition, where azomethine ylide was generated *in situ* from isatin 6 and l-proline 26, while the dipolarophile 33 was obtained *via* an aldol reaction. The process exhibited moderate yield, high stereospecificity, and regioselectivity, and required no complex purification steps.

The resulting compounds were able to interact with the amyloidogenic region within the peptide, thereby preventing the formation of abnormal amyloid structures (Scheme 5).

**Scheme 5:**
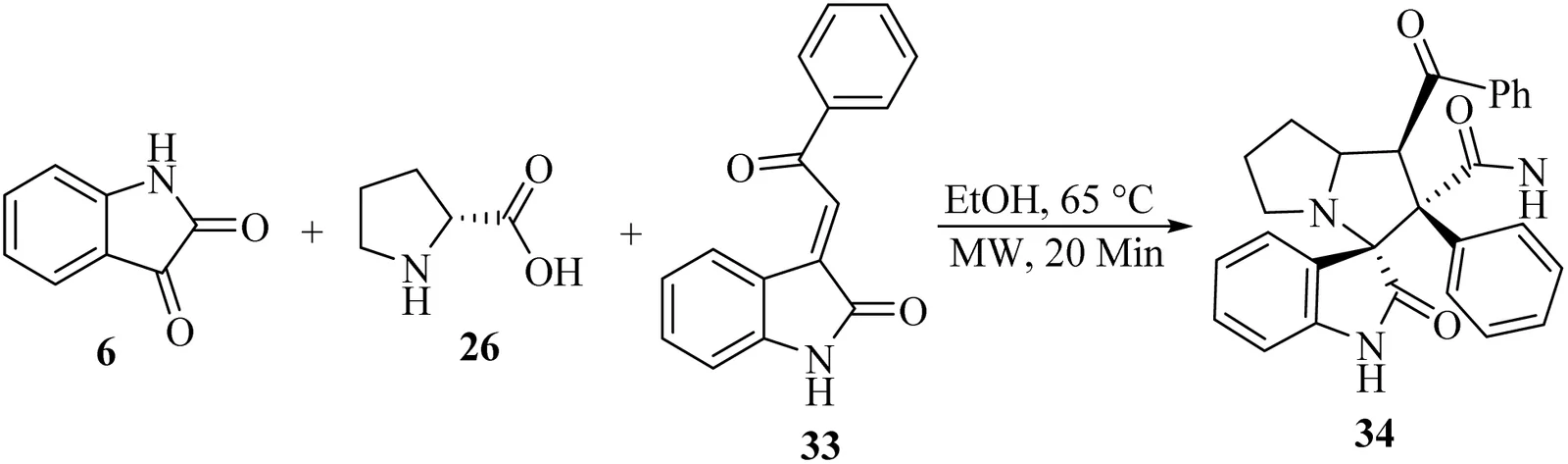
Microwave-assisted 1,3-dipolar cycloaddition synthesis of bisspirooxindole compounds.

In a study conducted by the Toumi research group in 2021, a novel synthetic strategy was developed for the construction of bisspiro compounds by integrating two bioactive heterocyclic moieties, oxindole and pyrrolo[1,2-*a*]isoquinoline derivatives.^50^ Both scaffolds are widely found in natural products and exhibit diverse pharmacological activities, including cytotoxic activity toward several tumor cell lines,^51^ human DNA topoisomerase I inhibition,^52^ HIV-1 integrase inhibiting reagents,^53^ and neuroprotective activity for the prevention and treatment of Parkinson's disease.^54^ Inspired by previous studies, such as those by Huang^55^ and Kumar,^56^ which employed 1,3-dipolar cycloaddition reactions involving azomethine ylides derived from isatin 6 and 1,2,3,4-tetrahydroisoquinoline (THIQ) 35 (Scheme 6), the authors expanded on this approach by incorporating a third component (Z)-5-arylidene-1,3-thiazolidine-2,4-diones 37.^50^ This compound, known for its antidiabetic^57–59^ potential (*e.g.*, pioglitazone and rosiglitazone), was used in a three-component reaction with 5-bromo-isatin and THIQ.

**Scheme 6:**
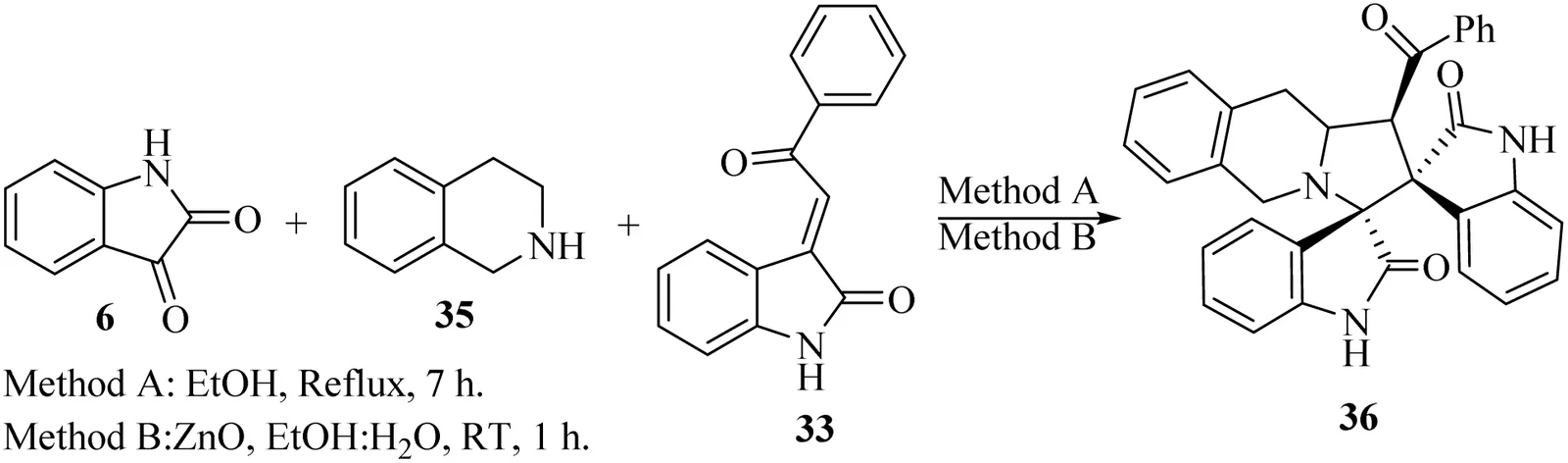
Diastereoselective synthesis of bisspirooxindole.

The reaction did not proceed at room temperature; however, increasing the reaction temperature led to the formation of bisspiropyrrolo[1,2-*a*] isoquinoline-oxindoles compound as a mixture of two regioisomers. Two optimized conditions were identified. When the reaction was carried out in acetonitrile under reflux at 60 °C, the endo regioisomer was obtained predominantly in 79% yield. In contrast, the use of dioxane resulted in a notable shift in regioselectivity toward the alternative regioisomer, underscoring the significant influence of the solvent on the reaction outcome. Moreover, the influence of electronic substituents on the arylidene group was studied: electron-donating groups such as methyl and methoxy improved reaction yield but reduced regioselectivity, whereas electron-withdrawing groups, such as fluoro, chloro, and bromo enhanced both yield and regioselectivity (Scheme 7).

**Scheme 7:**
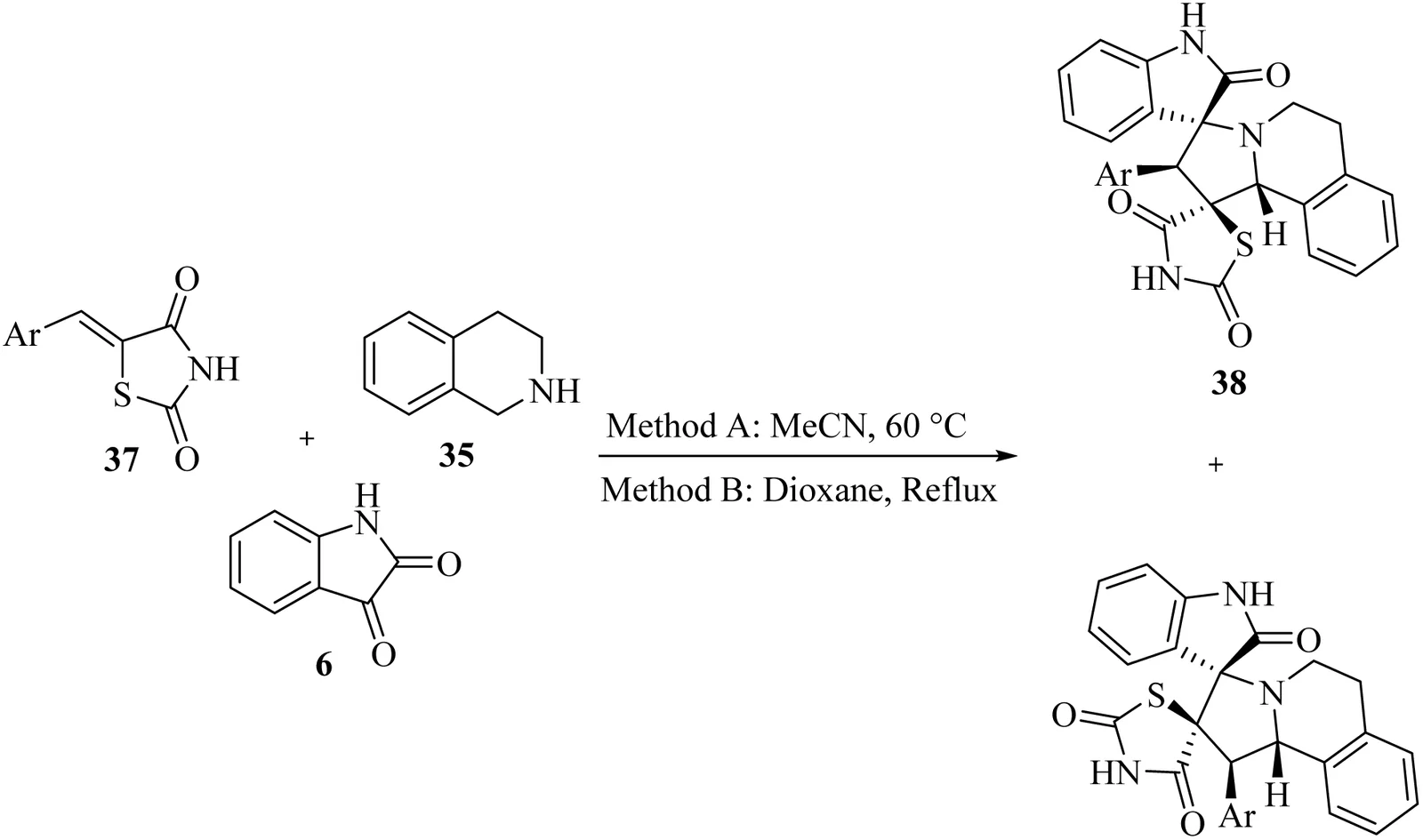
Diversity-oriented synthesis of bisspiropyrrolo[1,2-*a*]isoquinoline derivatives.

In a related study, a modified dipolarophile, namely Z-4-arylidene-thioxothiazolidine-2,4-dione, in which one thiocarbonyl (C=S) group was replaced by a one carbonyl group was employed. Its reaction with azomethine ylide, derived from isatin and THIQ, resulted in the formation of a novel bisspiro compound through a regioselective [3+3] cycloaddition, accompanied by formal extrusion of H_2_S.^50^

In a study published in 2020, Shahrestani *et al.* reported a diastereoselective one-pot protocol for the synthesis of bisspiro compounds using a chiral auxiliary. Their approach involved isatin 15 with amino acids such as proline 26 or sarcosine 31 to generate azomethine ylides *in situ*, along with a dipolarophile specifically, 2-oxindole-3-ylideneacetylsultam 40. These ylides underwent highly regioselective [3+2] cycloaddition reactions *via* the endo pathway, enabling the efficient construction of structurally complex bisspiro[pyrrolizidine/pyrrolidine] derivatives 41 containing four stereogenic centers. Reaction parameters were systematically optimized, revealing that polar protic solvents, especially methanol under reflux conditions, substantially improved the yield and efficiency of the transformation. Under these optimized conditions, the desired products were obtained in excellent yields of up to 96% within just 30 minutes (Scheme 8).^60^

**Scheme 8:**
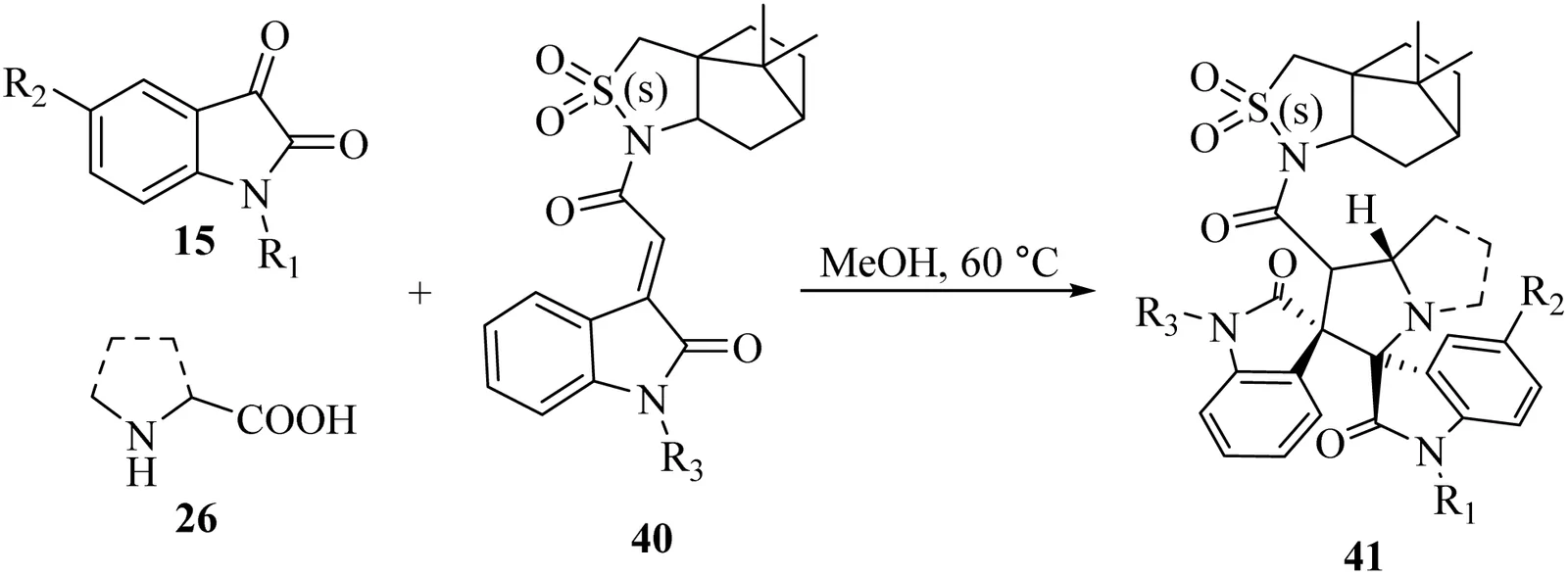
Asymmetric [3+2] cycloaddition reaction for the synthesis of enantioenriched bisspirooxindole pyrrolizidine/pyrrolidines.

In 2020, Kumar and his team investigated a one-pot, three-component cycloaddition reaction involving fluorinated isatins 42, tryptophan 43 or phenylalanine 44, and 2-arylmethylidene-5,6-dimethoxyindenones 45 in 1-butyl-3-methylimidazoliumbromide ([bmim]Br) as a green solvent, affording the desired products in moderate to good yields. The resulting bisspiro compounds 46 showed significant inhibitory activity against acetylcholinesterase (AChE) and butyrylcholinesterase (BChE), which are key enzymes involved in Alzheimer's disease. Notably, compounds incorporating an indole subunit exhibited enhanced inhibitory activity (Scheme 9).^61^

**Scheme 9:**
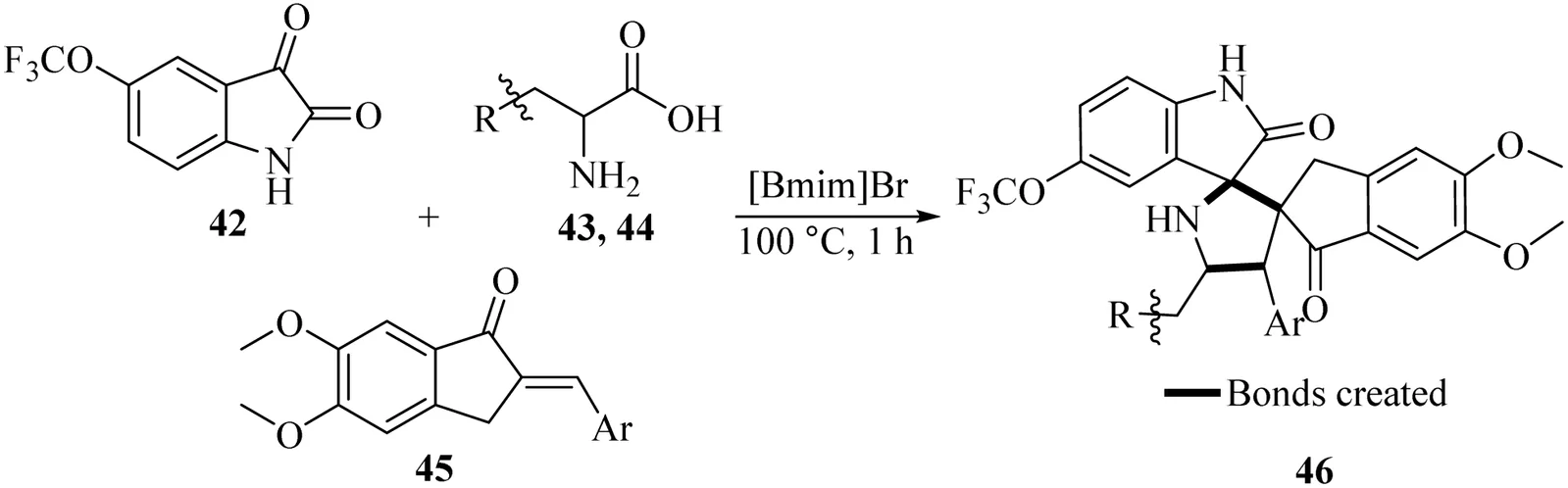
Synthesis of fluorinated bisspiropyrrolidines in [bmim]Br.

In 2020, Yuvaraj and his coworkers reported the endo-selective synthesis of bisspiro[3,2′-pyrrolidinyl]oxindole 48 using a one-pot, catalyst-free, and environmentally friendly approach that generates no by-products, while providing high yields. The reaction employs a three-component system comprising isatins 6, sarcosine 31, and 2-oxindole-3-ylidene 47 in acetonitrile (Scheme 10).^62^ Notably, the choice of solvent plays a crucial role in enhancing the reaction yield. Aprotic polar solvents, such as acetonitrile, afford the cycloaddition products in good to high yields, whereas protic polar solvents provide only moderate to good yields. This difference is attributed to the superior solubility of the dipolarophile in aprotic solvents like acetonitrile compared with protic solvents. This method is suitable for the synthesis of various bisspirooxindole derivatives, which show promising anticancer potential.

**Scheme 10:**
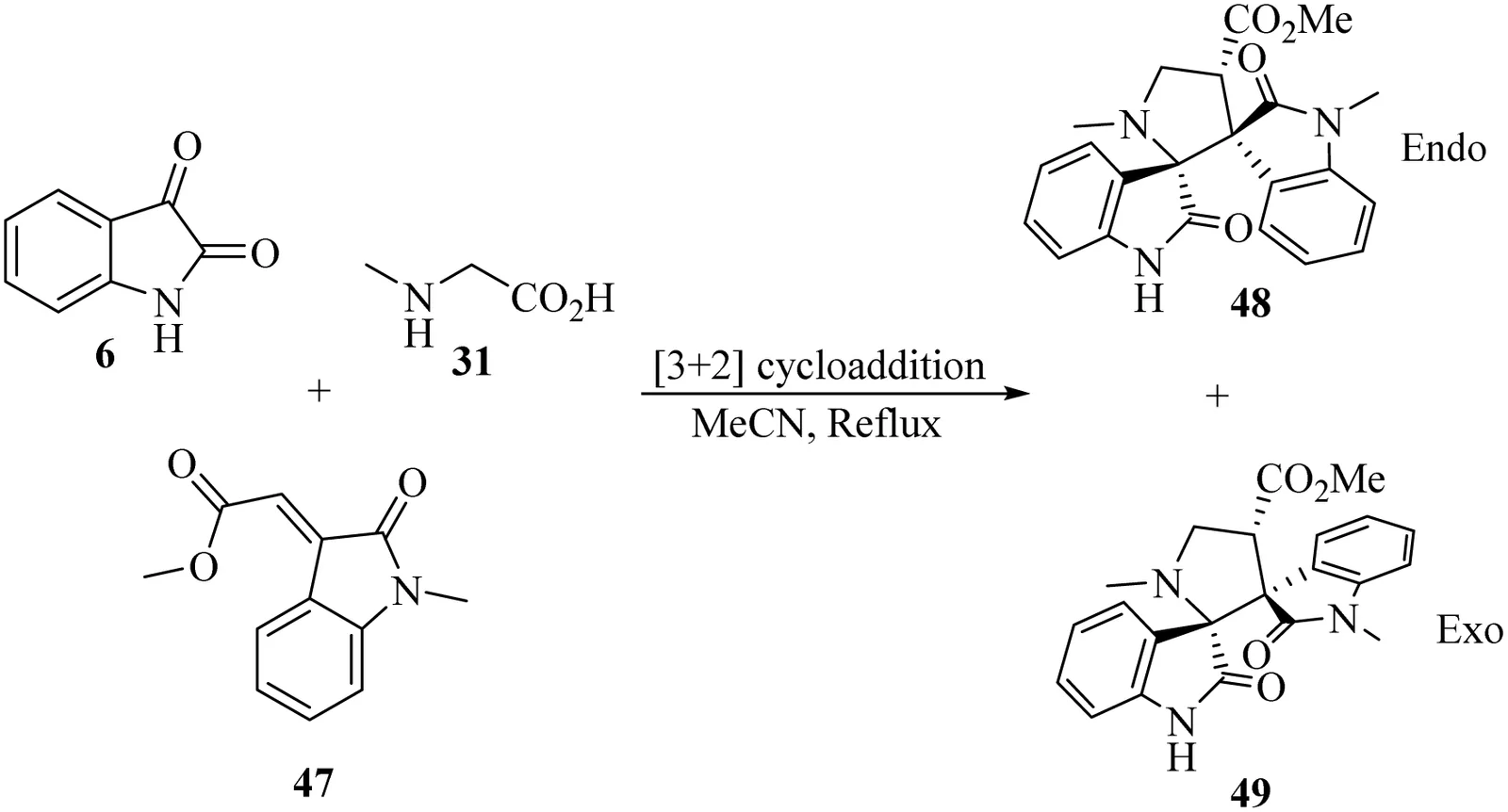
Catalyst-free synthesis of bisspiropyrrolidineoxindole.

In 2019, the regioselective synthesis of bisspiropyrrolidinyl-oxindole-based alkaloid derivatives 52 was developed by Srour *et al.* The synthesis was conducted in refluxing ethanol through the *in* situ formation of an azomethine ylide, generated *via* the decarboxylative condensation of isatin 50 and sarcosine 31, followed by a [3+2] cycloaddition with an α,β-unsaturated ketone 51. Biological evaluation of the synthesized compounds revealed their potential as inhibitors of acetylcholinesterase (AChE) and butyrylcholinesterase (BChE) enzymes (Scheme 11).^63^

**Scheme 11:**
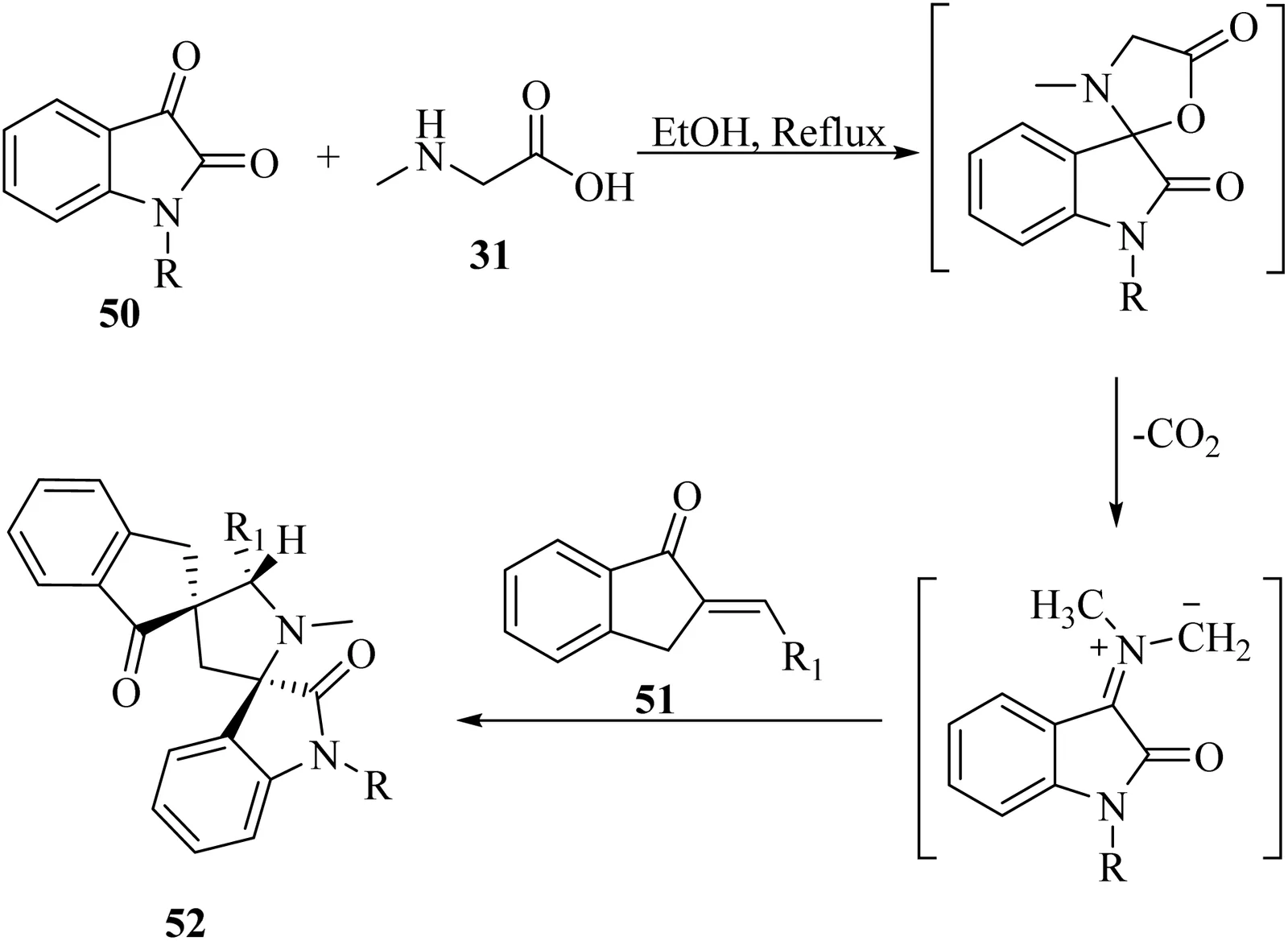
Synthesis of bisspiro[indene-2,3′-pyrrolidine-2′,3″-indoline]-1,2″(3H)-dione *via* [3+2] cycloaddition reaction.

It was found that the presence of an ethyl group on the oxindole ring, combined with an unsubstituted aromatic ring, such as phenyl or thienyl, enhanced the inhibitory activity. In contrast, substituted aromatic rings reduced the activity.

Bhandari and his coworkers, in 2019, developed an efficient and environmentally friendly method for the synthesis of bisspirooxindole scaffolds, offering excellent yields, high atom economy, and producing only non-toxic by-products such as CO_2_ and H_2_O. The reaction proceeds efficiently under microwave irradiation in ethanol, without the need for any additives. The process involves the *in situ* generation of azomethine ylides through the decarboxylative condensation of isatin derivatives 15 and primary α-amino acids 53, followed by a [3+2] cycloaddition with 3-alkenyl oxindoles 54 (Scheme 12).^64^

**Scheme 12:**
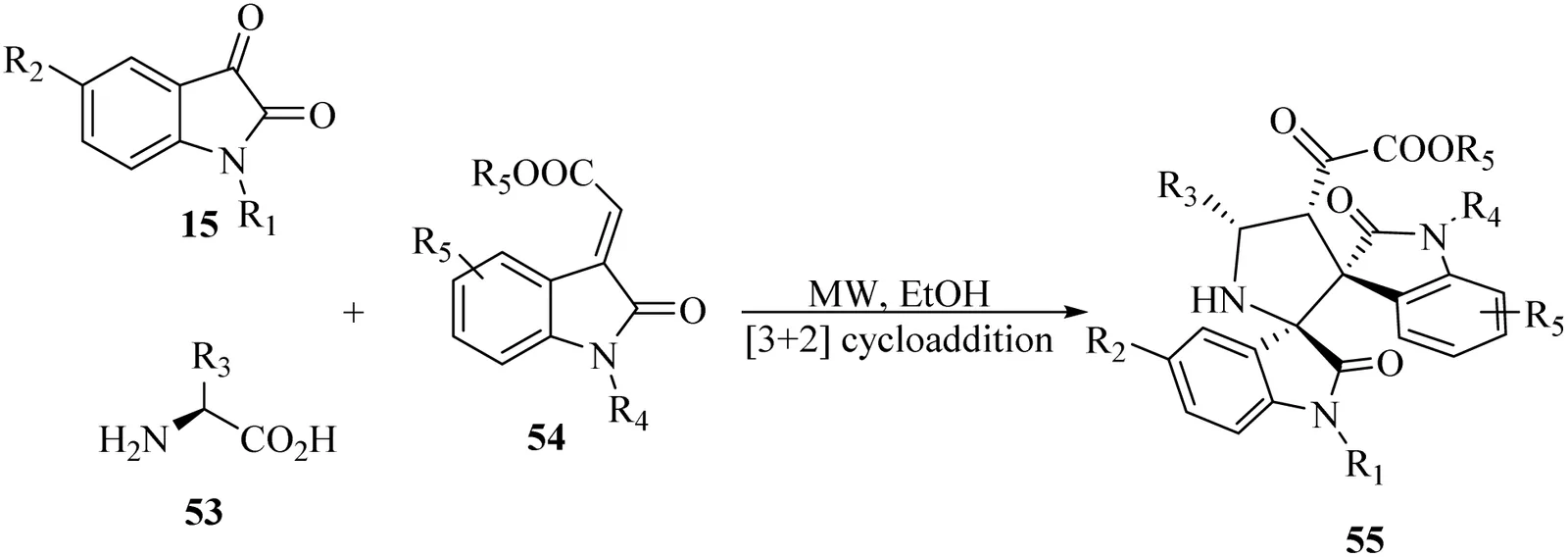
Microwave-assisted strategy for the construction of bisspirooxindole.

This approach provides a practical and effective strategy for forming both C–C and C–N bonds, leading to products with four stereocenters and a single diastereomer. Moreover, it enables the construction of a structurally diverse bisspirooxindole library.

A combinatorial stereoselective synthesis of novel spiro pyrazolo [3,4*b*] quinoline–pyrrolidine/pyrrolothiazole/indolizine–oxindole/acenaphthene hybrid heterocycles *via* multicomponent dipolar cycloaddition strategy was developed by Sumesh *et al.* In this strategy 1,3-dipolar cycloaddition of azomethine ylides generated *in situ* from α-amino acids (16, 29, 31) and 1,2-diketones (6, 57) with dipolarophiles 56 afforded pyrazolo[3,4-*b*]quinoline derivatives (58–63), in quantitative yields (Scheme 13).^65^ The generation of diverse azomethine ylides by the decarboxylative condensation of sarcosine 31, thiazolidine-4-carboxylic acid 29, or piperidine-2 carboxylic acid 16 and isatin 6 or acenaphthenequinone 57 enabled the synthesis of a wide variety of the desired bisspiro products (58–63) through cycloaddition reactions with exocyclic alkenenic dipolarophiles.

**Scheme 13:**
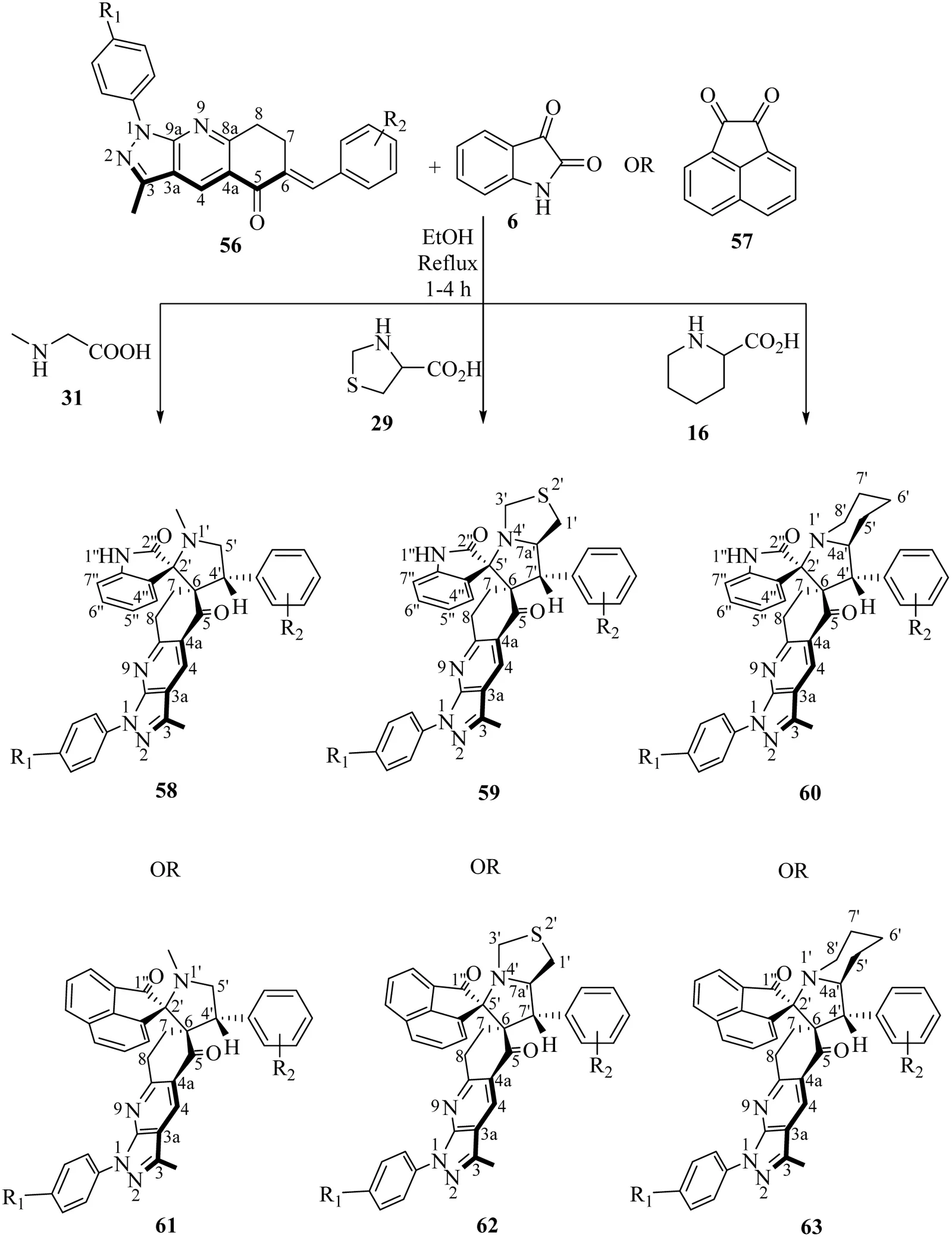
Combinatorial synthetic routes towards bisspiro tethered pyrazolo[3,4-*b*]quinoline hybrid heterocycles.

Dai and his coworkers, in 2015, reported, for the first time, a method for the synthesis of the 3,3′-pyrrolidinyl bisspirooxindole scaffold 66*via* an organocatalytic asymmetric 1,3-dipolar cycloaddition reaction between azomethine ylides and methyleneindolinones 64. This strategy enables the construction of bisspirooxindoles bearing three adjacent and two quaternary stereogenic centers, affording the corresponding products in high yields (up to 99%) and with excellent diastereo- and enantioselectivities (up to >95 : 5 dr and 98% ee) (Scheme 14).^66^

**Scheme 14:**
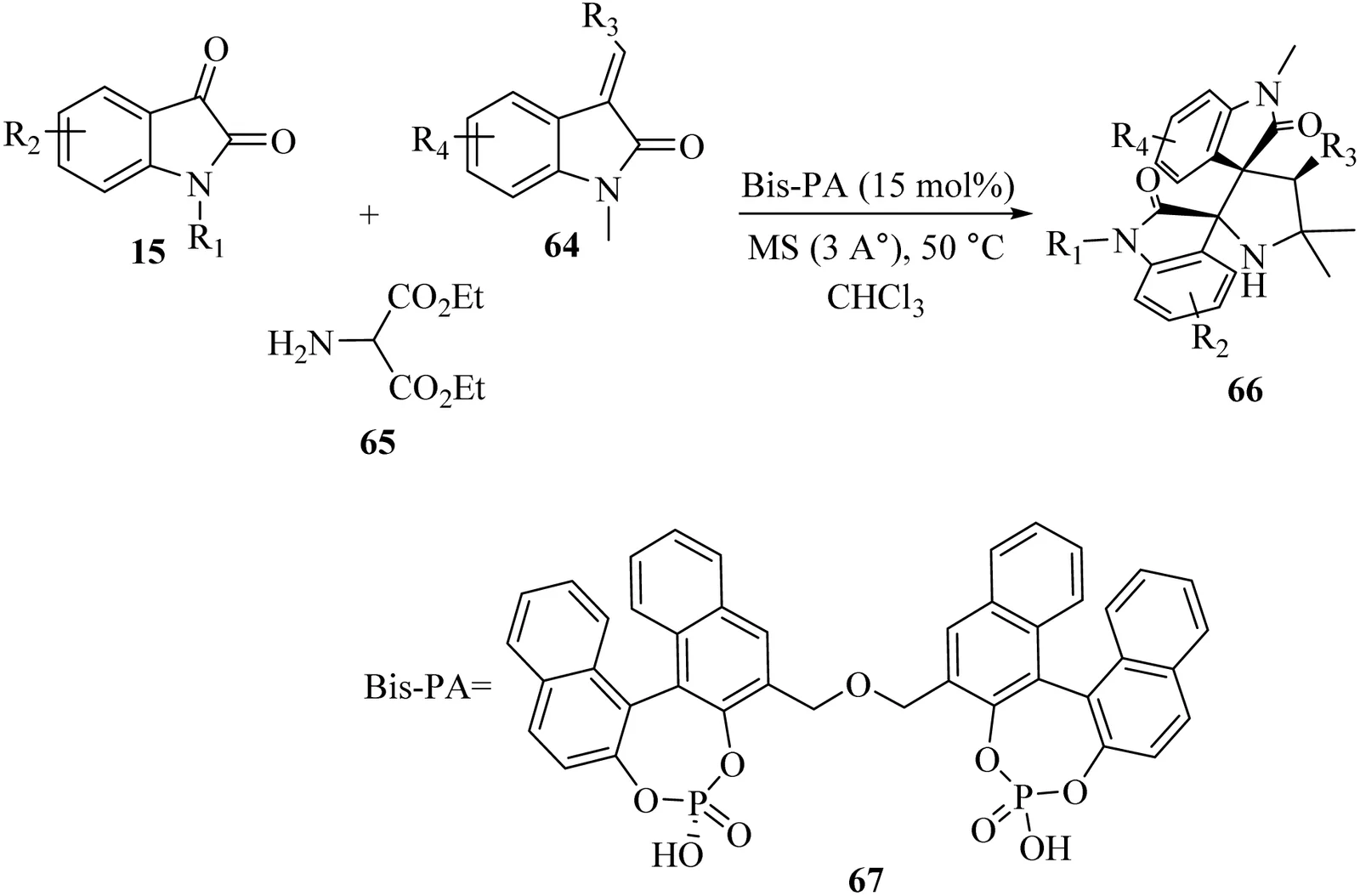
Catalytic enantioselective synthesis of a 3,3′-pyrrolidinyl bisspirooxindole.

Moreover, this reaction not only provides a powerful approach for the stereocontrolled construction of asymmetric bisspirooxindoles, but also significantly expands the application scope of asymmetric 1,3-dipolar cycloaddition reactions for the synthesis of biologically important spiro compounds with high optical purity.

In 2014, Kia *et al.* developed an efficient ionic liquid mediated, 1,3-dipolar cycloaddition methodology for the synthesis of highly functionalized mono- and bisspiro-pyrrolidines as potent cholinesterase inhibitors. The three-component [3+2]-cycloaddition reaction between a series of 69 and an azomethine ylide generated *in situ* from the condensation of isatin 6 and phenylglycine 68 was carried out in 1-butyl-3-methylimidazoliumbromide ([bmim]Br). [bmim]Br afforded the best overall performance, significantly improving both the reaction rate and isolated yield while providing a readily recyclable and environmentally benign reaction medium. The synthesized compounds were evaluated *in vitro* for their inhibitory activities against AChE and BChE. Most compounds exhibited significant AChE inhibitory activity, with IC_50_ values ranging from 1.68 to 21.85 µM. Molecular modeling studies further provided valuable insights into the observed inhibitory activities and were in good agreement with the experimental results (Scheme 15).^67^

**Scheme 15:**
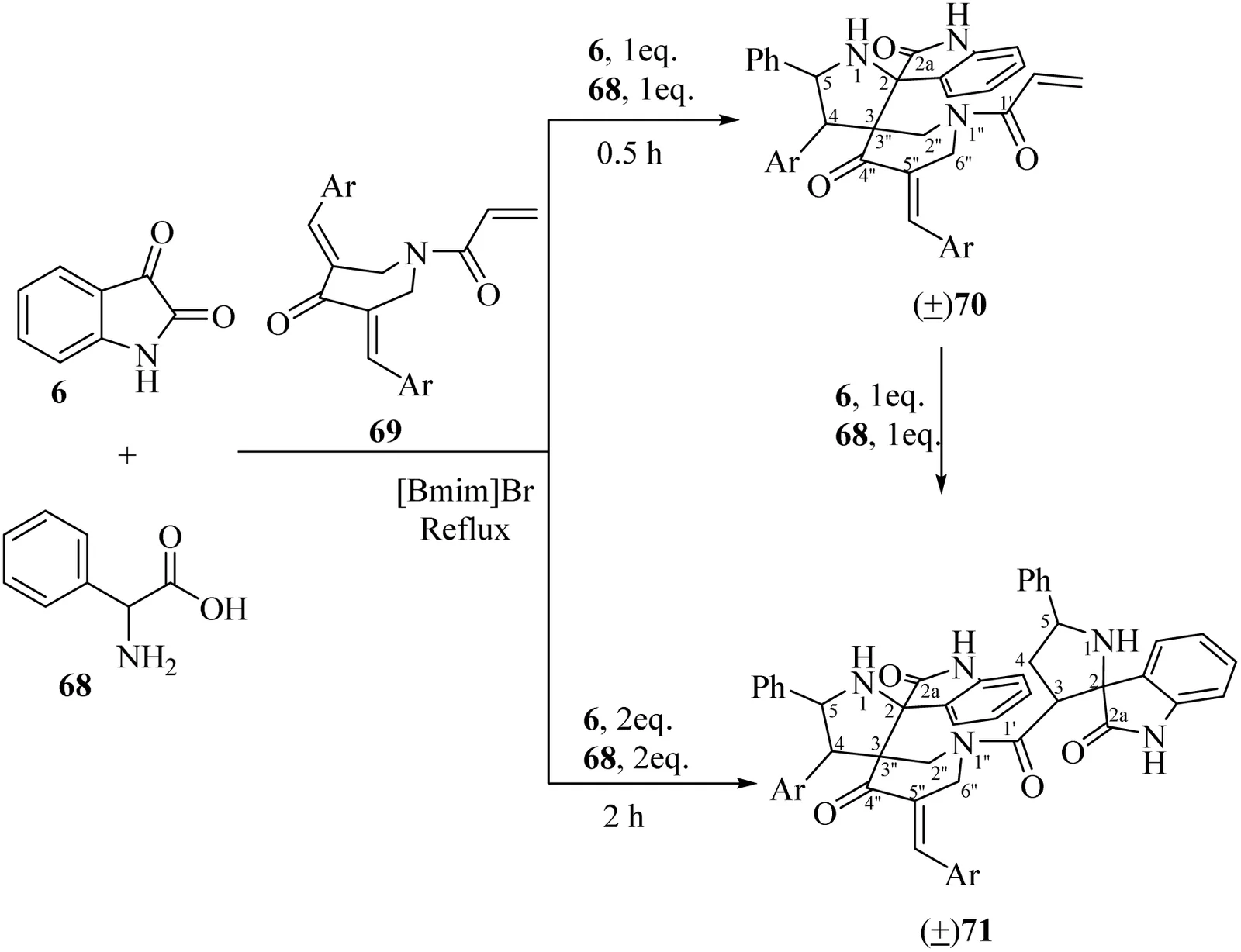
Construction of highly functionalized mono- and bisspiro-pyrrolidines.

In the same year, Lanka and colleagues proposed a chemo- and regioselective approach for the synthesis of bisspirooxindole and trisspirooxindole derivatives by controlling the molar ratio of reactants in a [3+2] cycloaddition reaction between azomethine ylides and dipolarophiles. The dipolarophile, which was assembled *via* a two-step process using various isatins 50 and acetylenic coumarin 72, contains both endo- and exocyclic double bonds, making it highly reactive in cycloaddition reactions. In their initial investigation, they conducted a three-component, sarcosine, and the dipolarophile in methanol under reflux conditions. After 2 hours, the reaction yielded highly substituted bisspirooxindole derivatives 74 as the exclusive products. Subsequently, by increasing the molar ratio of isatin and sarcosine to two equivalents, the reaction furnished trisspirooxindole 73 derivatives after 14 hours. This stoichiometry-controlled approach offers an efficient and selective route for the synthesis of complex spirooxindole architectures, which are highly valued in medicinal chemistry because of their presence in a wide range of bioactive compounds (Scheme 16).^68^ Moreover, the researchers evaluated l-proline as an alternative to sarcosine. In both experimental setups, using one or two equivalents of isatin and proline, the reaction exclusively produced bisspiropyrrolizidinyl oxindole derivatives 75, highlighting the decisive role of the amino acid structure in determining the final product.

**Scheme 16:**
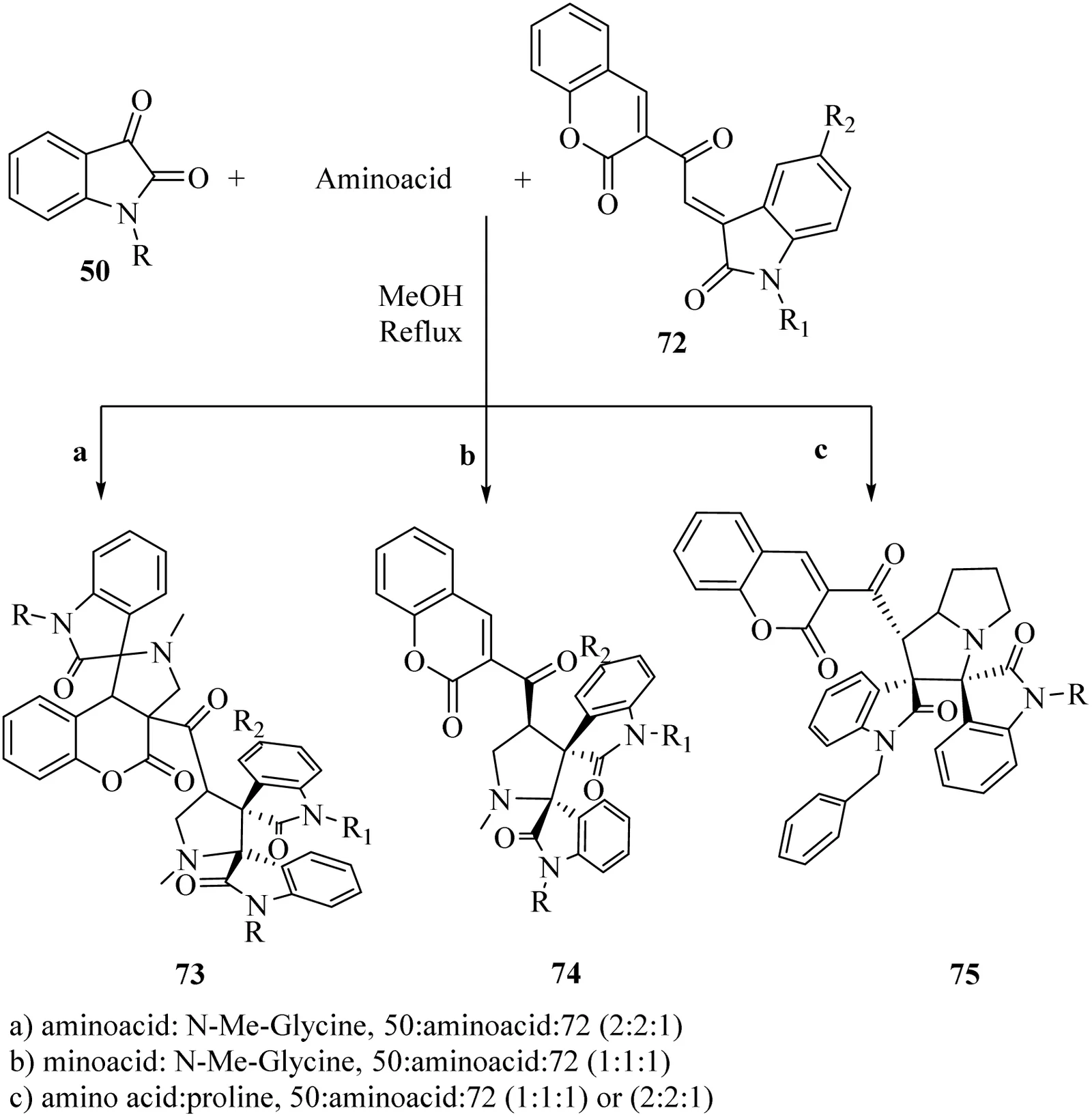
Construction of trisspiropyrrolidinyloxindole 73, bisspiropyrrolidinyloxindole 74, and bisspiropyrrolizidinyloxindole 75.

In 2014, Yang *et al.* designed an efficient, one-pot, and regioselective method for the synthesis of bisspiropyrrolizidine derivatives 77*via* a 1,3-dipolar cycloaddition reaction between azomethine ylides and (Z)-4-(4-nitrobenzylidene)-2-phenyloxazol-5(4H)-one 76 as the dipolarophile. Furthermore, the electronic nature of the substituents on the phenyl ring of the dipolarophile was found to markedly influence the reaction outcome. In particular, dipolarophiles bearing electron-withdrawing substituents, such as nitro (–NO_2_), fluoro, chloro, or bromo exhibited enhanced reactivity, delivering the corresponding products 77 in higher yields and shorter reaction times. In contrast, electron-donating substituents like methoxy or methyl resulted in lower yields and longer reaction times (Scheme 17).^69^

**Scheme 17:**
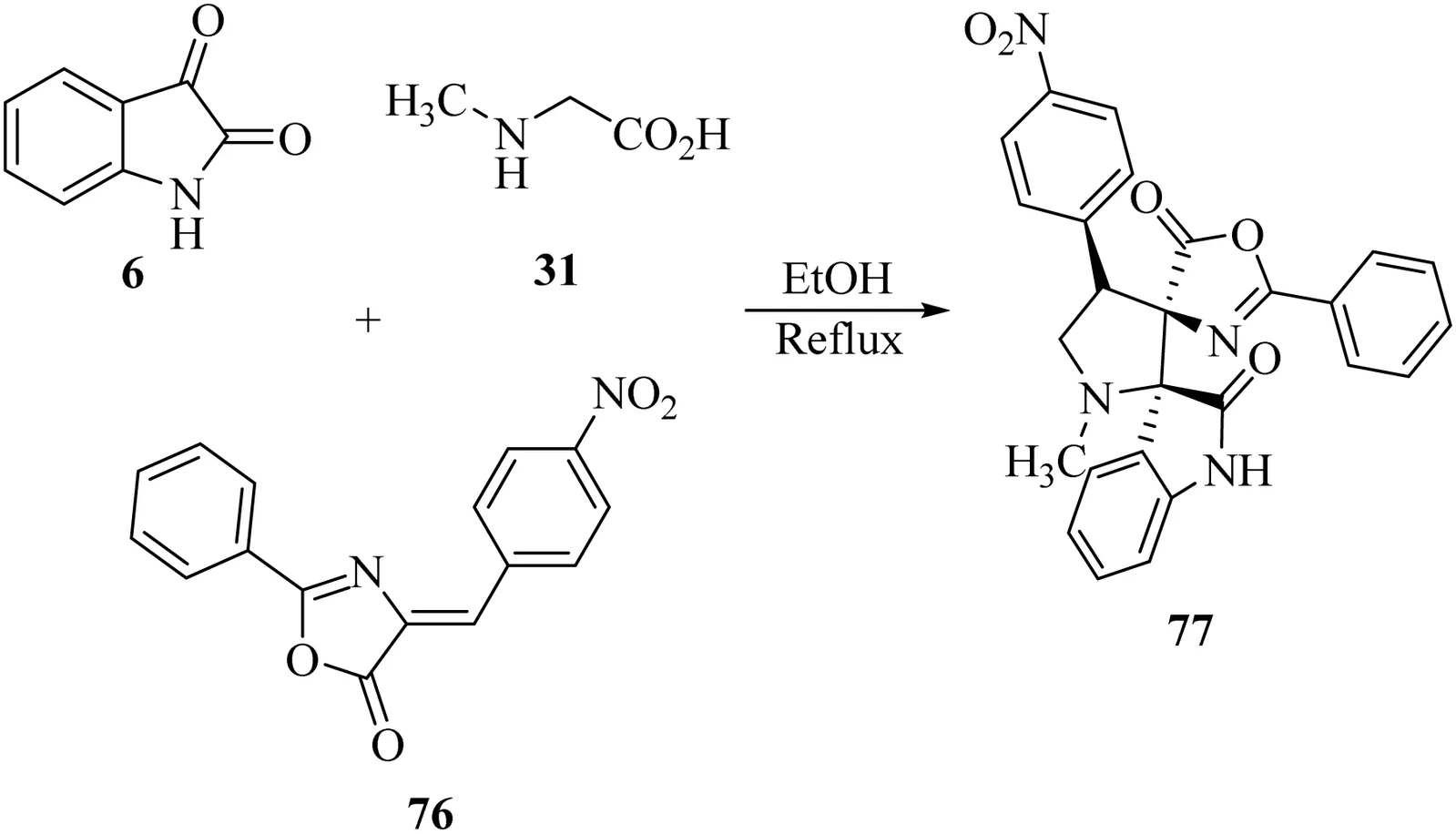
Synthesis of functionalized bisspiropyrrolizidine *via* a 1,3-dipolar cycloaddition reaction.

This method offers several advantages, including high atom economy, excellent yields, mild reaction conditions (using ethanol as the solvent under reflux condition), and high selectivity. The resulting bisspiropyrrolizidine compounds exhibit pharmaceutical potential, particularly antiproliferative activity, suggesting their promise as lead structures for anticancer drug development. Additionally, the reaction can be finely tuned to achieve chemo-, regio-, and stereoselectivity.

In 2013, Ramesh and coworkers developed a simple and effective method for the synthesis of bisspirooxindole-pyrrolidines 80 with good regio- and diastereoselectivity. This reaction is catalyzed by only 0.5 mol% of ceric ammonium nitrate (CAN) and is carried out in water, which makes the process environmentally friendly. The plausible mechanism for this reaction involves azomethine ylides functioning as dipoles that react with the dipolarophile, exocyclic enone derived from isatin, to form the desired products. All the obtained compounds showed good antimicrobial activity against both Gram-positive bacteria, such as *Bacillus subtilis*, *Staphylococcus aureus*, and *Micrococcus luteus* and Gram-negative bacteria like *Salmonella paratyphi*, *Pseudomonas aeruginosa*, and *Salmonella typhi* (Scheme 18).^70^

**Scheme 18:**
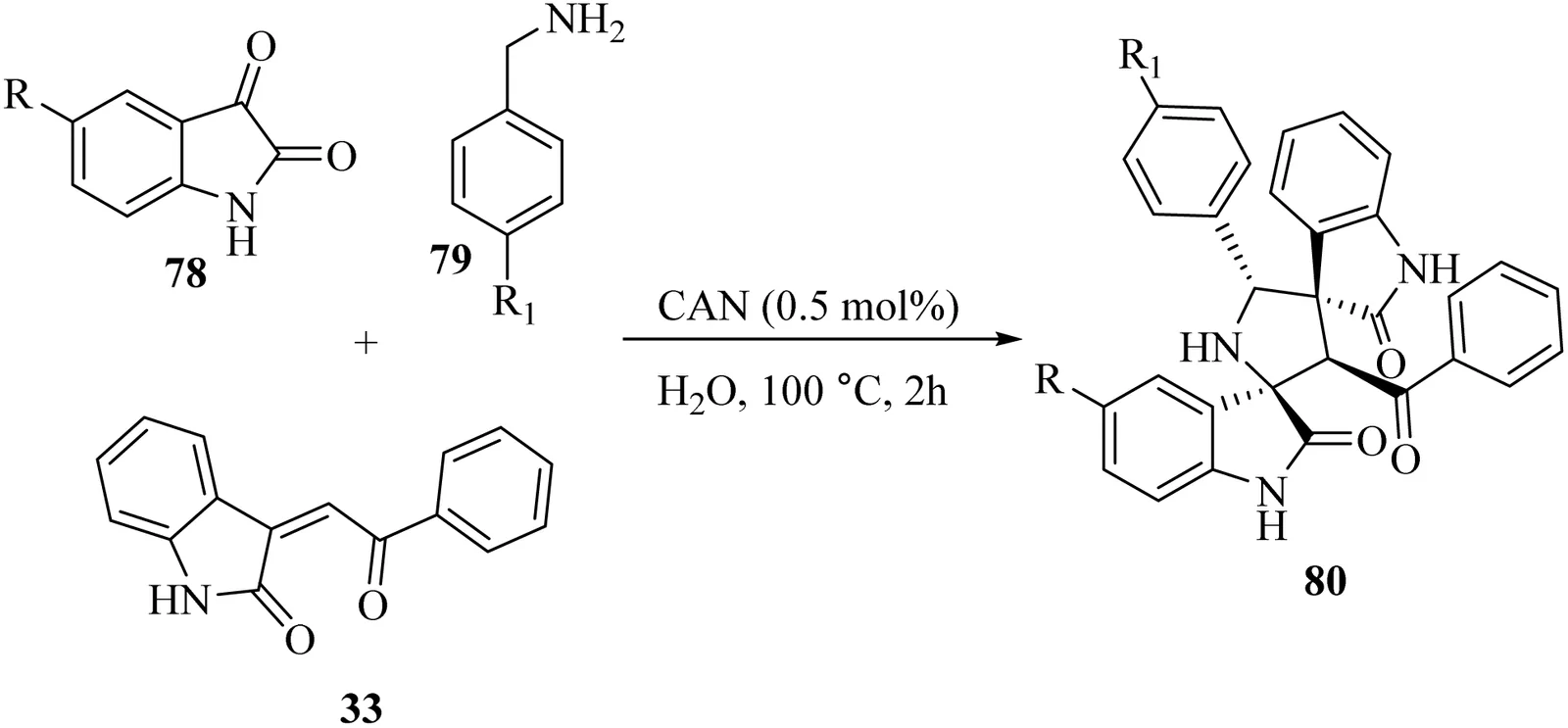
Synthesis of novel functionalized bisspirooxindoles as potential antimicrobial agents.

Synthesis of novel bisspirooxindoles, 3-acetyl-5-phenyl-pyrrolo(spiro-[2.3′]-1′-ben zyl-oxindole)-spiro-[4.3″]-1″-benzyl-oxindoles 84, 85*via* a three-component 1,3-dipolar cycloaddition reaction was developed by Chen Xiao's research group. In this strategy 1,3-dipolar cycloaddition of an azomethine ylide with 3-acetonylideneoxindole 81, yielded the desired products in high regioselectivity and good yields. An unusual regioselectivity was observed, presumably due to the presence of benzylamine 83, as the reaction conducted with proline 26 gave different results. This altered selectivity ultimately led to the synthesis of compounds featuring a novel structural scaffold (Scheme 19).^71^

**Scheme 19:**
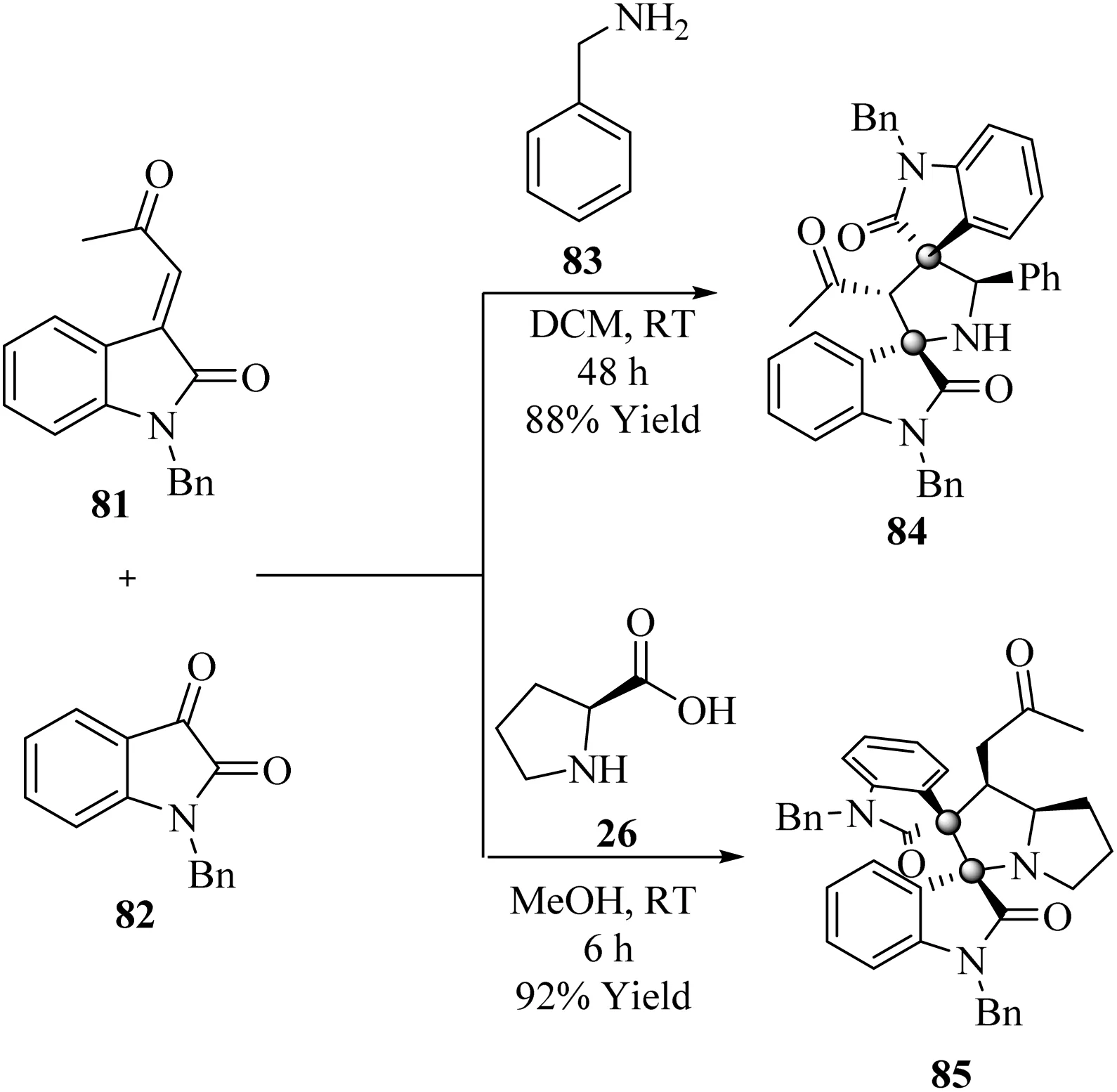
Synthetic route toward pyrrolo(spiro-[2.3′]-oxindole)-spiro-[4.3″]-oxindole.

A regio- and stereoselective synthesis of novel bisspiropyrrolidine bisoxindole containing two different indole rings 87 was reported by Liu *et al.* in 2010. In this strategy, 1,3-dipolar cycloaddition reaction between 2-(2-oxoindolin-3-ylidene)-malononitrile and azomethine ylides generated *in situ* from isatine 15 and sarcosine 31, produced the desired products through a one-pot multicomponent procedure. Although both endo 88 and exo 89 transition-state models are conceivable for this transformation, the X-ray crystal structure of the product clearly supports an endo transition-state pathway. Accordingly, the proposed model provides a reasonable explanation for the observed regioselectivity and stereoselectivity (Scheme 20).^72^

**Scheme 20:**
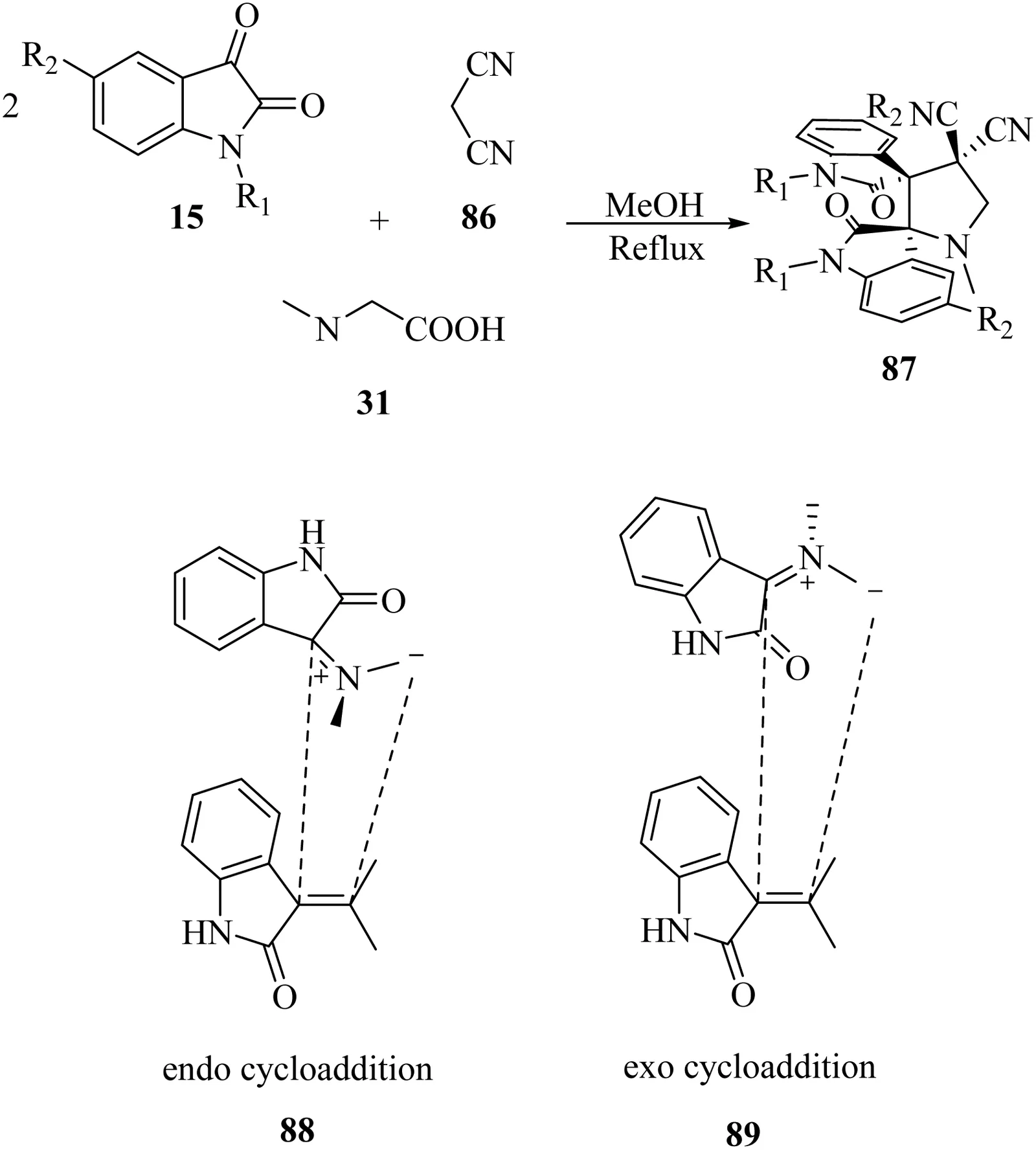
Regioselective synthesis of bisspiropyrrolidine bisoxindoles *via* multicomponent reactions.

Synthesis and antimycobacterial evaluation of bisspiro-oxindolylpyrrolothiazoles 91 was developed by Maheswari's research group in 2010. In this method, 1,3-dipolar cycloaddition of azomethine ylide, generated *in situ* from the reaction of 1,3-thiazolane-4-carboxylic acid 29 and isatin 78, to 2-arylidene-1,3-indanediones 90 afforded desired novel products with high regio- and stereoselectively in moderate to good yields (Scheme 21).^73^*In vitro* antitubercular screening of the synthesized compounds against *Mycobacterium tuberculosis* H37Rv identified spiro[5.3.0] 5′-nitrooxindole–spiro-[6.3.0]-1*H*-indene-1′,3(2*H*)-dione-7-(4-bromophenyl)tetrahydro-1*H*-pyrrolo[1,2-c][1,3]thiazole as the most potent compound, exhibiting an MIC of 1.4 µM and showing 3.4- and 5.4-fold greater potency than ciprofloxacin and ethambutol, respectively.

**Scheme 21:**
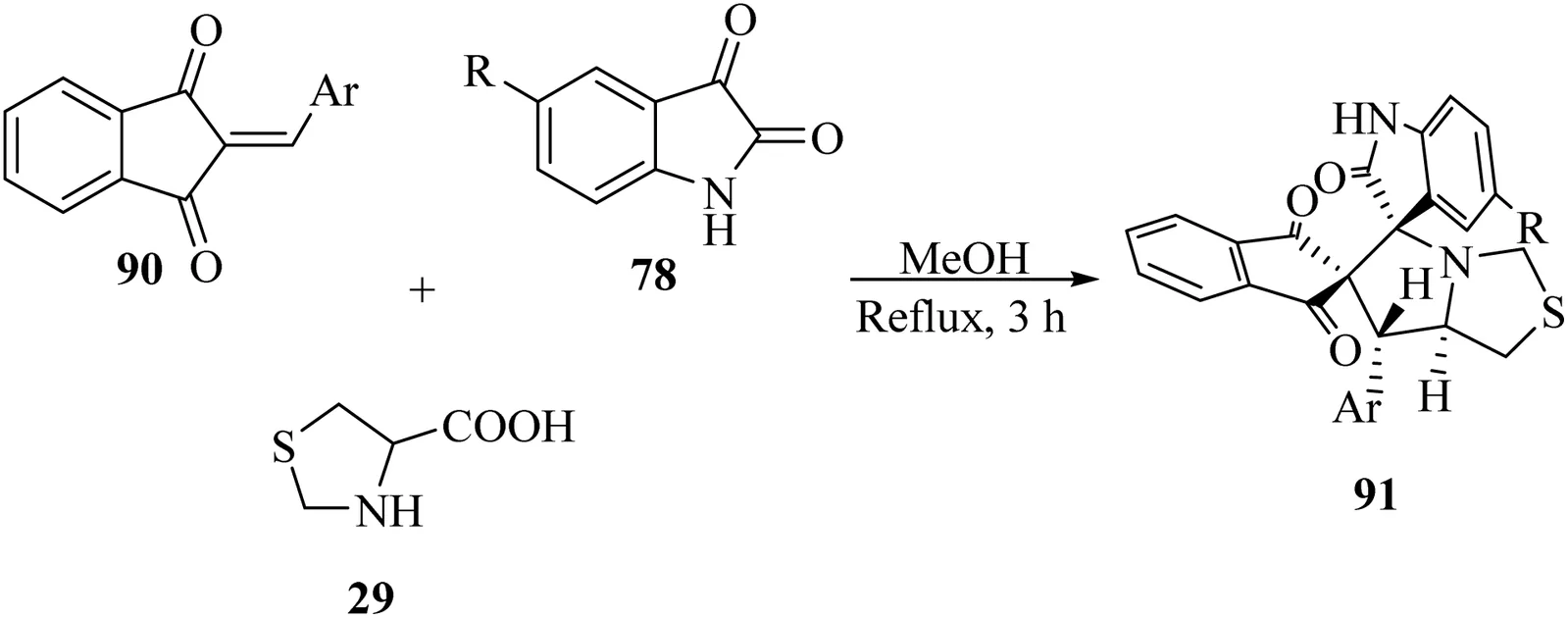
A facile synthesis of bisspirooxindolylpyrrolothiazoles as antimycobacterial agent.

In 2009 Adel S. Girgis reported a regioselective synthesis of bisspiro[1H-indene-2,3′-pyrrolidine-2′,3″ [3H]indole]-1,2″(1″H)-diones 52*via* 1,3-dipolar cycloaddition reaction of 2-(arylmethylene)-2,3-dihydro-1*H*-inden-1-ones 51 with non-stabilized azomethine ylides, generated *in situ via* the decarboxylative condensation of isatins 50 and sarcosine 31 (Scheme 22). Biological screening demonstrated that the synthesized compounds possess promising antitumor activity, particularly against the SK-MEL-2 melanoma cell line. Furthermore, *in vivo* evaluation using the carrageenan-induced rat paw edema model revealed significant anti-inflammatory activity, in some cases showing efficacy comparable to that of the reference drug indomethacin.^74^

**Scheme 22:**
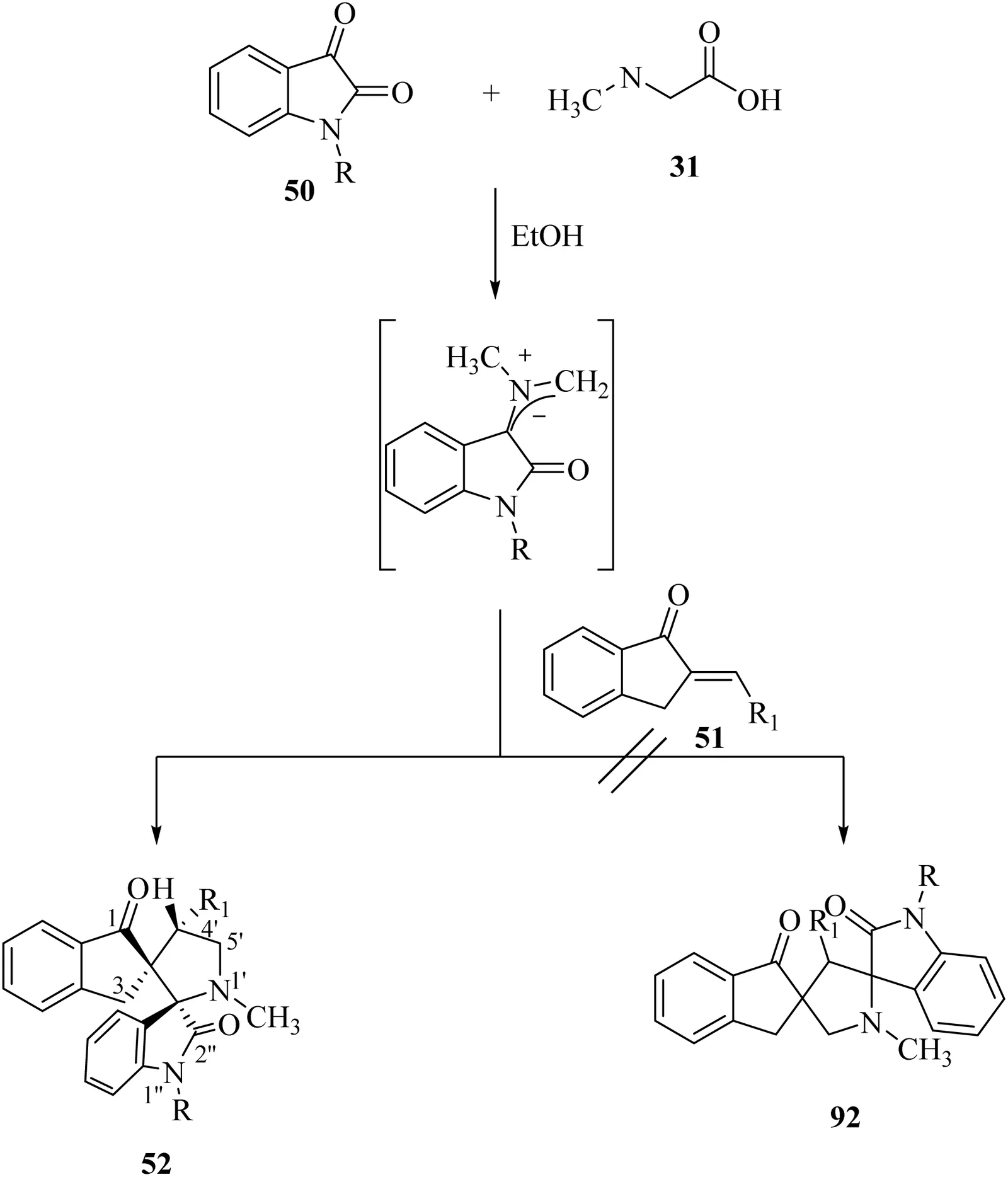
Regioselective synthesis of bisspiro[1H-indene-2,3′-pyrrolidine-2′,3″ [3H]indole]-1,2″(1″H)-diones for anti-tumor applications.

In 2008, Kumar and co-workers reported the stereoselective synthesis of a new class of spiro-piperidin-4-ones 94–96 through a 1,3-dipolar cycloaddition reaction involving *in situ*-generated azomethine ylides from isatin 6 and R-amino acids like proline 26, phenylglycine 68, and sarcosine 31 with a series of 1-methyl-3,5-bis[(E)-arylmethylidene]tetrahydro-4(1H)-pyridinones 93 (Scheme 23).^75^ Notably, both *in vitro* and *in vivo* biological studies demonstrated that the synthesized compounds displayed potent antimycobacterial activity against *Mycobacterium tuberculosis* H37Rv (MTB), multidrug-resistant *Mycobacterium tuberculosis* (MDR-TB), and *Mycobacterium smegmatis* MC2. These promising biological results identify this molecular scaffold as an attractive platform for the design and development of next-generation antitubercular agents.

**Scheme 23:**
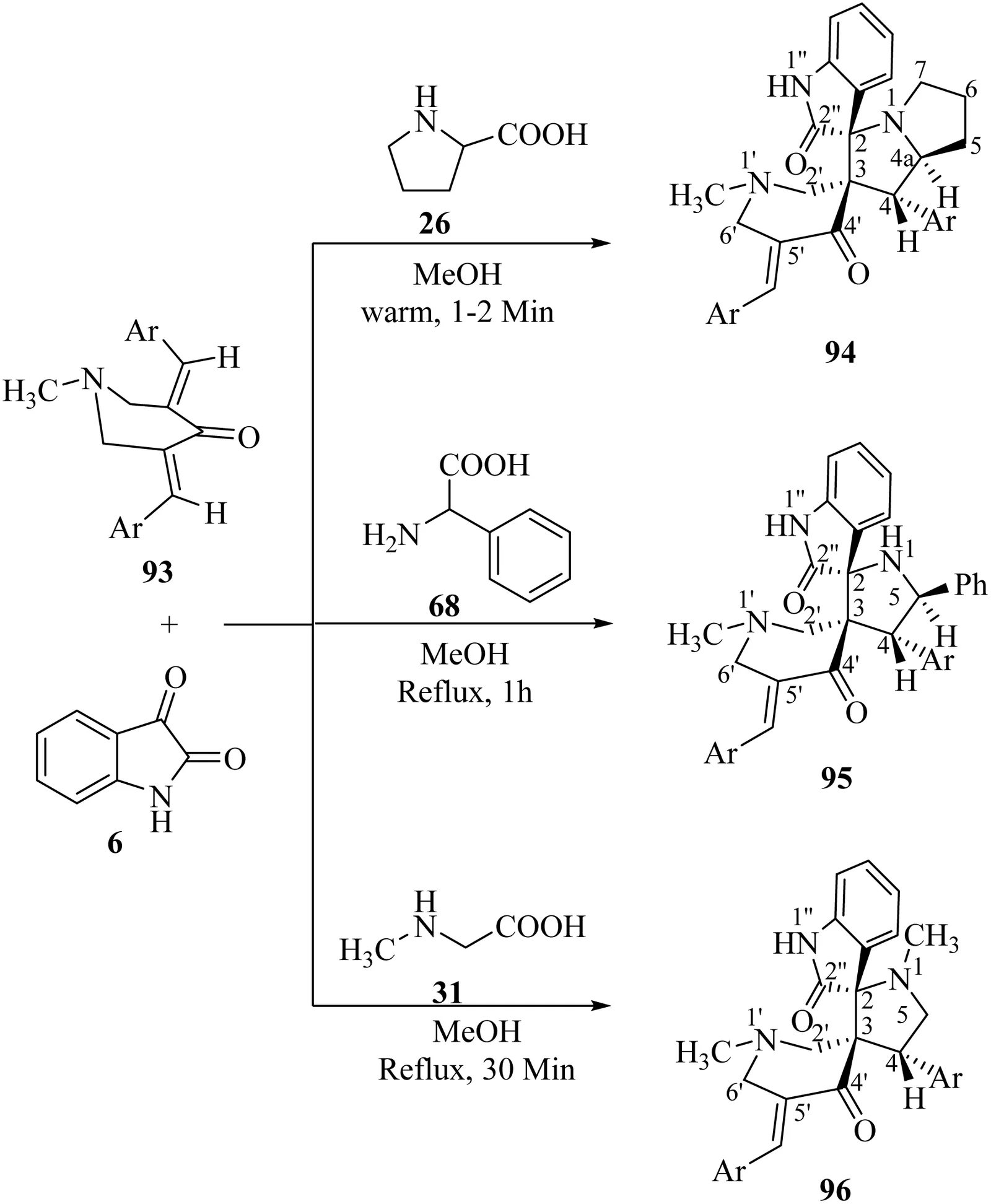
Stereoselective synthesis of spiro-piperidin-4-ones exhibit antimycobacterial activity.

## 3-Alkenyl oxindoles annulation toward bisspirooxindole

Among the various approaches reported for bisspirooxindole synthesis, methodologies employing 3-alkenyl oxindoles as key building blocks have emerged as particularly attractive strategies. These reactions typically undergo cyclization to furnish the bisspirooxindole core. The versatility of this approach has led to the development of numerous catalytic protocols, including organocatalyst, *N*-heterocyclic carbene (NHC) catalyst, squaramide-catalyst, transition-metal catalyst, as well as catalyst-free protocols, allowing the synthesis of a wide range of functionalized bisspirooxindoles under diverse reaction conditions. In this section, representative examples of bisspirooxindole syntheses based on 3-alkenyl oxindoles are discussed.

Very recently Chen *et al.* reported an organocatalytic cascade reaction of isatylidene malononitriles 97 and 2-ethylidene-1,3-indandiones 98 using quinine 100 as the catalyst for the synthesis of spiro-bridged heterocyclic compounds 99 (Scheme 24).^76^ The reaction proceeded *via* a chemoselective vinylogous Michael/cyclization/rearrangement sequence. An unexpected vinylogous Michael addition at the β-position of isatylidene-malononitriles, followed by aldol cyclization, alkoxide 1,2-addition to the nitrile, and [1,3]-O-to N rearrangement, afforded unique amide, indanone, and oxindole containing spiro-bridged heterocyclic compounds in good to excellent yields with high diastereoselectivity. The DFT calculation results supported the preference for vinylogous Michael addition at the β-position of isatylidene malononitriles and provided a rational explanation for the observed selectivity.

**Scheme 24:**
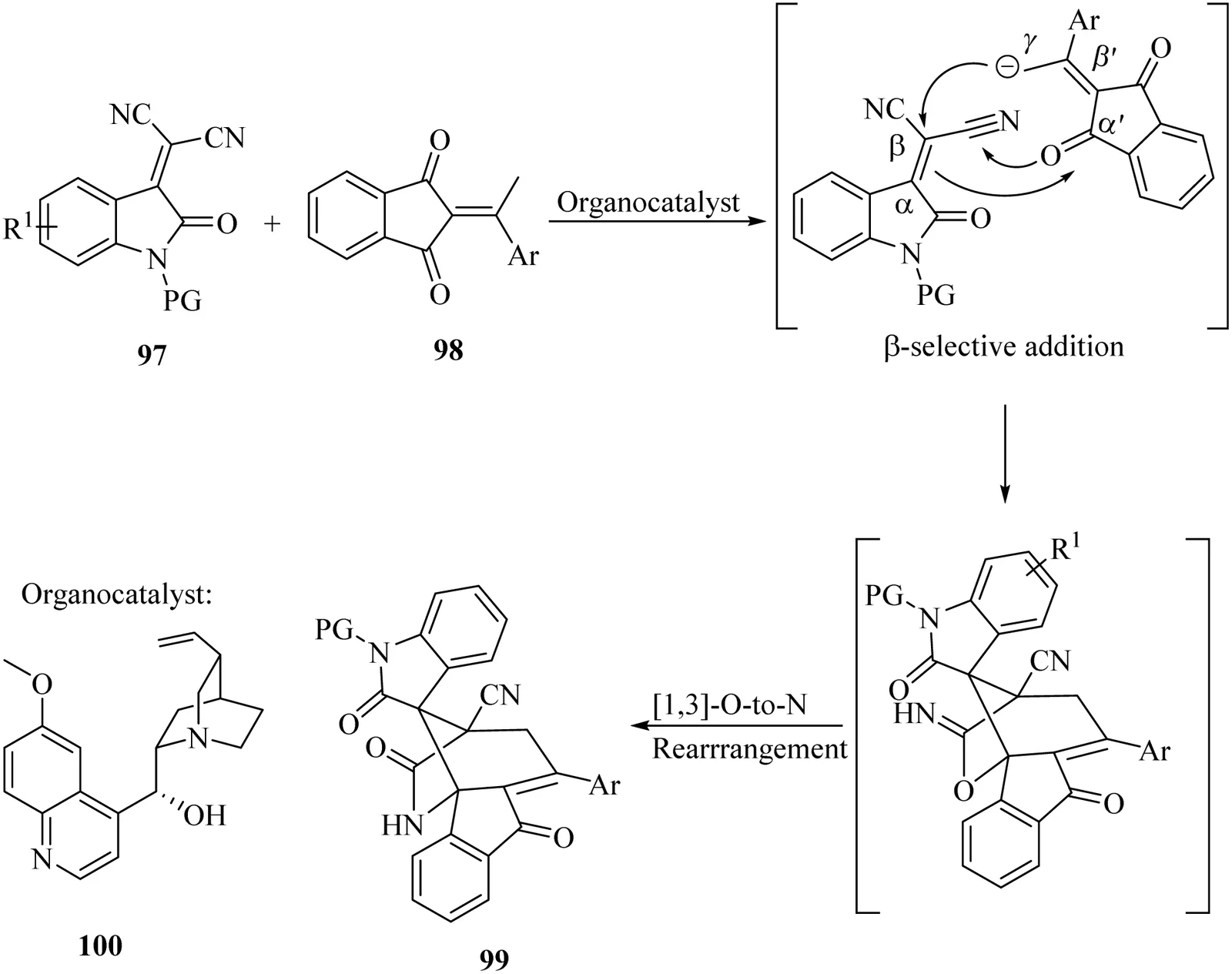
Organocatalytic synthesis of spiro-bridged heterocyclic compounds.

In 2024, Yu *et al.* developed an efficient asymmetric [2+1] annulation of 3-alkenyl oxindoles 101 with two classes of α-bromoketones 102, catalyzed by a chiral bifunctional phosphonium salt 104 (Scheme 25).^77^ Mechanistic investigations revealed that hydrogen-bonding interactions between the catalyst and the substrate are key determinants of stereocontrol. This methodology provides access to a wide range of multisubstituted spirocyclopropyl oxindoles and bisspiro[oxindole-cyclopropane-cyclohexanone] derivatives 103 with excellent enantioselectivities and high diastereoselectivities (up to 97% ee and 99 : 1 dr). The resulting bisspirocyclopropane frameworks represent promising structural motifs for the discovery and development of novel chiral drug candidates.

**Scheme 25:**
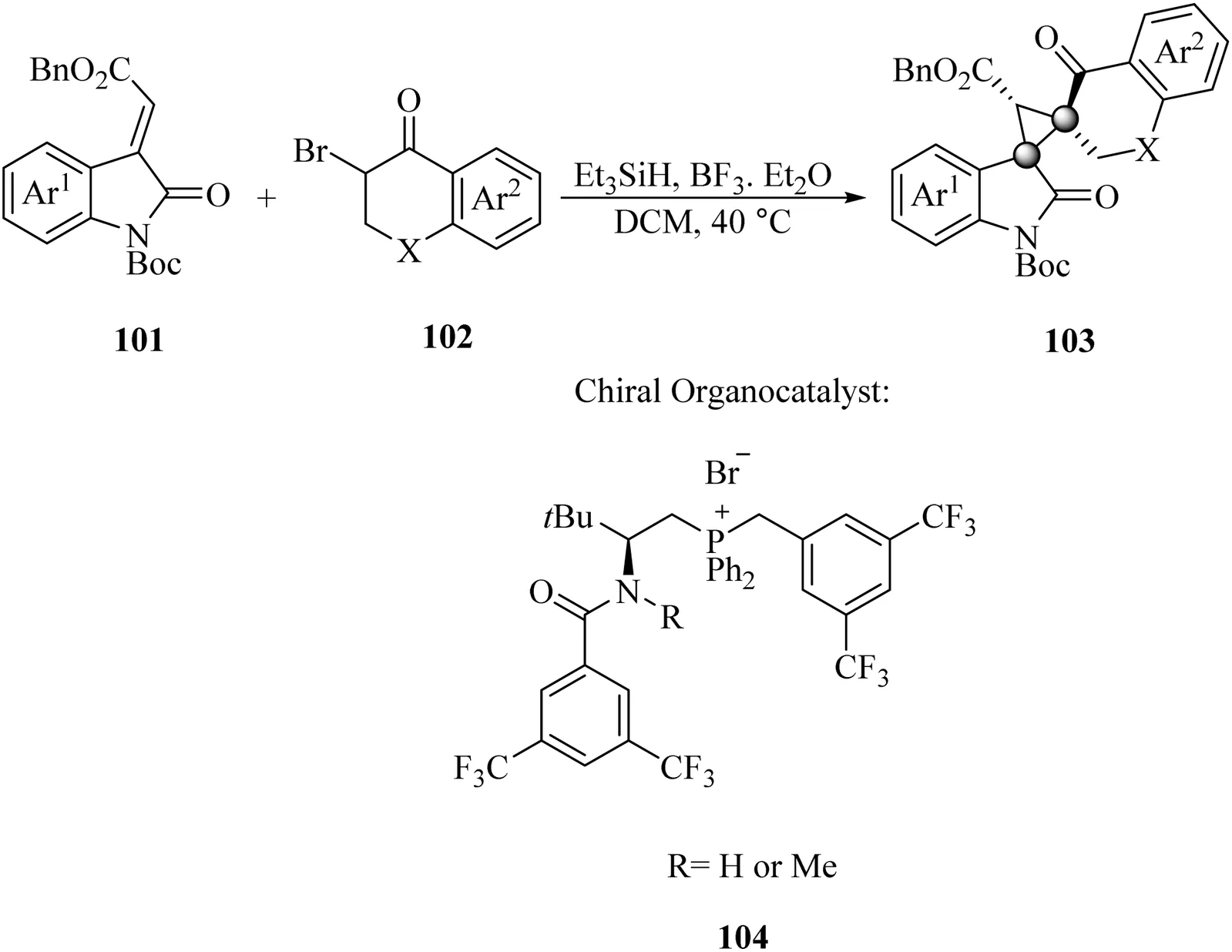
Asymmetric synthesis of bisspiro cyclopropane skeletons *via* bifunctional phosphonium salt-catalyzed [2+1] annulation.

In 2022, the Liu group, introduced a catalytic synthetic strategy based on an *N*-heterocyclic carbene (NHC)-catalyzed [3+2] annulation between bisoxindoles 105 and cinnamaldehyde 106 or β-bromoenal 108, providing access to products bearing two stereogenic centers with high levels of enantio- and diastereoselectivity (Scheme 26). Given that the construction of quaternary stereogenic centers remains a considerable challenge due to the limited efficiency and selectivity of conventional methods, this one-pot NHC catalytic strategy represents an efficient and highly enantioselective approach for the synthesis of such structurally complex motifs. The NHC generates a nucleophilic acyl-anion equivalent from α-bromoenals, which then undergoes a stereocontrolled cycloaddition with bisoxindoles. This mechanistic design allows the rapid formation of a spirocyclic core in a single step. The reaction afforded spirocyclic bisoxindoles in good to excellent yields and with remarkable stereocontrol. It also tolerated a broad range of α-bromoenals and bisoxindoles, indicating a wide substrate scope. Notably, this methodology enables the simultaneous construction of three stereogenic centers, including two contiguous all-carbon quaternary ones, in a single step, a transformation that is rarely achievable using conventional synthetic strategies.^78^

**Scheme 26:**
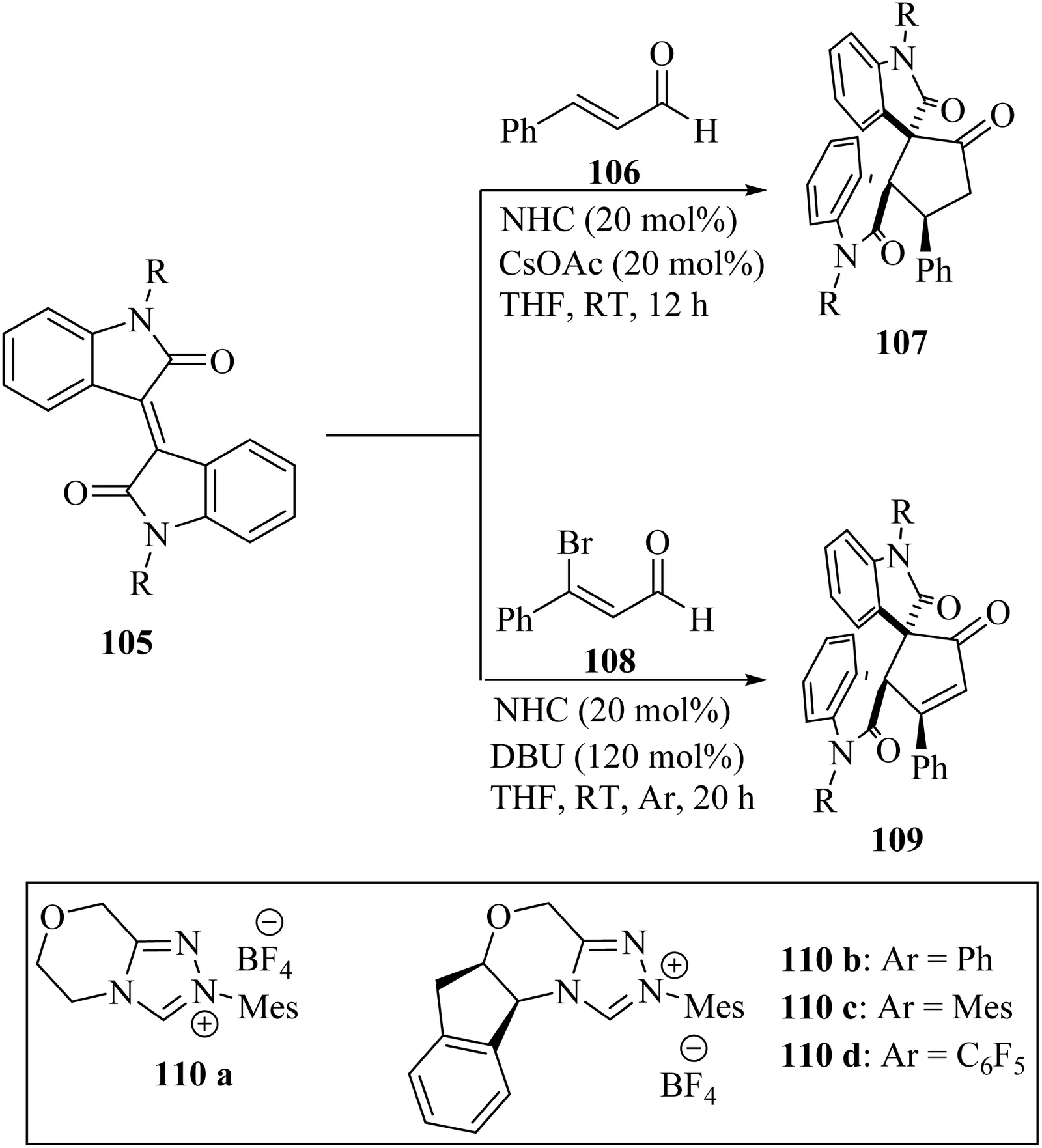
The NHC-catalyzed [3+2] annulation of isoindigos with enals.

Also, in the same year, The Lu group reported an organocatalytic asymmetric [3+2] annulation reaction between 3-methyleneoxindoles 111 and ninhydrin-derived Morita–Baylis–Hillman (MBH) carbonates 112. This method provided a highly efficient and stereoselective approach to the construction of a a range of structurally diverse bisspiro compounds 113–114, mainly those containing spiroindane-1,3-dione and spirooxindole frameworks. The reaction exhibited both β- and γ-selectivity, achieving moderate to excellent yields under mild reaction conditions (Scheme 27).^79^

**Scheme 27:**
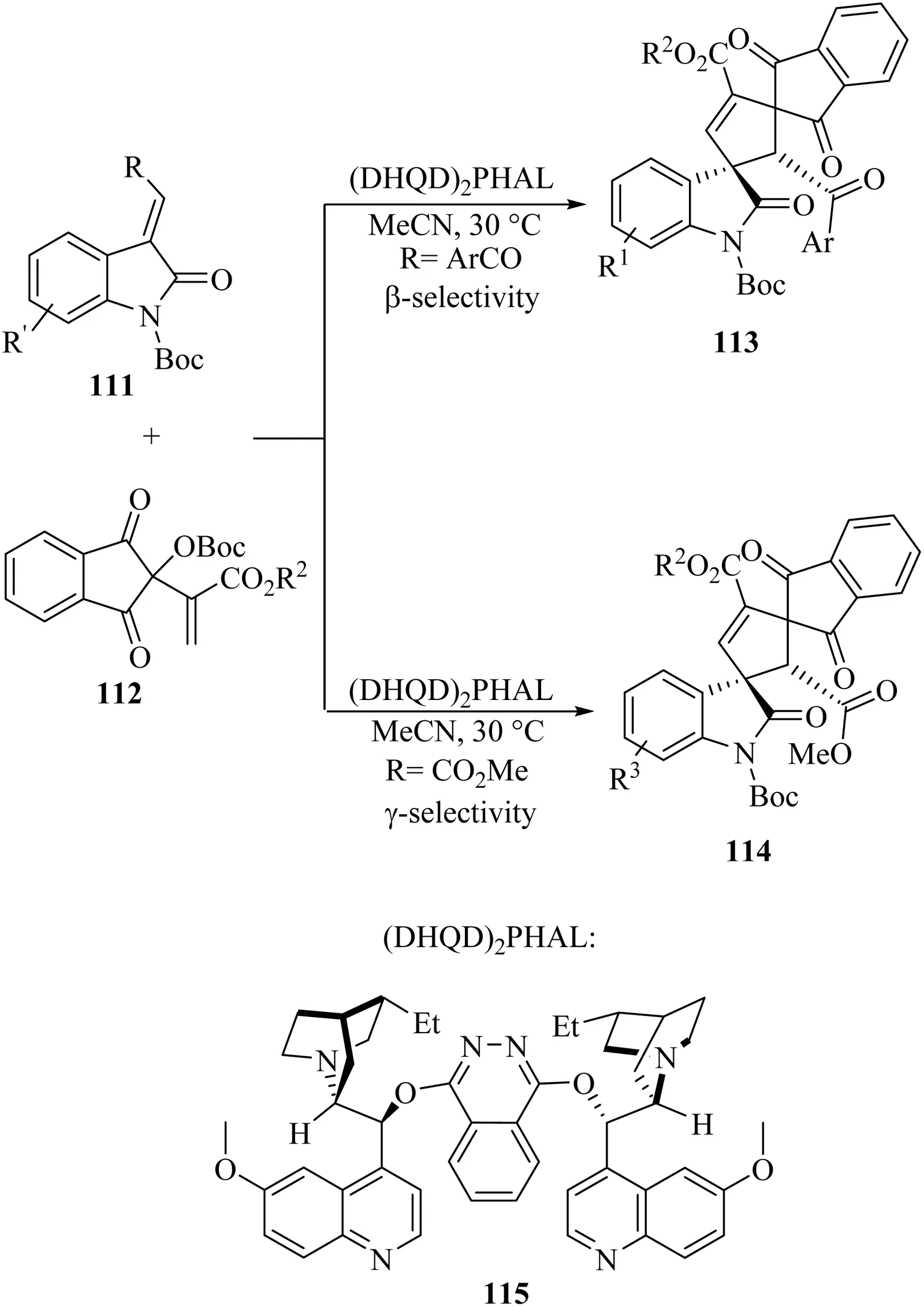
Organocatalytic regio-and enantioselective approach to bisspirooxindole framworks.

Li's research broadened the synthetic applications of MBH carbonates, offering a practical route to complex chiral spirocyclic architectures with potential applications in organic and medicinal chemistry.

In 2021, Jia *et al.* developed a palladium(0)-catalyzed asymmetric (4+2) annulation between γ-methylidene-δ-valerolactones (GMDVs) 116 and para-quinone methides (QMs) 117. The reaction proceeded smoothly under mild conditions, affording cyclohexadienone-fused cyclohexyl spirooxindoles 118 in high yields and with excellent stereoselectivity (up to 99% yield, dr > 20 : 1, 95% ee). This method simultaneously forms three contiguous tetrasubstituted carbon atoms, including two vicinal quaternary centers, providing access to complex chiral spiro frameworks. It represents the first example of an enantioselective (4+2) annulation using GMDVs 116 with *p*-QMs 117 (Scheme 28).^80^

**Scheme 28:**
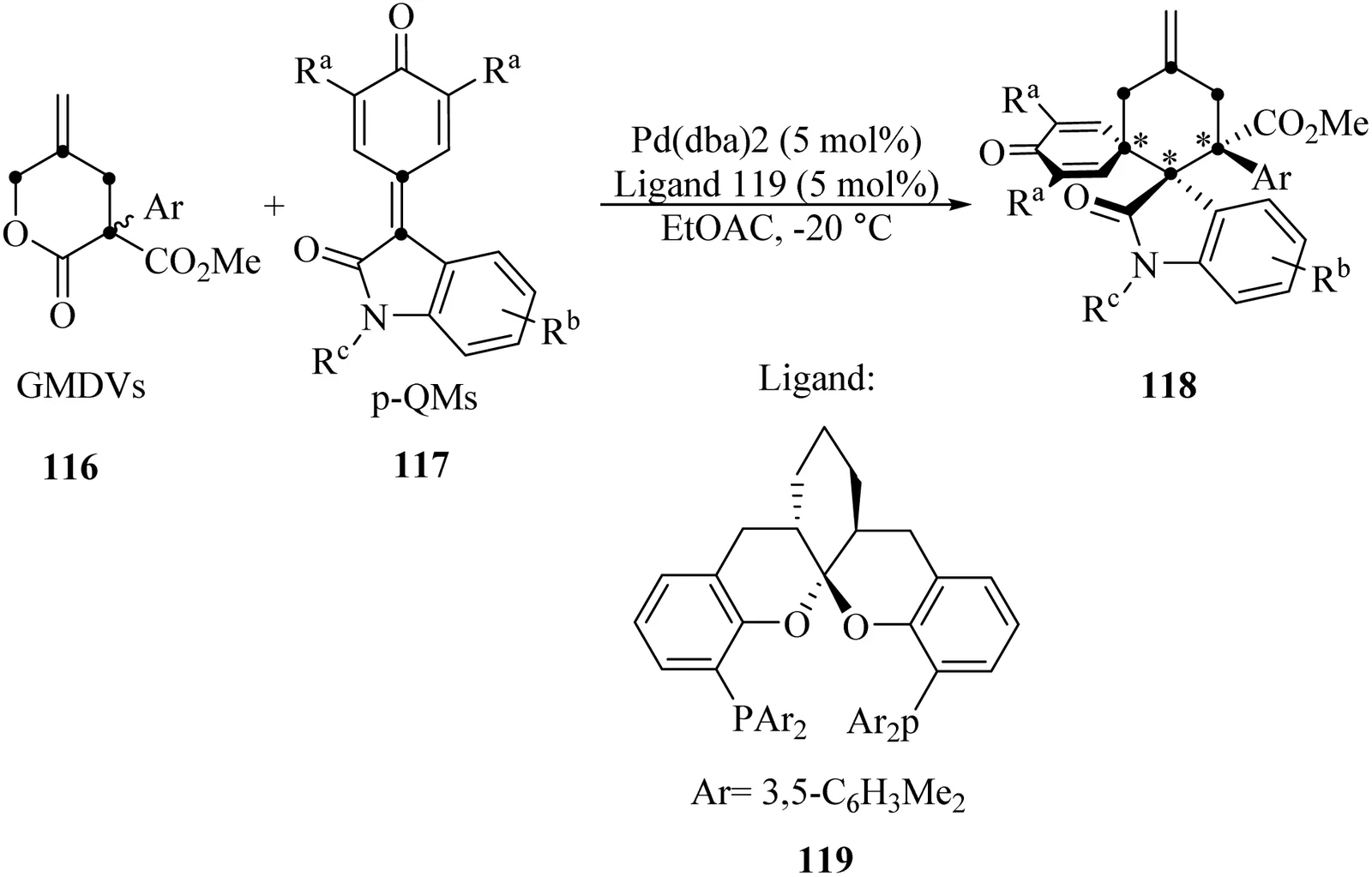
Palladium-catalyzed asymmetric synthesis of cyclohexadienone-fused cyclohexyl spirooxindoles.

Siva and co-workers (2021) reported a regio- and stereoselective cascade [3+2] annulation involving nitrosatylidene derivatives 120 as vinylogous Michael donors reacting with various electron-deficient alkenes 121 (Scheme 29). This transformation proceeded through two consecutive Michael additions, generating bisspiro-bisoxindoles bearing four contiguous chiral centers, including two quaternary spiro carbons. Notably, the method afforded moderate to excellent yields and high diastereoselectivity, providing a practical route to rigid γ-amino acid derivatives that serve as valuable building blocks in asymmetric synthesis.^81^

**Scheme 29:**
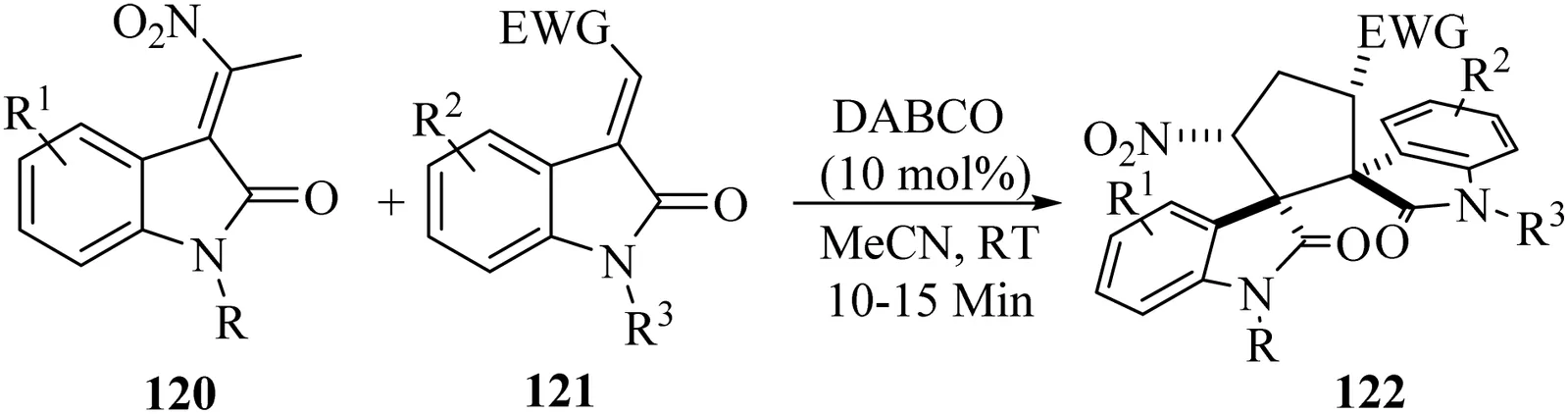
Regio- and stereoselective synthesis of bisspiro-bisoxindoles *via* [3+2] annulation reaction.

In 2020, Luo *et al.* described a highly regio-, diastereo-, and enantioselective [3+2] annulation between γ-substituted allenoates 123 and pyrazolonyldiene oxindoles 124 (Scheme 30). In this reaction, a ferrocene-derived bifunctional phosphine catalyst 126 was employed, enabling cooperative activation through metal–amine interaction. The reaction afforded the desired spiro-cyclopentene-linked oxindole and pyrazolone frameworks 125 in good to excellent yields (up to 95%), bearing three contiguous stereogenic centers, including two vicinal spiro-quaternary carbons. Notably, this study presents the first efficient strategy for the construction of spirooxindole-fused spiropyrazolones bearing consecutive stereogenic centers, providing access to structurally complex chiral spiro scaffolds with potential applications in the design of bioactive molecules.^82^

**Scheme 30:**
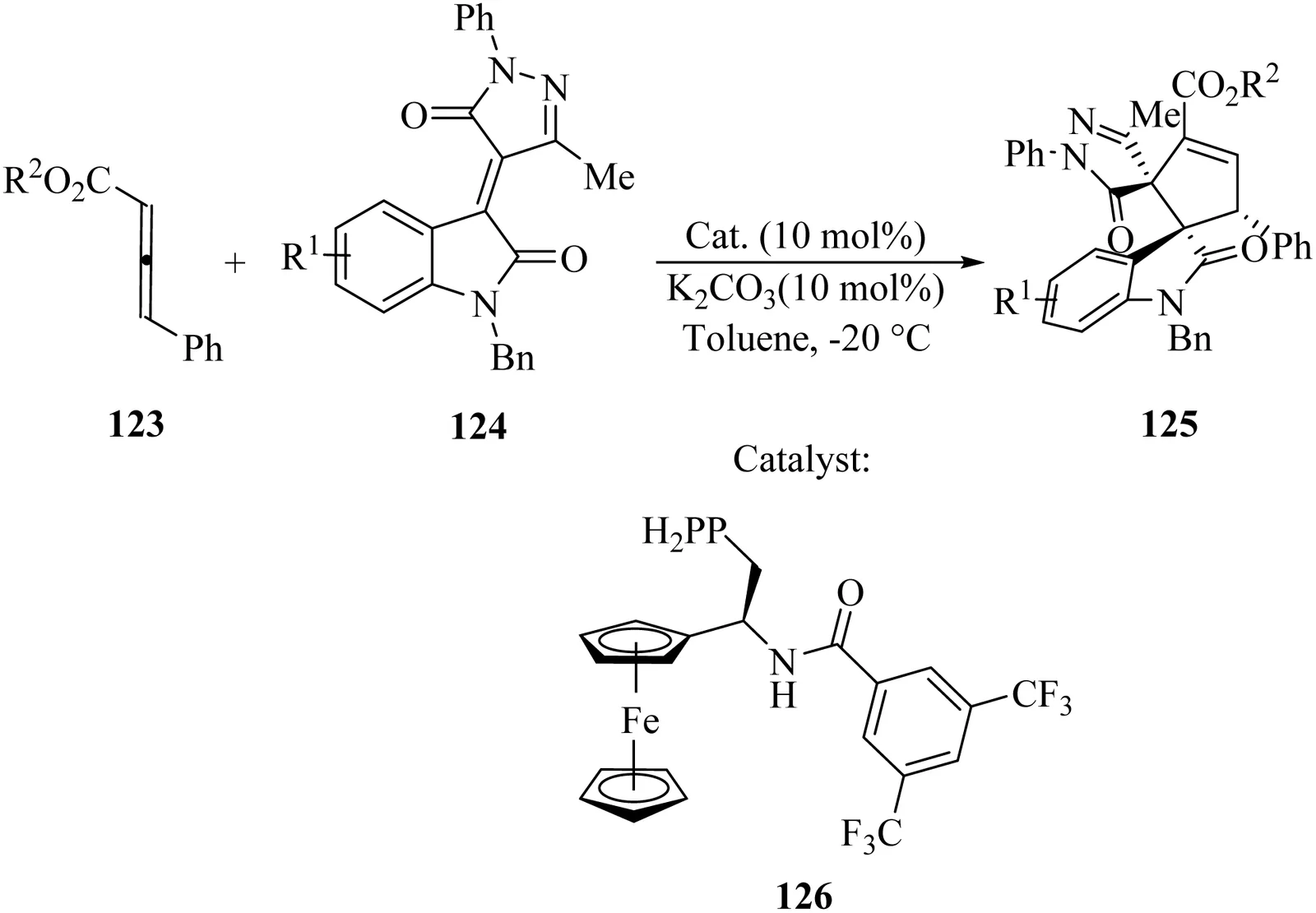
Construction of spirooxindole-fused spiropyrazolones possessing contiguous three stereogenic centres.

In 2020, Chen *et al.* described an efficient organocatalytic approach for the asymmetric construction of bisspiro[2,1′-indoline-3′,3-dihydrofuran-3-carbonitrile] bisoxndoles 130 bearing adjacent spiro stereocenters. Their study initially explored a conventional cascade aldol–cyclization–tautomerization pathway (Scheme 31, Path A); however, despite optimization of the catalyst and reaction conditions, this route afforded only moderate enantioselectivity, indicating intrinsic limitations in stereochemical control. To overcome this drawback, the authors redesigned the reaction by implementing a novel Umpolung strategy that, altered the polarity of the reacting partners and enabled an alternative cascade pathway (Scheme 31, Path B). Under this revised mechanism, a cinchona-derived thiourea organocatalyst 131 promoted efficient dual activation, leading to the desired bisspiro frameworks in moderate yields (∼46%), but with excellent diastereo- and enantioselectivity (up to 99 : 1 dr and 95% ee). This work represents the first umpolung-based cascade reaction for the enantioselective synthesis of complex bisspiro architectures and highlights the impact of pathway redesign on stereocontrol in spirocyclic molecule construction.^83^

**Scheme 31:**
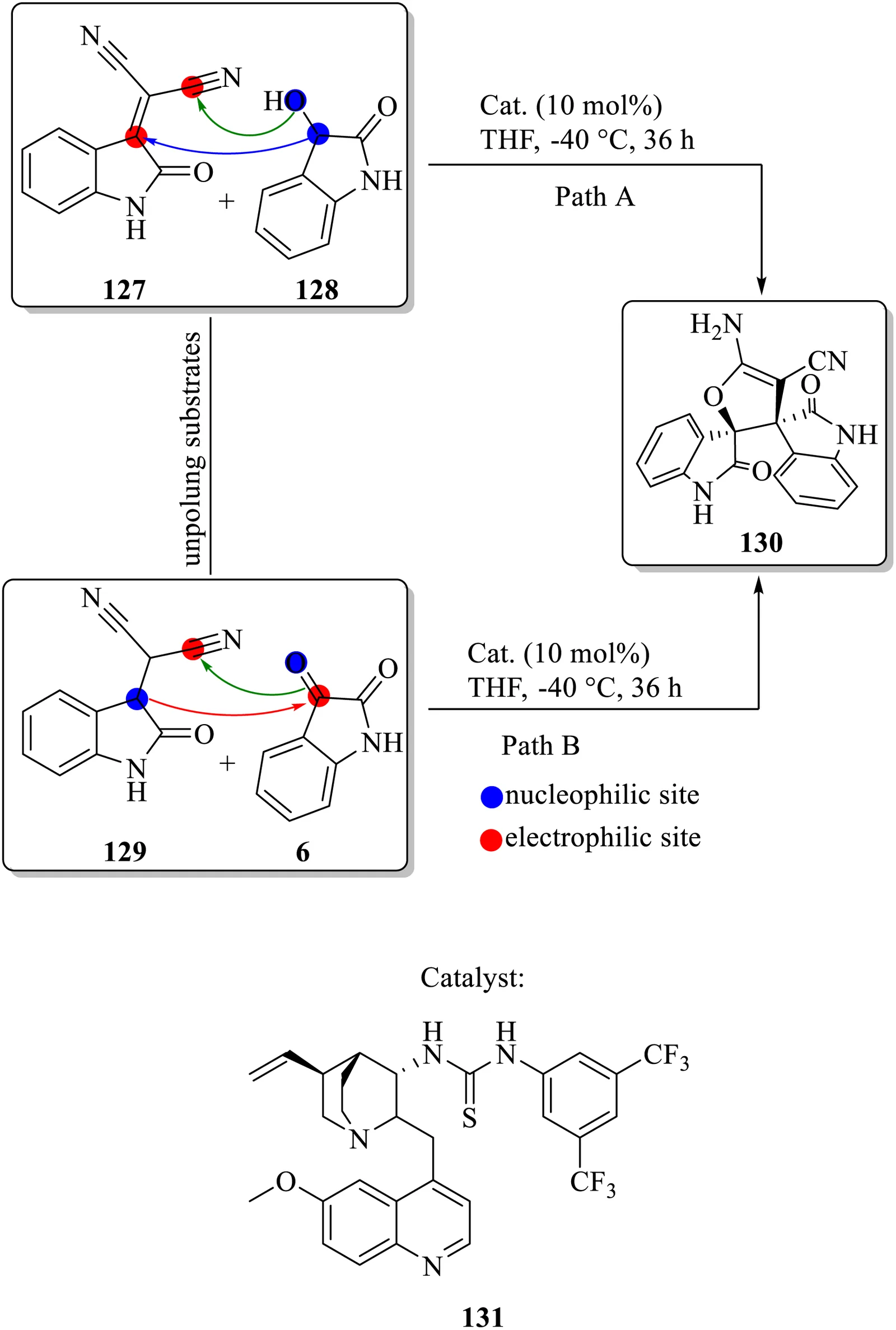
Umpolung strategy for the construction of chiral bisspiro[2-amino-4,5 dihydrofuran-3-carbonitrile]bisoxindoles.

Suman and colleagues introduced a Lewis acid-catalyzed domino [3+2] cycloaddition strategy for the efficient construction of 3,3′-pyrrolidinyl-bisspirooxindoles 134 containing four contiguous chiral stereogenic centers and two neighboring quaternary spirostereocenters (Scheme 32). The reaction proceeds *via* an azide–alkene cycloaddition cascade involving rearrangement and aziridine ring opening, affording highly regio- and stereoselective products bearing dense functionalization. This one-step transformation enables the simultaneous formation of two new C–C bonds in a single operation, providing a single, highly functionalized chiral isomer in good yields.^84^

**Scheme 32:**
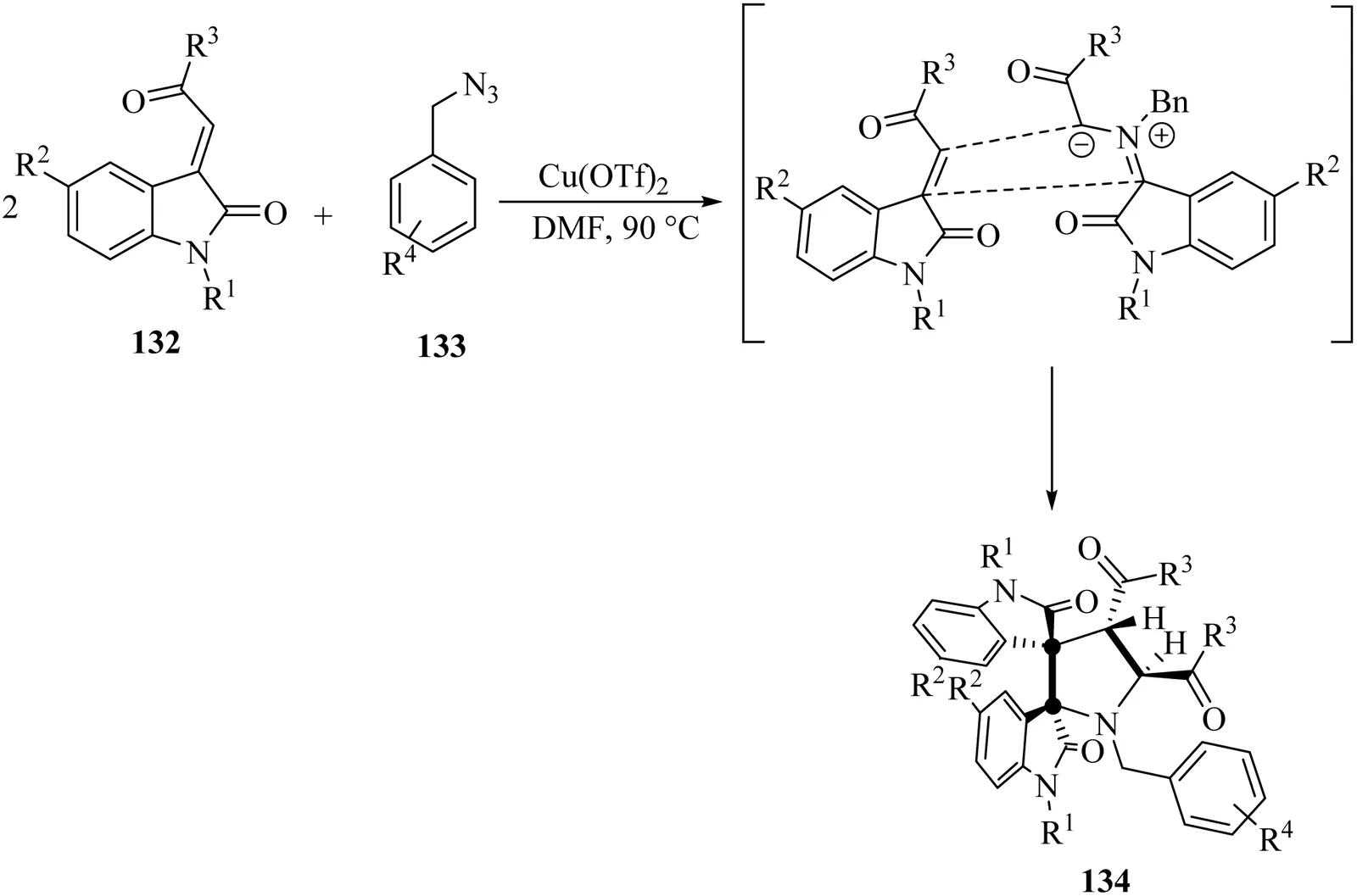
Lewis acid catalyzed synthesis of 3,3′-pyrrolidinylbisspirooxindoles *via* unprecedented [3+2] cycloaddition.

This process demonstrates excellent regio- and diastereocontrol while providing access to biologically relevant bisspirooxindoles scaffolds through an atom-economical strategy enabled by Lewis acid catalysis.

Sun and his coworkers, in 2014, presented an efficient organocatalytic Michael–Michael cascade approach for the enantioselective synthesis of spirocyclopentane bisoxindoles 137, a class of structurally complex heterocycles with potential biological activity. The reaction proceeds under mild conditions using dichloroethane (DCE) as the solvent and a bifunctional chiral squaramide 138 as the catalyst, affording the desired products in moderate to good yields. This strategy enables the precise control of four contiguous stereocenters, including two quaternary all-carbon spiro centers (Scheme 33). Furthermore, the catalytic system provides high diastereoselectivity and excellent enantioselectivity.^85^

**Scheme 33:**
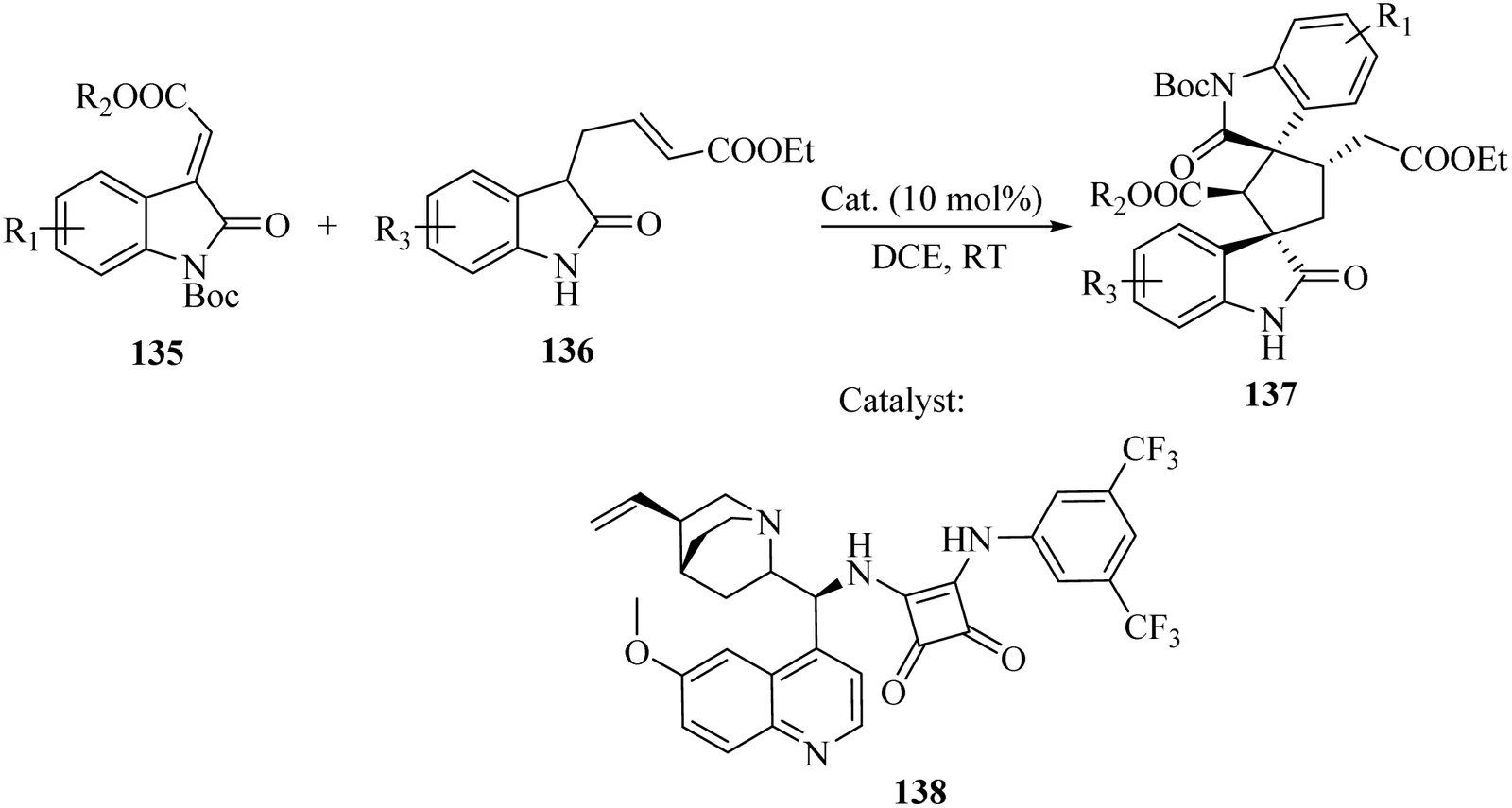
Enantioselective construction of spirocyclopentane bisoxindoles.

Sun *et al.* reported a convenient synthetic method for the preparation of novel bisspirocyclopentane-bisoxindoles 141–143 with high diastereoselectivity, forming four or five stereogenic centers and demonstrating potential applications in medicinal chemistry. The reaction proceeds through a cascade involving Michael addition, aldol condensation, nucleophilic substitution, and reduction steps between two molecules of 3-phenacylideneoxindole 139 (Scheme 34). The method offers several notable advantages, including readily accessible starting materials, mild reaction conditions, operational simplicity, and broad substrate compatibility.^86^

**Scheme 34:**
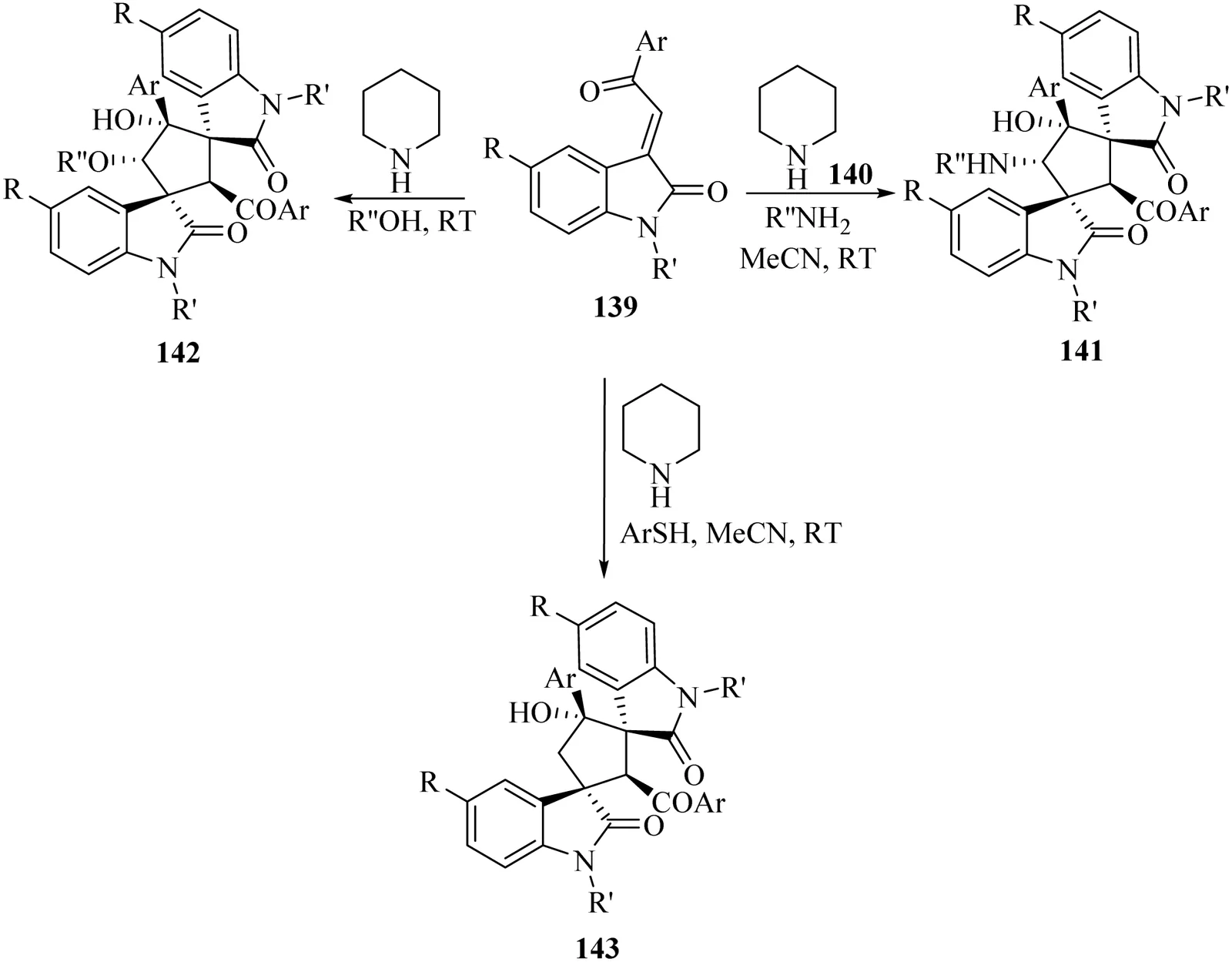
Synthesis of bisspirocyclopentanebisoxindoles *via* self-domino Michael-aldol reactions.

In 2009, a novel dipolar [3+2] cycloaddition approach was developed by Shvets's research group for the efficient synthesis of structurally diverse β,β-spiropyrrolidinooxindoles 146 (Scheme 35). The reaction involves isatin-derived methylene-active heterocycles 144 and azomethine ylides 145 generated *in situ* from sarcosine and paraformaldehyde. The process proceeds smoothly under heating in toluene to afford bisspiro heterocycles 146 in near-quantitative yields with excellent diastereoselectivity, as no other stereoisomers were detected by nuclear magnetic resonance (NMR) spectroscopy. Mechanistically, the cycloaddition occurs from either faces of the ylide, leading to racemic mixtures of 146. This approach enables efficient construction of sterically congested bisspirooxindole frameworks using readily available precursors without chromatographic purification. The new spiro derivatives retain biological relevance owing to their structural analogy to natural spirooxindoles exhibiting antitumor and antibacterial activities.^87^

**Scheme 35:**
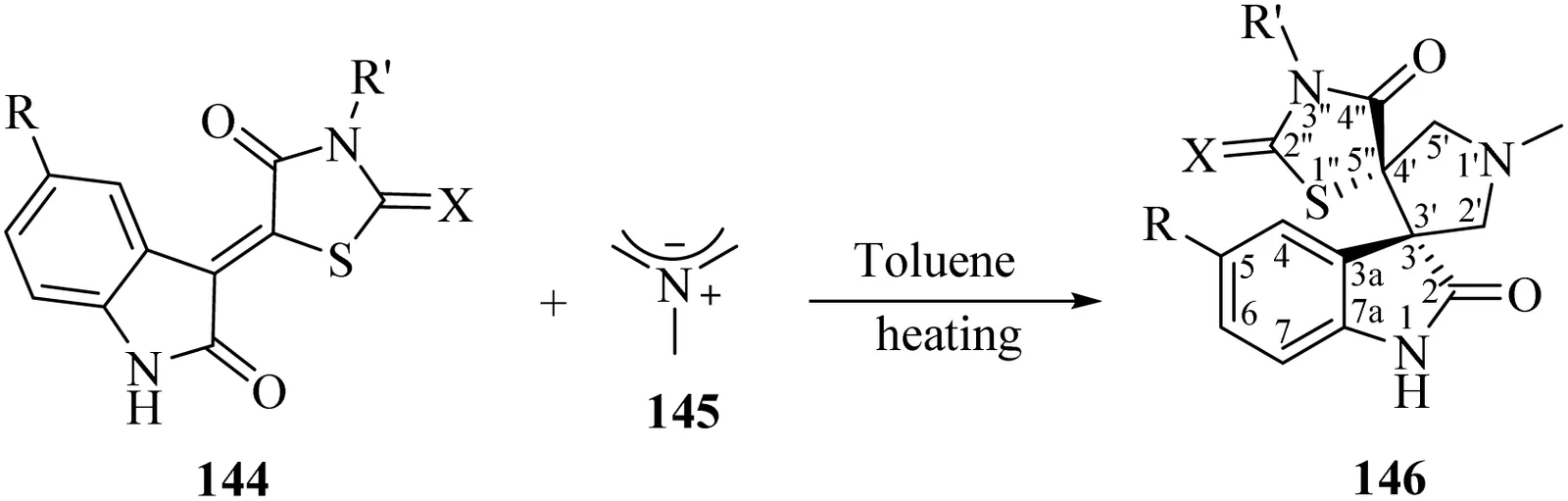
Synthesis of β,β′-spiropyrrolidinooxindoles *via* dipolar [3+2] cycloaddition.

## 3-Alkyl-oxindole-mediated bisspirooxindole synthesis

3-Alkyl-Oxindole have emerged as attractive starting materials for the synthesis of bisspirooxindole derivatives. Although the alkyl group generally exhibits low intrinsic reactivity, appropiriate functionalization can enabled them to the direct transformation of 3-alkyl-oxindole substrates into structurally diverse bisspirooxindole frameworks. Although only a limited number of methodologies have been reported, the representative studies discussed in this section highlight the feasibility and synthetic potential of strategies based on 3-alkyl-oxindole for bisspirooxindole construction.

In 2019, the construction of spirooxindole-fused spiropyrazolones 149*via* an asymmetric [3+2] cyclization reaction between 2-(1-methyl-2-oxoindolin-3-yl)malononitriles 147 and unsaturated pyrazolones 148 was reported by Lin and his co-workers.^88^ Generally, the synthesis of these bisspirodispiro compounds is challenging because it involves the formation of multiple stereocenters, including quaternary stereocenters, under mild and efficient conditions. However, in this strategy, the desired products were obtained through a cascade process involving Michael addition followed by intramolecular cyclization reaction, the desired products were obtained in affording high yields (up to 97%) with excellent enantioselectivities (up to >99% ee) and diastereoselectivities (>20 : 1 dr). This methodology also offers a valuable platform for the development of compounds with potential medicinal applications. Remarkably, bifunctional squaramide organocatalysts 135 play a crucial role in activating both reaction partners through hydrogen-bonding interactions, leading to excellent stereoinduction and high reaction efficiency at very low catalyst loading, while squaramide catalystis regenerated during the reaction (Scheme 36).

**Scheme 36:**
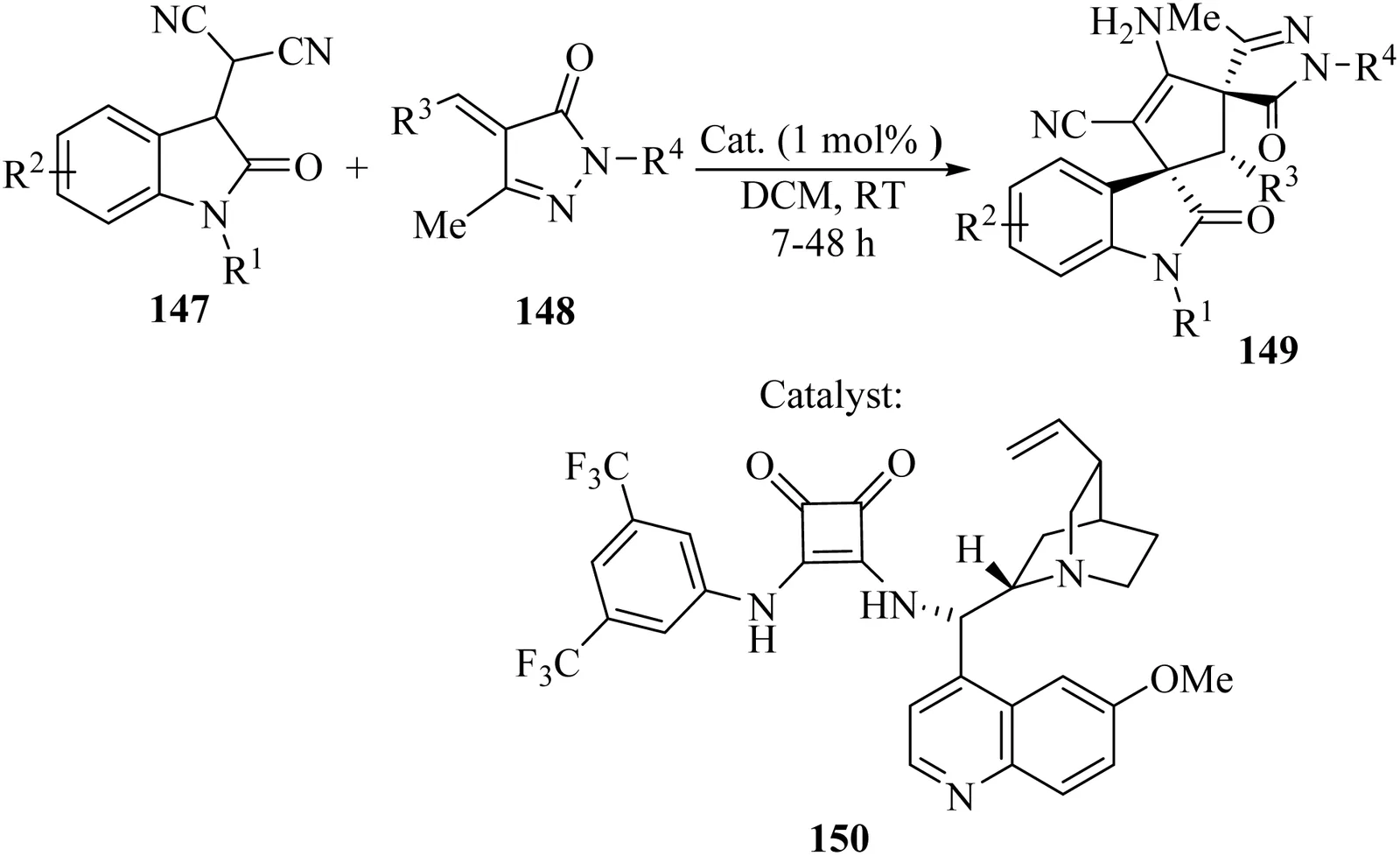
Bifunctional squaramide-catalyzed synthesis of spirooxindole-fused spiropyrazolones.

Bisspirooxindole-based polycyclic frameworks represent an important class of structurally rigid motifs frequently encountered in bioactive and medicinally relevant molecules. In 2019, Liu and co-workers developed a bifunctional oxindole–chromone synthon-directed thioamidation cascade reaction, enabling the rapid and stereocontrolled assembly of highly complex bisspirooxindole frameworks 153. The final products, hexahydroxanthone-based derivatives 153 bearing two spirooxindole units and five contiguous stereocenters, were obtained in excellent yields (up to 91%), with high diastereoselectivities (>20 : 1 dr) and excellent enantioselectivities (up to 99% ee). Preliminary biological evaluation showed that selected compounds exhibited moderate antiproliferative activity against K562 cancer cells, highlighting the medicinal potential of these structurally rigid bisspirooxindole derivatives.

Significantly, the observed stereocontrol arises from a donor/acceptor-based oxindole–chromone system that effectively controls the spatial arrangement of the reacting partners, thereby enabling the selective formation of multiple contiguous stereogenic centers without the involvement of chiral transition-metal catalysis (Scheme 37).^89^

**Scheme 37:**
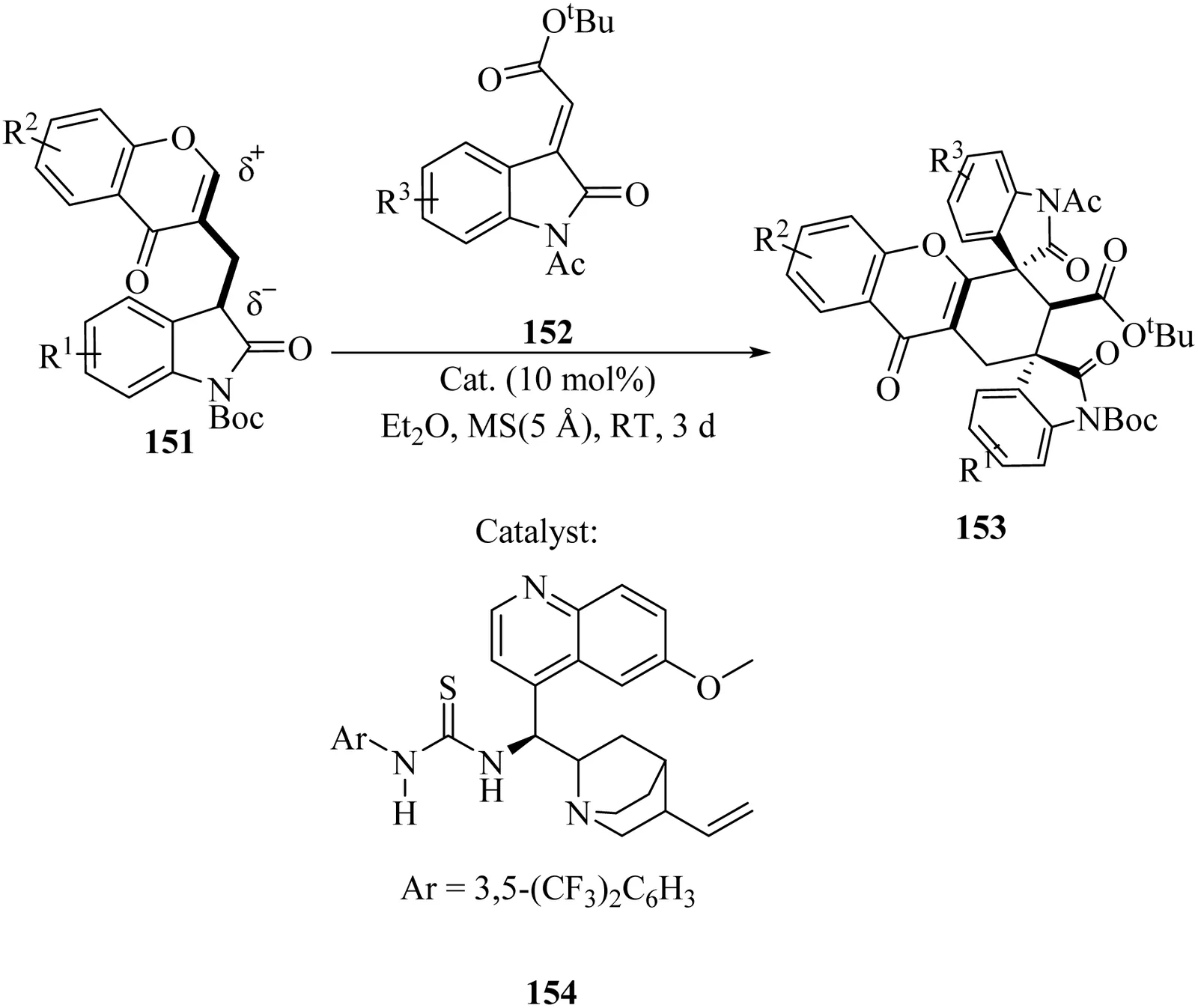
Synthesis of bisspirooxindole-based hexahydroxanthones bearing five contiguous stereocenters.

The construction of sterically congested arylated quaternary stereogenic centers represents a fundamental challenge in asymmetric synthesis because of severe steric hindrance and limited activation modes.^90^ Such highly substituted stereocenters are prevalent structural motifs in a wide range of biologically active natural products and structurally complex synthetic analogues, highlighting the continued need for efficient and highly stereoselective synthetic strategies for their. In 2018, Qiao *et al.* reported an asymmetric organocatalytic direct arylation strategy between quinones 155 and 3-alkyloxindoles 156. This transformation is particularly challenging because the competitive oxidation of hydroquinones to quinones typically prevents access to the desired arylated hydroquinone products. Nevertheless, the developed method provides access to a wide range of structurally and stereochemically complex heterocyclic frameworks, including fused 159, spiro 161–162, and bisspiro 163 heterocycles, highlighting its synthetic and medicinal relevance (Scheme 38).^91^

**Scheme 38:**
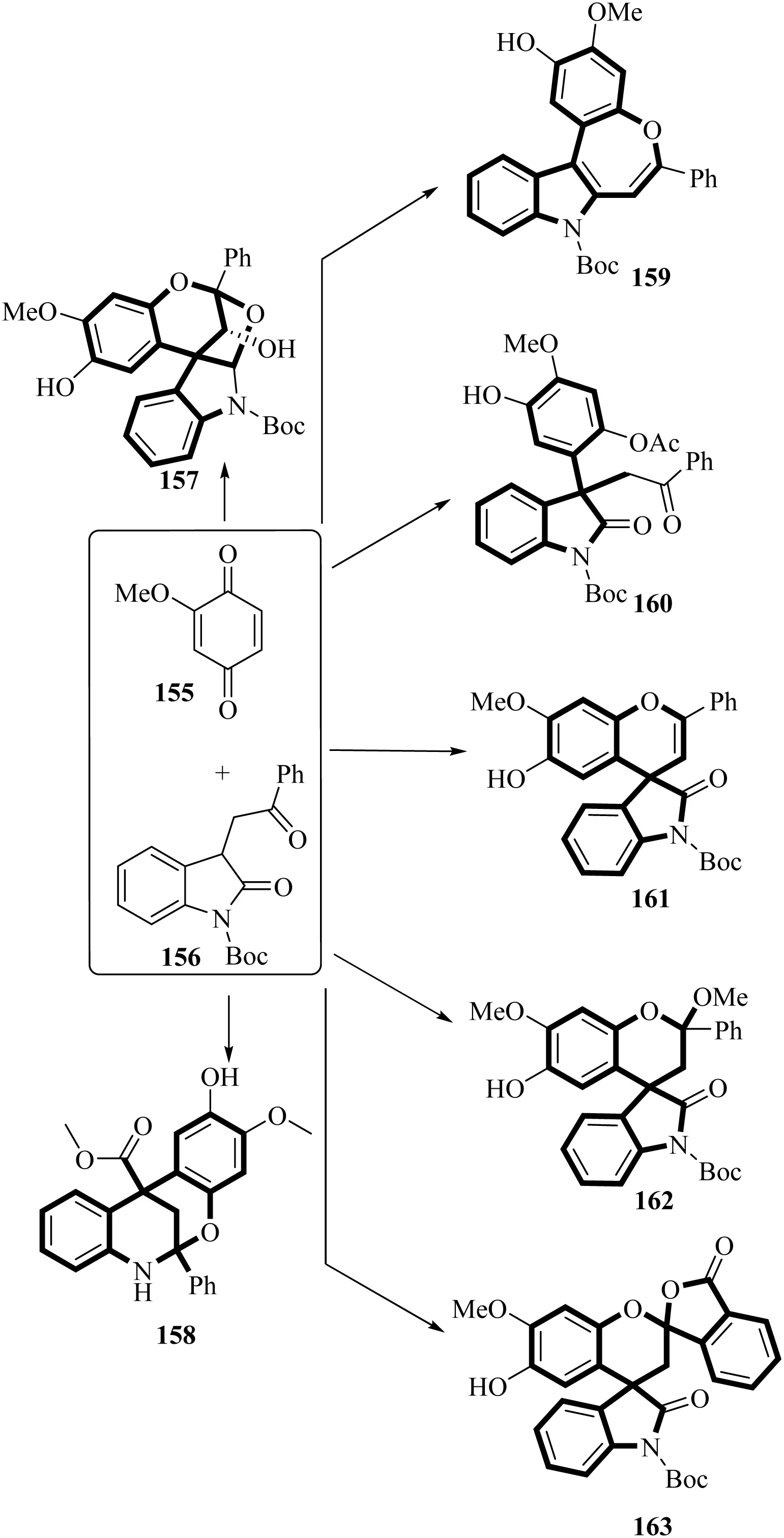
Organocatalytic asymmetric synthesis of complex heterocycles.

In this strategy, the introduction of a ketone carbonyl group leads to the *in situ* formation of a hemiketal intermediate. This key design element stabilizes the hydroquinone intermediate and suppresses undesired oxidation pathways. The transformation proceeds efficiently under mild organocatalytic conditions with a low catalyst loading of 1 mol%, exhibiting excellent operational simplicity while enabling precise stereochemical control over the formation of a sterically congested aryl-substituted quaternary stereogenic center.

In 2017, a catalyst-free protocol for the synthesis of 3,3′-spirooxindole γ-butyrolactones 165, 167 was developed through formal [3+2] cyclization reaction. Utilizing indolin-2-one-derived aliphatic acids 164 as a readily accessible substrates and isatins 15 as electrophiles, the transformation proceeded efficiently *via* an *in situ* activation strategy, providing access to structurally diverse 3,3′-spirooxindole γ-butyrolactones scaffold 165 in moderate to excellent yields with good diastereoselectivities (Scheme 39). Notably, the resulting spirooxindole γ-butyrolactones architectures exhibit distinctive structural features that may render them attractive candidates for future drug discovery and medicinal chemistry applications.^92^

**Scheme 39:**
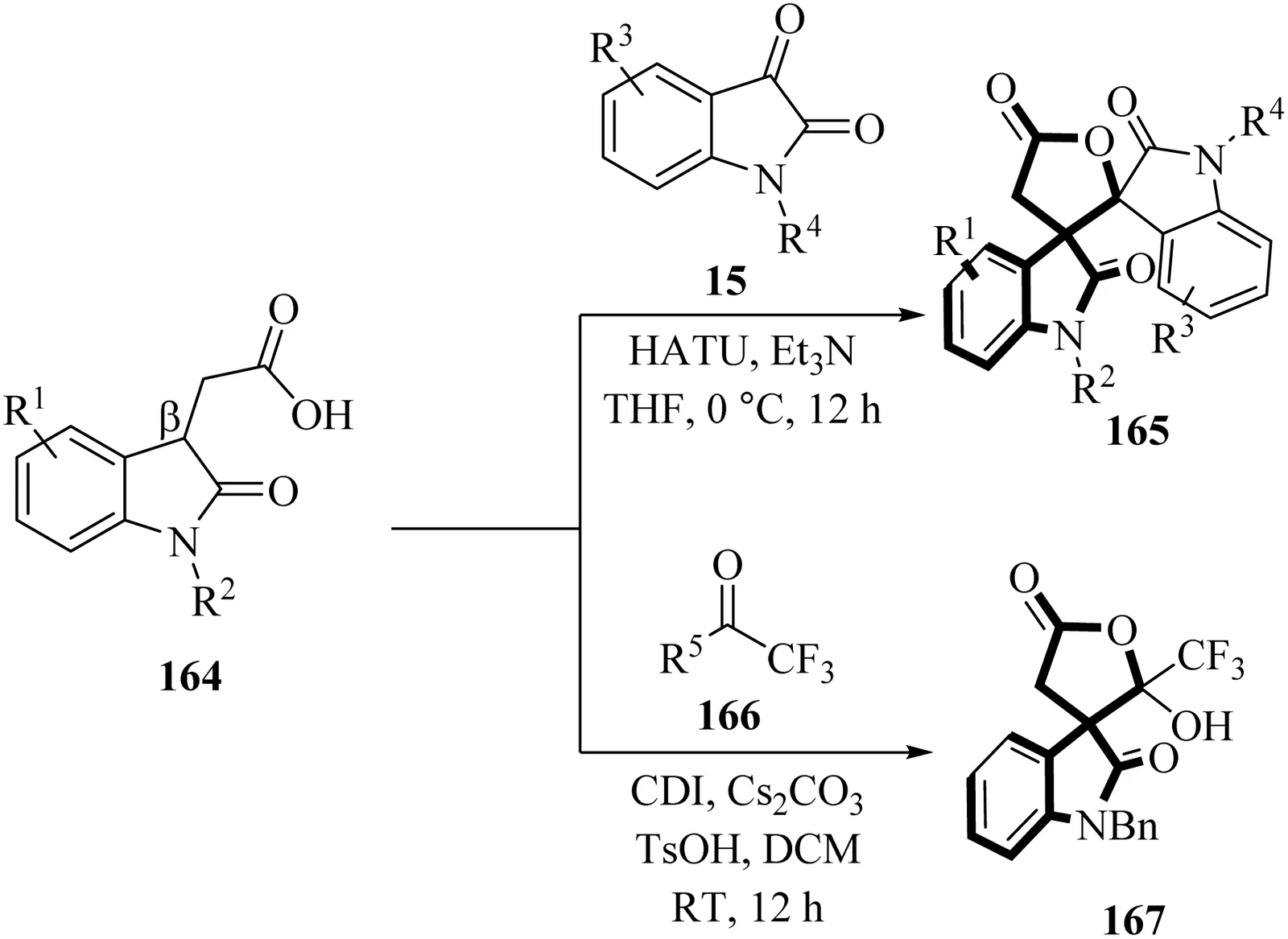
Divergent synthesis of spirooxindole γ-butyrolactones.

## Isatin-derived Morita–Baylis–Hillman (MBH) carbonates approaches to bisspirooxindole

Isatin-derived Morita–Baylis–Hillman (MBH) carbonates are versatile allylic systems that have attracted considerable attention as synthetic substrates for the construction of bisspirooxindole scaffolds. Owing to their unique structural features, they readily participate in a variety of cyclization reactions enabling the efficient construction of structurally diverse bisspirooxindole frameworks.

In 2023, the Sun group reported a convenient base-promoted cyclization strategy for the synthesis of diverse functionalized spiro-indanedione-oxindole scaffolds fused with dihydrobenzofuran units 170–171, employing Morita-Baylis–Hillman (MBH) carbonates of isatins 168 and 2-(*o*-hydroxybenzylidene)-1,3-indanediones 169 as starting materials. In this study, they used mild organic bases, including 1,4-diazabicyclo[2.2.2]octane (DABCO) and 4-Dimethylaminopyridine (DMAP), which facilitated cyclization reactions with high regio- and diastereoselectivity, yieliding structurally diverse products, including spiro[cyclopropa[*c*]chromene-1,2′-indene]-1′,3′-diones 170 and bisspiro[indene-2,1′-cyclopenta[*b*]benzofuran-2″,3″-indolines] 171 (Scheme 40).^93^

**Scheme 40:**
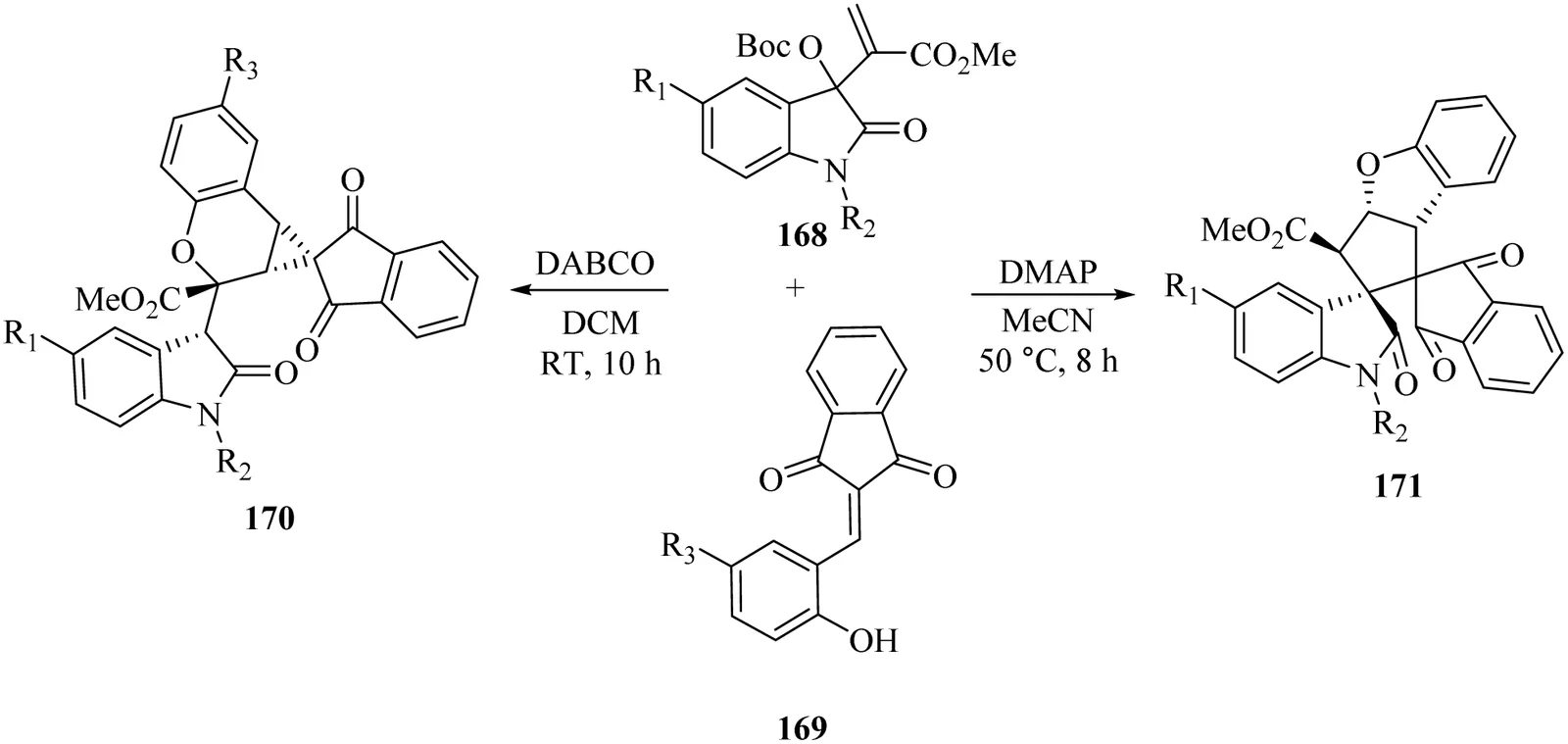
Synthesis of functionalized spiro-indanedione-oxindole scaffolds fused with dihydrobenzofuran units.

Additionally, a similar strategy involving MBH maleimides and isatins enabled the efficient synthesis of bisspiro[indene-2′,3′:1,5]cyclopenta[1,2-*c*]pyrrole-4′,3″-indolines. Moreover, substituents on the isatin core had minimal effects on the reaction outcome, whereas chloro- and bromo-substituted *o*-hydroxybenzylidene rings generally afforded lower yields compared with their methyl-substituted counterparts.

In 2023, Dan Liu, Jing Sun, and colleagues examined a regio- and diastereoselective base-promoted [3+3] cyclization reaction between bindone 172 and isatin-derived MBH carbonates 173 enabaling structural diversity through variation of the reaction conditions. Using DABCO as the base in DCM at room temperature, the reaction afforded the corresponding bisspiro[indene-2,4′-fluorenone-1′,3″-indoline] derivatives 174 in good yields and with high diastereoselectivity (Scheme 41, Path A).

**Scheme 41:**
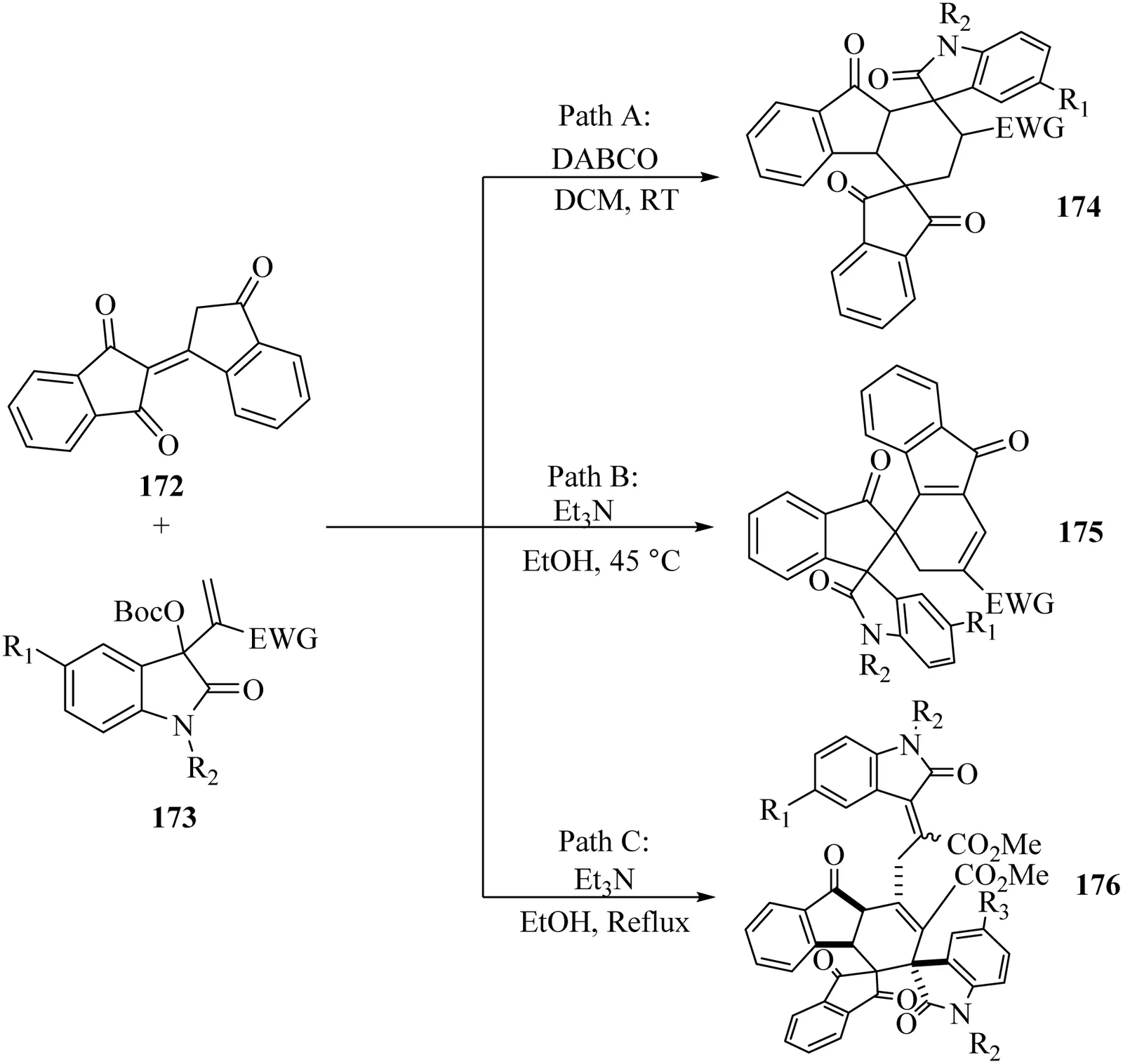
Divergent synthesis of bisspiro frameworks *via* [3+3] cyclization reaction.

Under another condition, triethylamine was used in EtOH at 45 °C, and the cyclization reaction between 1,3-indanediones and isatin-derived MBH esters generated bisspiro[indene-2,4′-fluorenone-3′,3″-indoline] compounds 175 in moderate yields (Scheme 41, Path B). Notably, the refluxing EtOH conditions with triethylamine, the reaction with an excess 1,3-indanediones selectively formed the Z-isomer 176 as the major product, while the E-isomer was afforded as the minor one (Scheme 41, Path C). This study demonstrates the significant influence of reaction temperature, base, and substrate structure on the regioselectivity and stereoselectivity of the transformation. Furthermore, the potential applications of this methodology in heterocyclic and medicinal chemistry are particularly noteworthy.^94^

In 2022, Prakash K. Warghude, Anindita Bhowmick, and Ramakrishna G. Bhat reported a highly regio- and diastereoselective [3+2] cyclization method involving Morita–Baylis–Hillman (MBH) carbonates of isatins 178 and pyrazolones with ketimine derivatives 177 to produce a new class of heterocyclic compounds 179.

Pyrazolone-derived ketimines have recently been used as valuable starting materials in asymmetric synthesis that can serve as excellent electrophiles at C-4 position. In this work, the authors developed an efficient cyclization between MBH carbonates and ketimine derivatives to synthesize spirooxindole dihydropyrrole-fused pyrazolone derivatives 179 and bisspiropyrazolone derivatives 181. The final products feature two vicinal quaternary spiro centers and were obtained in good to excellent yields with very high diastereoselectivity under mild catalytic conditions using DMAP (4-dimethylaminopyridine) at room temperature (Scheme 42).^95^

**Scheme 42:**
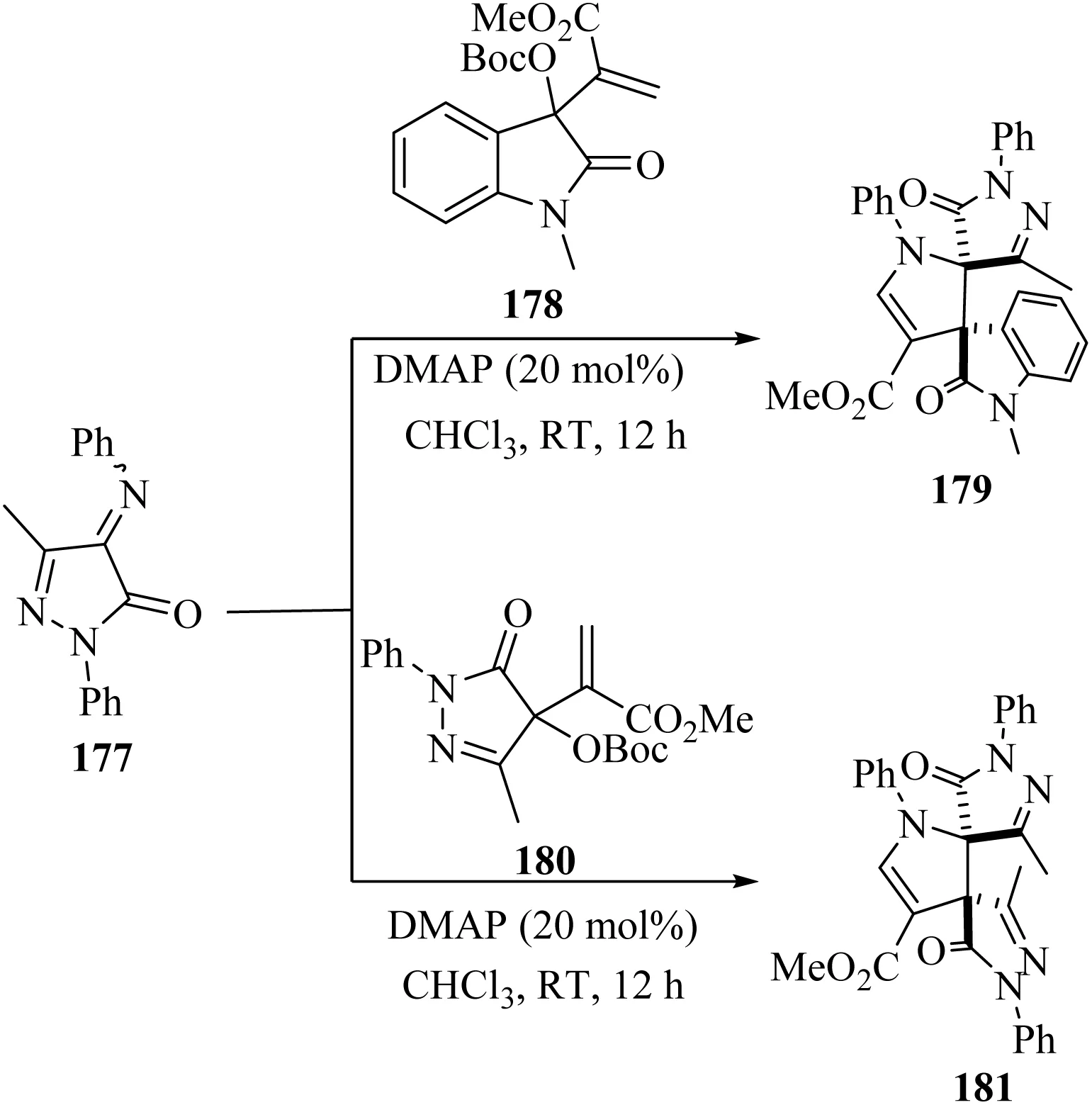
[3+2] cyclization method for the synthesis of new bisspiro compounds.

In this 2022 study, Lokesh and colleagues developed a highly efficient and diastereoselective [4+2] cyclization reaction between isatin-derived Morita–Baylis–Hillman (Is-MBH) alcohols 182 and alkylidene pyrazolones 183 to construct novel tetrahydro-dispiro[indolinone-3,2′-pyran-5′,4″-pyrazolone] scaffolds 184. Remarkably, this metal-free strategy represents the first reported application of Is-MBH alcohols as oxa-Michael donors, providing efficient access to two quaternary and two tertiary stereogenic centers *via* a tandem Michael addition reaction followed by subsequent intramolecular cyclization (Scheme 43).^96^

**Scheme 43:**
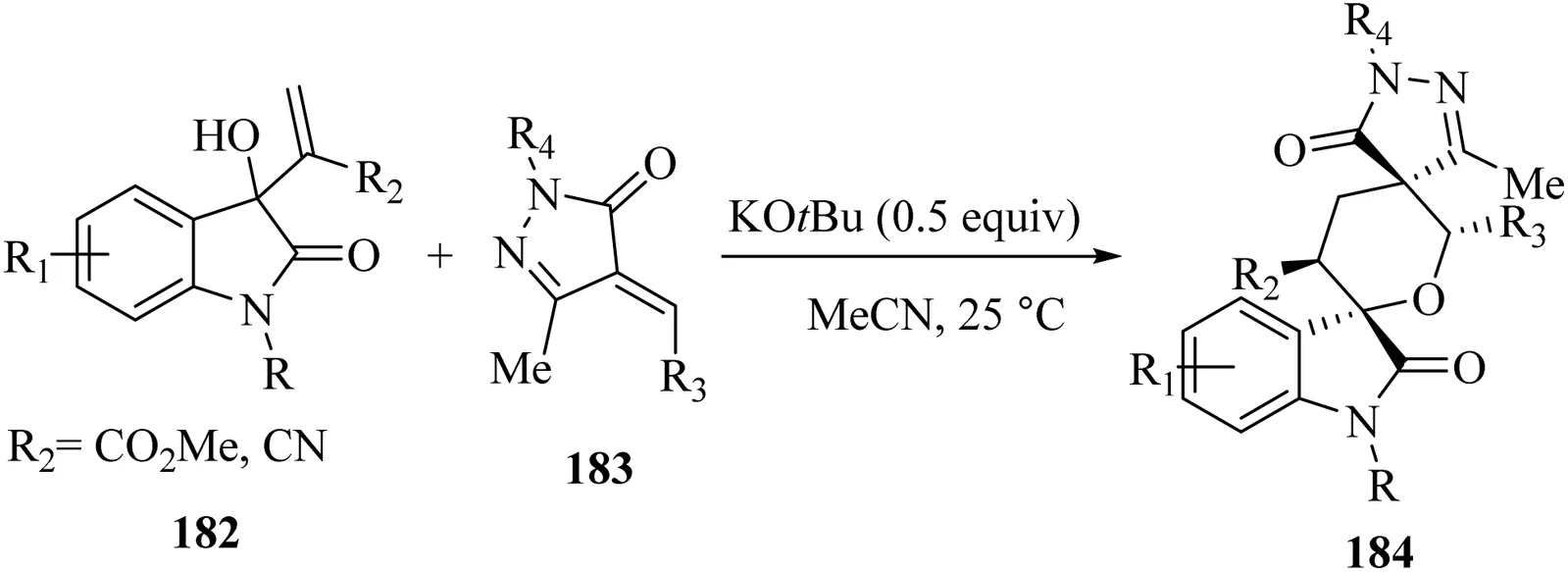
Construction of novel tetrahydro-bisspiro[indolinone-3,2′-pyran-5′,4″-pyrazolone] scaffolds *via* [4+2] cyclization reaction.

In 2022, Tian and his team developed a chiral phosphine-catalyzed asymmetric α-regioselective [3+2] cyclization between Morita–Baylis–Hillman (MBH) carbonates 168 and 4-arylmethylisoxazol-5-ones 185. This metal-free strategy proceeds under mild reaction conditions using a chiral phosphine catalyst 187, affording excellent yields (up to 99%) with high regio-, diastereo-, and enantioselectivity (d.r. >19 : 1; ee up to 99%). Importantly, this method enables the efficient construction of spirocyclic oxindole derivatives 186 bearing three contiguous stereogenic centers, including vicinal all-carbon quaternary chiral centers (Scheme 44).^97^

**Scheme 44:**
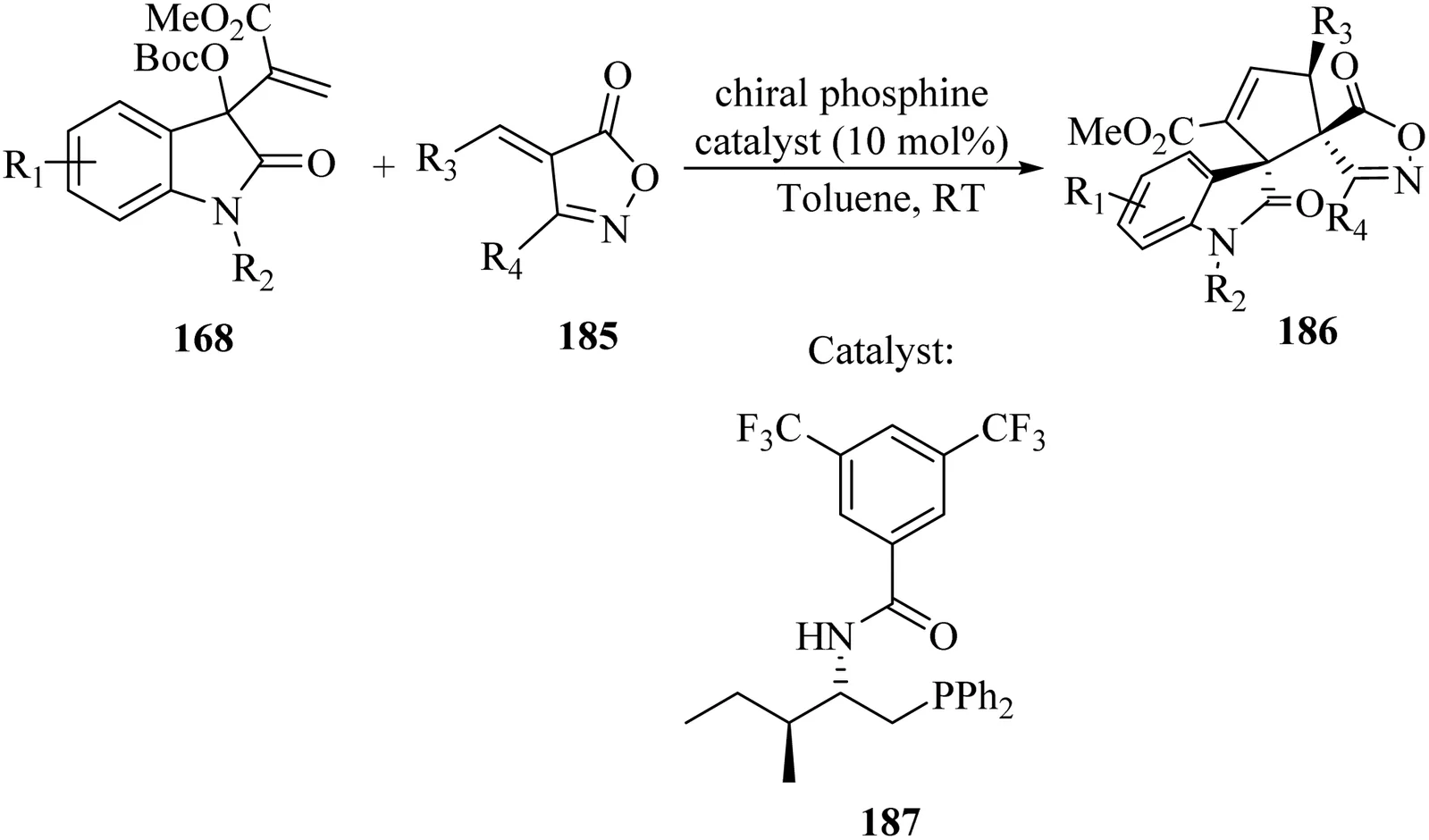
Synthesis of spirocyclic oxindole derivatives *via* a chiral phosphine-catalyzed asymmetric regioselective [3+2] cyclization reaction.

In 2021, Mei and his coworkers reported an asymmetric [3+2] cyclization reaction between isatin-derived Morita–Baylis–Hillman (MBH) carbonates 188 and 5-alkenylthiazol-4(5H)-ones 189, resulting in the generation of bisspirooxindole derivatives with three contiguous stereogenic centers. One of the most notable features of this work is the catalyst-controlled modulation of diastereoselectivity: employing an amide-phosphine catalyst 190 directs the reaction to one diastereomer 191, while using a DMAP-thiourea catalyst 192 leads to the opposite diastereomer 193. Furthermore the authors realized that the additive HFIP (hexafluoroisopropanol) played a key role in accelerating the reaction and enhancing the diastereoselectivity (Scheme 45).^98^

**Scheme 45:**
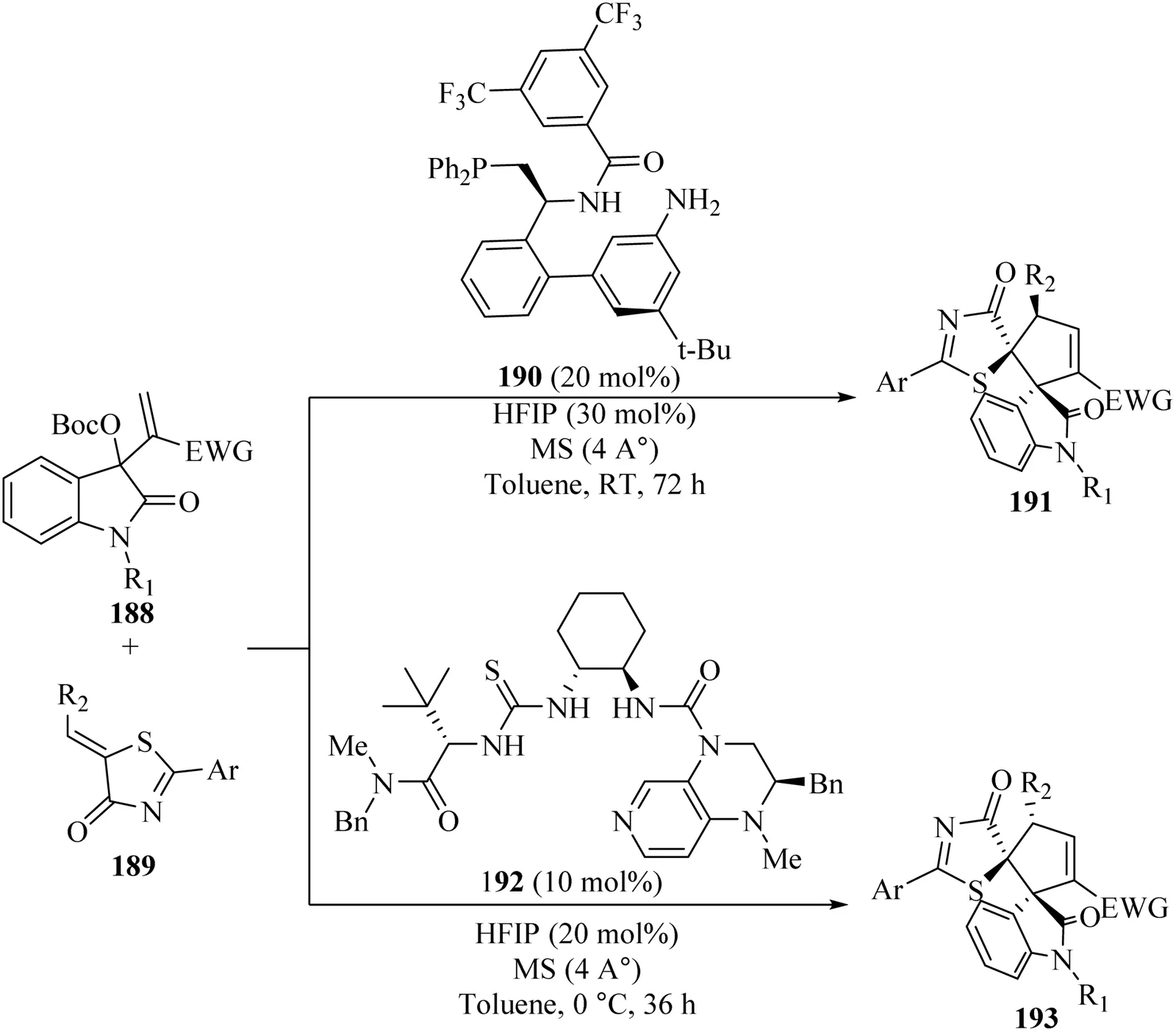
Construction of bisspirooxindole derivatives *via* [3+2] cyclization reaction between isatin-derived Morita–Baylis–Hillman (MBH) carbonates and 5-alkenylthiazol-4(5*H*)-ones.

Chen and his team, in 2018, described a highly regio- and stereoselective [3+2] cycloaddition reaction between isatin-derived Morita–Baylis–Hillman (MBH) carbonates 168 and 3-methyleneoxindoles 194, leading to novel 3,3′-cyclopentenyldi- spirooxindoles 195 featuring two adjacent quaternary spirostereocenters. The reaction is catalyzed by a chiral tertiary amine (B-isocupreidine as the nucleophile) 196 under mild conditions, affording the bisspiro products in high yields (Scheme 46).^99^

**Scheme 46:**
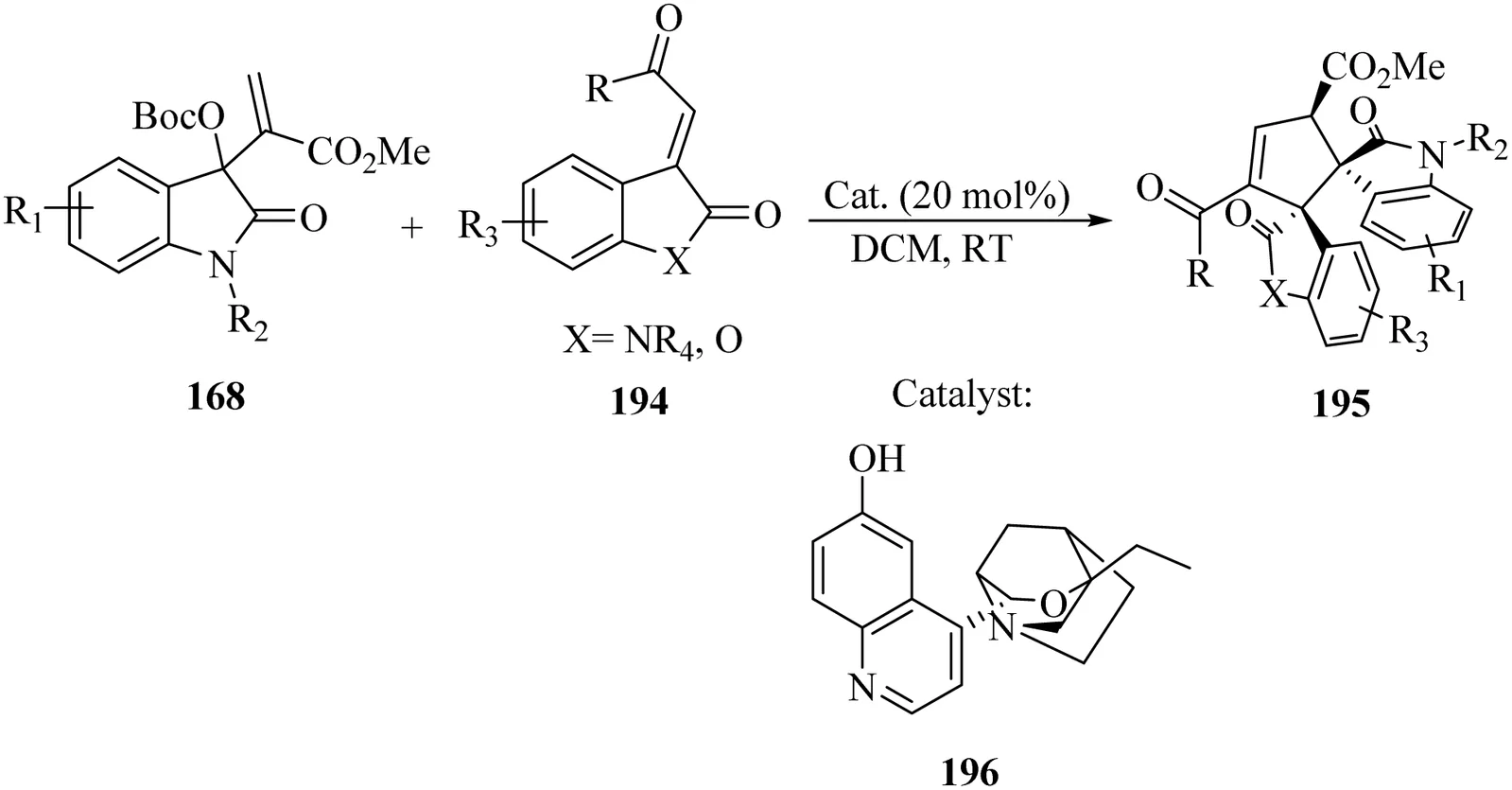
Synthesis of 3,3′-cyclopentenylbisspirooxindoles *via* 1,3-dipolar cycloaddition.

In 2014, Warghude *et al.* reported a catalyst-controlled strategy under mild reaction conditions for the construction of two distinct classes of spirooxindole derivatives *via* cycloaddition reactions between isatin-derived Morita–Baylis–Hillman (MBH) carbonates 168 and 3-methyleneoxindoles 197. In the first pathway (Scheme 47, Path A), using a chiral tertiary amine catalyst (DMAP), the reaction proceeds through a [3+2] cycloaddition, generating cyclopentenyl bisspirooxindoles 198. In contrast, employing a different catalyst directs the reaction through a [2+1] cycloaddition pathway (Scheme 47, Path B), leading to the formation of cyclopropyl spirooxindoles 199. Both pathways exhibit high diastereoselectivity and result in the formation of three contiguous stereocenters, with the desired products resulting in good to excellent yields.^100^

**Scheme 47:**
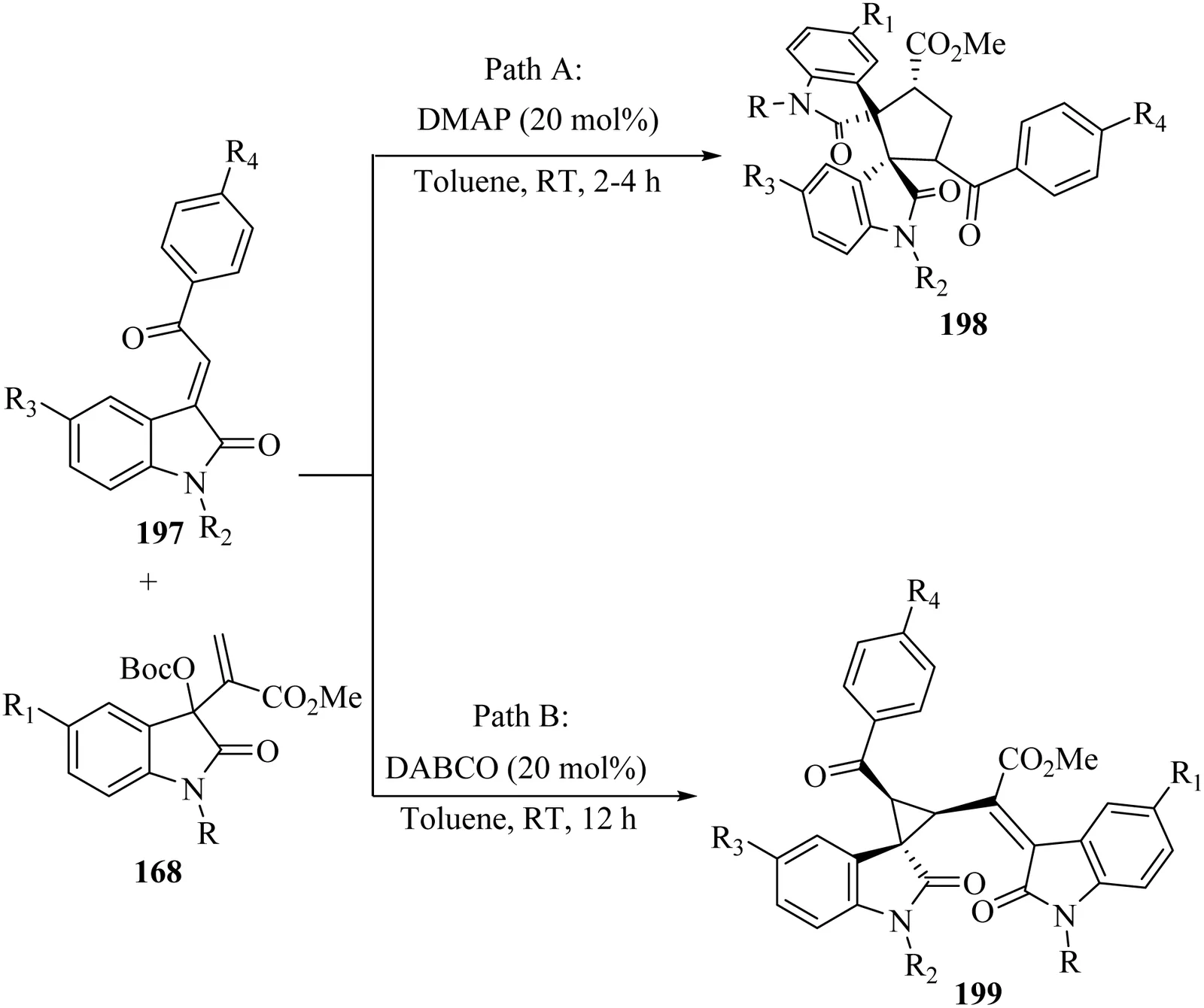
Synthesis of cyclopropyl spirooxindoles 198 and cyclopentenyl bisspirooxindoles 199*via* cycloaddition reactions.

## 3-Halo-oxindole-based precursors for bisspirooxindole formation

3-Halooxindoles have emerged as versatile and valuable synthetic intermediates in modern heterocyclic chemistry. Owing to the presence of a halogen substituent at the C-3 position adjacent to the carbonyl group, 3-halooxindoles display enhanced reactivity and readily participate in cyclization reactions with functionalized alkenes, enabling the efficient synthesis of structurally diverse bisspirooxindole-cyclopropane frameworks.

In 2023, Eskandari and his team reported a new approach for the asymmetric synthesis of bisspirocyclopropyloxindole frameworks using 3-chlorooxindole derivatives 200 as the starting substrates. This substrate-controlled approach, guided by a chiral auxiliary, enabled the construction of four distinct stereoisomers: rel-(1R,2R,3R), rel-(1S,2S,3R), rel-(1R,2R,3S), and rel-(1S,2S,3S) 202–205, all obtained in excellent enantiopurity and high yields. Remarkably, the absolute configurations of these compounds, featuring three contiguous quaternary/tertiary stereogenic centers, was determined by X-ray crystallography. In addition, the authors introduced a new and efficient synthetic approach for the preparation of 3-chlorooxindole derivatives, which serve as valuable intermediates in organic synthesis (Scheme 48).^101^

**Scheme 48:**
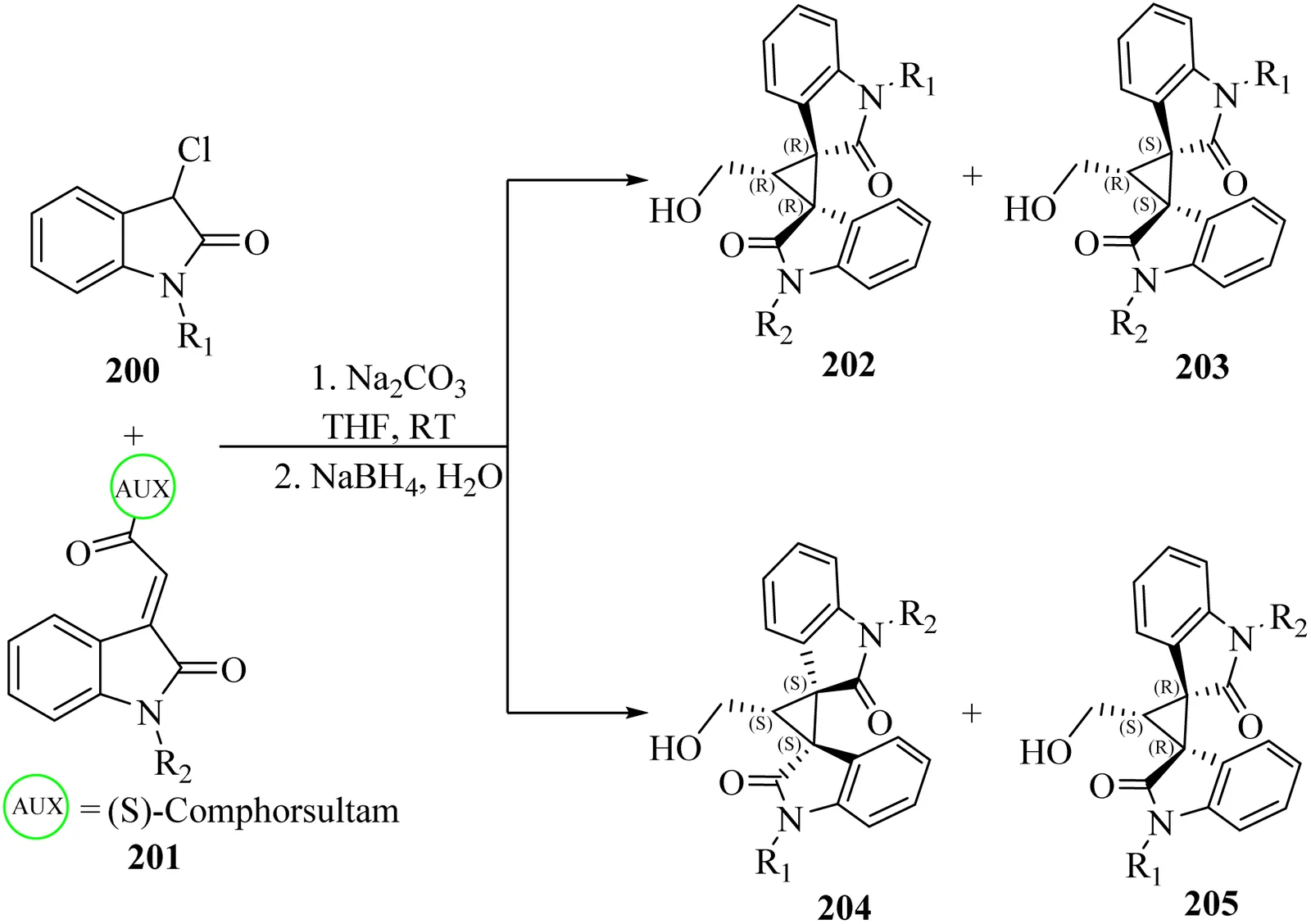
Diastereo- and enantioselective synthesis of bisspirocyclopropyloxindoles.

In 2022, the Hao group proposed a novel strategy for the enantioselective and diastereoselective synthesis of bisspiro[indanedione-oxindole-cyclopropane] compounds 207*via* a squaramide-catalyzed [2+1] cycloaddition reaction between 2-arylidene-1,3-indanediones 90 and 3-bromooxindoles 206, affording the corresponding products in high yields (Scheme 49). The formation of an ammonium ylide intermediate through intramolecular cyclization was confirmed by high-resolution mass spectrometry (HRMS).^102^

**Scheme 49:**
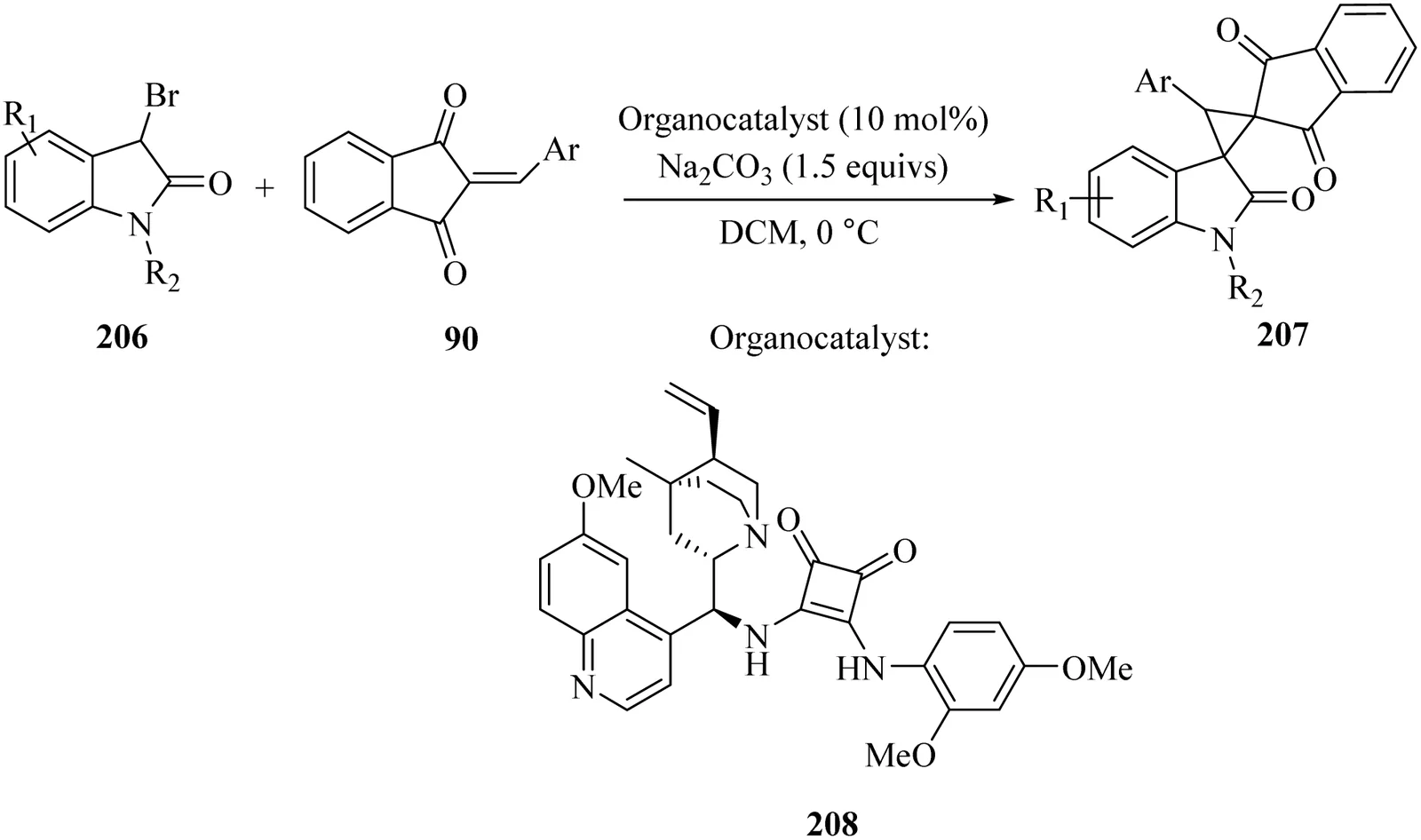
Squaramide-catalyzed [2+1] cycloaddition reaction for the synthesis of bisspiro[indanedione-oxindole-cyclopropane].

A cascade 1,6-conjugate addition/dearomatization reaction of para-quinone methides 209, 210 with 3-chlorooxindoles 211 was reported by Zhang *et al.* for the synthesis of bisspiro cyclopropanes 212 (Scheme 50).^103^ One of the major advantages of the proposed synthetic protocol is the efficient synthesis of the target compounds in high yields within an exceptionally short reaction time of approximately 5 min at room temperature. Furthermore, the synthesized products are expected to exhibit promising biological activity and may therefore serve as potential candidates for further biological evaluation.

**Scheme 50:**
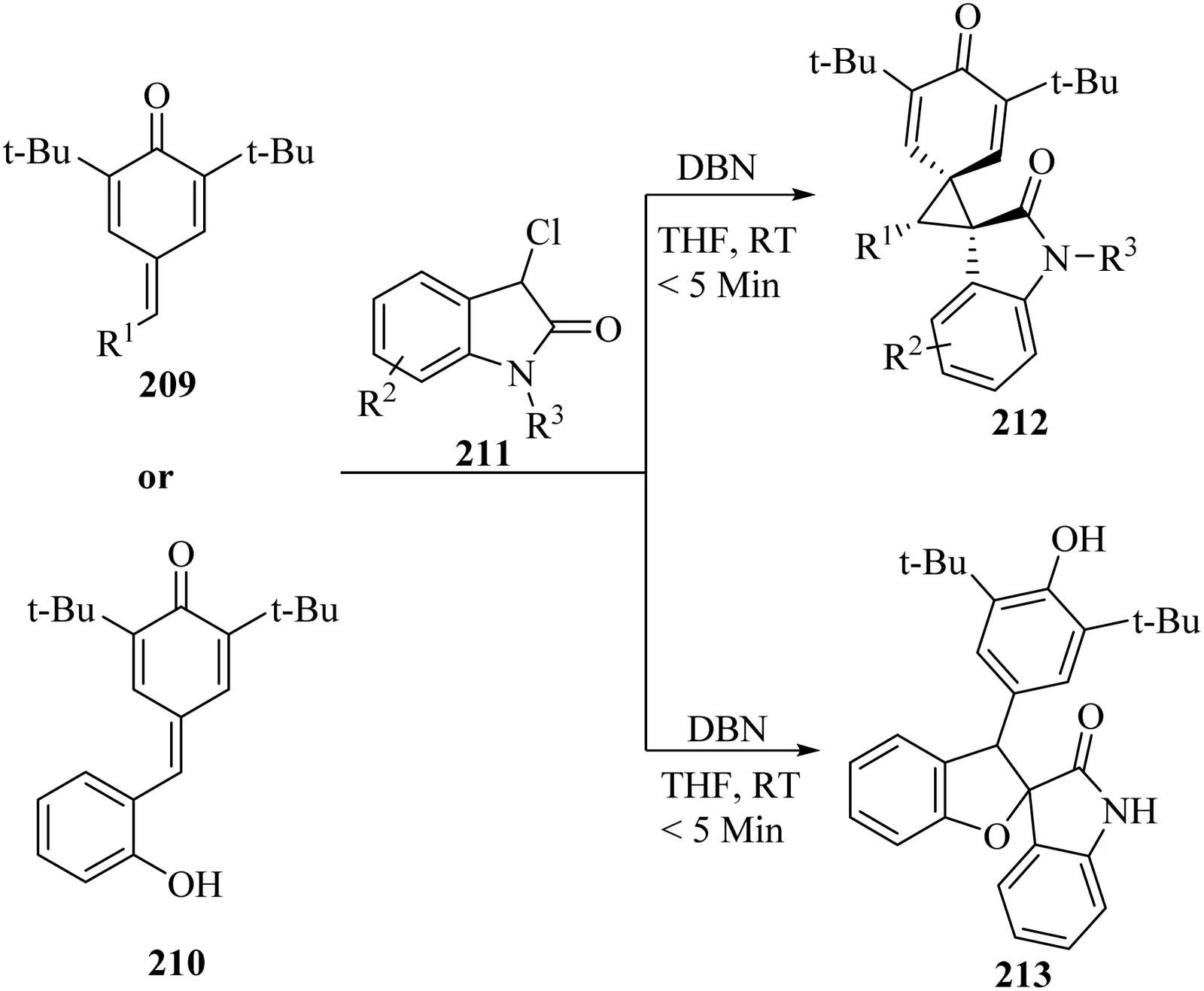
Construction of bisspiro cyclopropanes *via* cascade 1,6-conjugate addition/dearomatization reaction.

In 2018, Ding and Wolf proposed an organocatalytic asymmetric method for the synthesis of α-oxetanyl and α-azetidinyl alkyl halides 216 with excellent diastereo- and enantioselectivities. These compounds act as versatile intermediates for the construction of different multifunctional molecules, involving fluorooxindoles with two contiguous stereocenters, fluorinated heterocyclic spiranes, and polyspiro compounds (Scheme 51).^104^

**Scheme 51:**
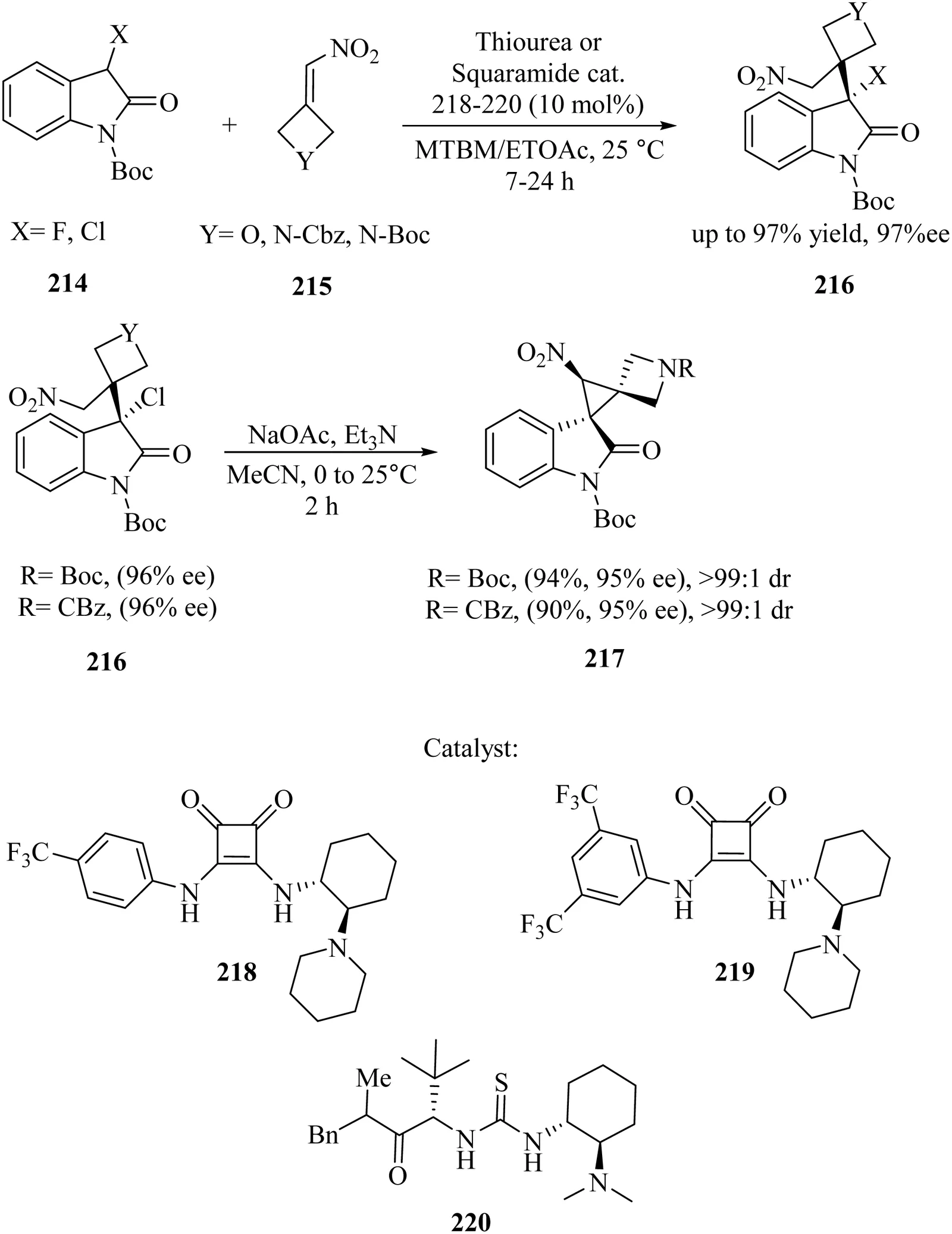
Synthesis of bisspirooxindole *via* azetidinyl tertiary alkyl halides.

Significantly, they achieved the conversion of an oxindole chloride into a bisspiro compound 217*via* intramolecular cyclization, generating a cyclopropane ring in which all three carbon atoms are stereogenic centers. The transformation proceeded with high yield (94%), excellent enantioselectivity (95% ee), and exceptional diastereoselectivity (>99 : 1 dr).

In 2015, Li and his colleagues developed a new strategy for the construction of bisspirooxindole derivatives from arylidene pyrazolones 221 and 3-chlorooxindoles 222*via* a diastereoselective Michael/alkylation cascade reaction. In the first method (Scheme 52, method A), *N*,*N*-diisopropylethylamine (DIPEA) was used as a base, to catalyze the reaction, and this provided a simple and straightforward route to access the desired products in excellent yields. The resulting products feature vicinal quaternary carbon centers within the cyclopropane ring.

**Scheme 52:**
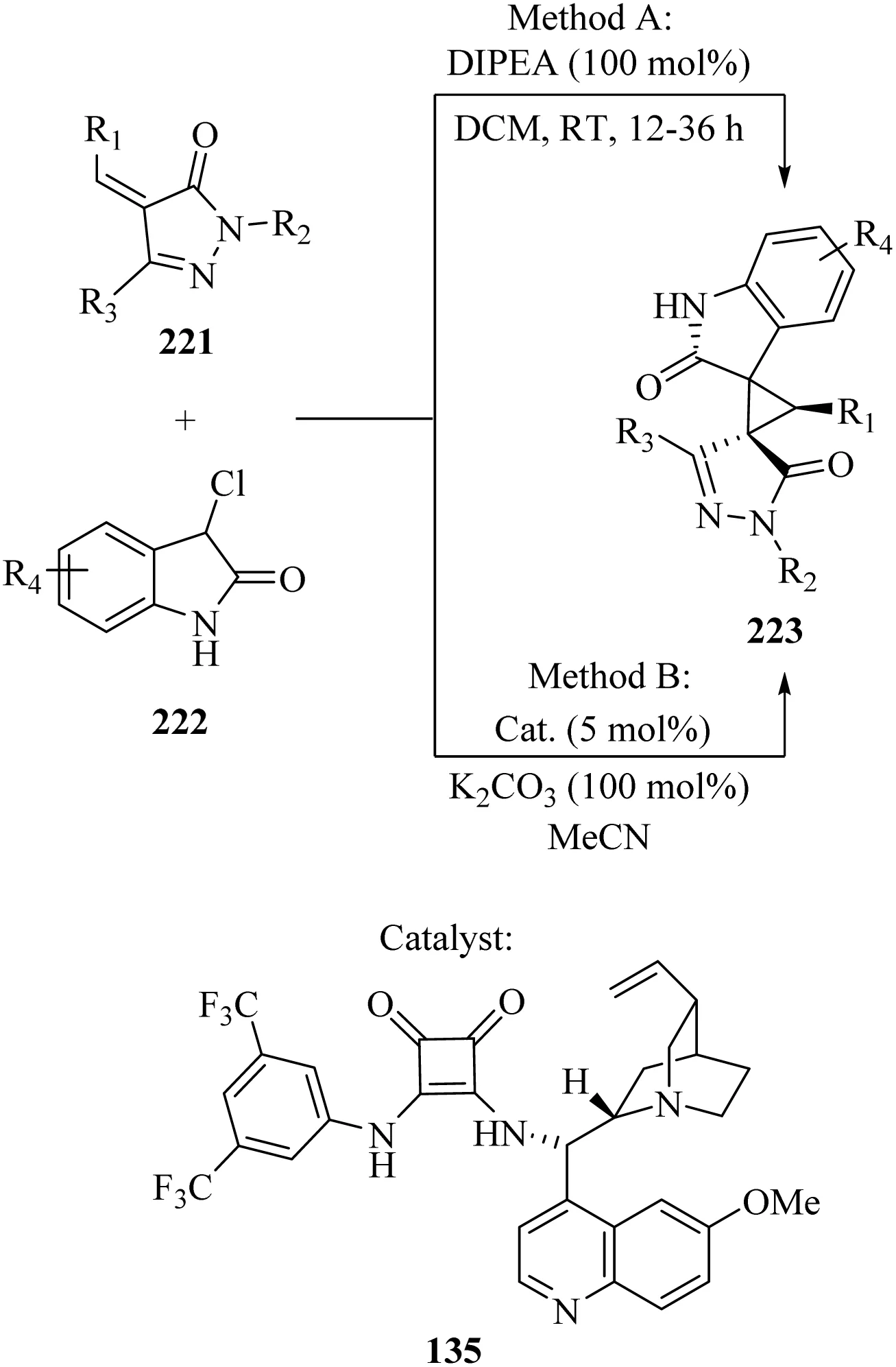
Diastereoselective michael/alkylation cascade reaction of arylidenepyrazolones with 3-chlorooxindoles.

The authors also developed an asymmetric version of this reaction by using a squaramide-based organocatalyst 135 (Scheme 52, method B). This method is practical for constructing complex heterocyclic scaffolds with multiple stereocenters, showing moderate diastereoselectivity (87 : 13 dr) and moderate to high enantioselectivity (up to 74% enantiomeric excess).^105^

## 3-Amino-oxindoles-initiated construction of bisspirooxindole cores

This section reviews the synthesis of bisspirooxindole derivatives from 3-aminooxindoles. Although reports employing 3-aminooxindoles as key starting materials remain relatively scarce, the representative examples discussed herein demonstrate that the combination of appropriate substrates with suitably designed catalytic systems can provide efficient access to bisspirooxindole frameworks. These studies highlight the synthetic potential of this strategy and encourage further exploration of 3-aminooxindoles in bisspirooxindole synthesis.

The 3,2′-pyrrolidinyl spirooxindole frameworks are important structural motifs widely found in biologically active and medicinally relevant molecules.^106–109^ In 2019, Yang *et al.* presented an asymmetric cascade reaction between 3-aminooxindoles 224 and β,γ-unsaturated α-keto amides 225 for the synthesis of chiral 2,5-pyrrolidinyl bisspirooxindoles 226. This transformation is particularly challenging due to the simultaneous formation of two nonadjacent spiro-quaternary stereocenters, which requires precise control over both diastereo- and enantioselectivity. Nevertheless, the developed method afforded the desired products in moderate to good yields with good diastereoselectivities and moderate to excellent enantioselectivities of up to 85% ee through a cascade sequence involving Michael addition, keto–imine formation, and intramolecular Friedel–Crafts alkylation. Notably, a dinuclear zinc catalyst in combination with newly designed unsymmetric semi-azacrown ether ligands 227 was employed, enabling the simultaneous activation of both reaction partners and effective control of the stereochemical outcome (Scheme 53).^110^

**Scheme 53:**
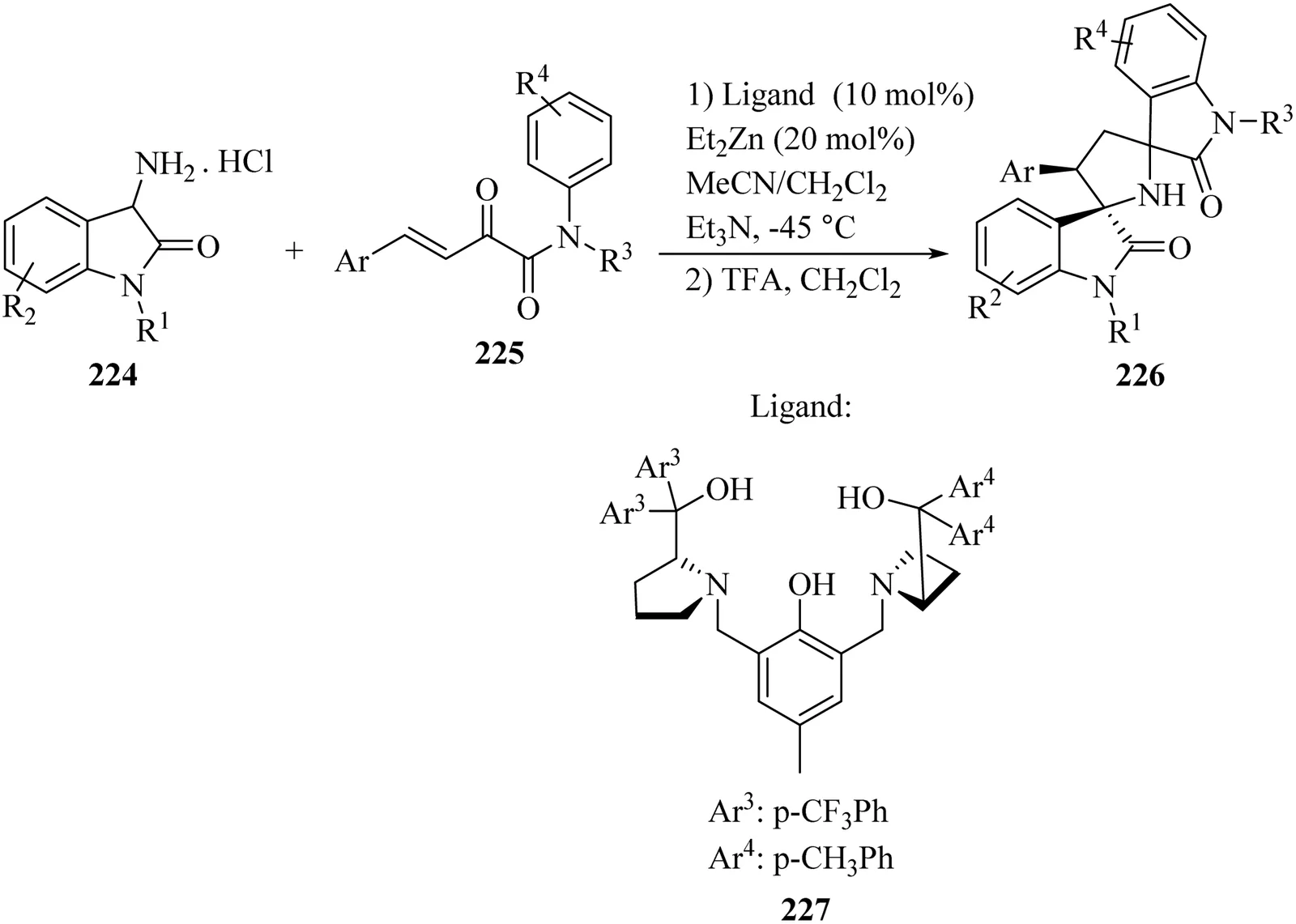
Asymmetric synthesis of 2,5-pyrrolidinyl bisspirooxindoles *via* dinuclear zinc catalyzed asymmetric cascade reactions.

In 2018, Lin's research team described a new synthetic approach for the synthesis of 3,3′-pyrrolidinyl-bisspirooxindoles 228*via* a Michael/*N*-hemiketalization cascade reaction. This strategy employs 3-aminooxindoles which possess two reactive sites 224, making them highly suitable substrates for spirocyclization reactions; in this context, they function as nucleophilic synthons, whereas isatin-derived β,γ-unsaturated α-keto esters 136 serve as highly activated electrophilic alkenes. Under bifunctional squaramide organocatalysis 142, capable of simultaneously activating both the nucleophilic and electrophilic partners through cooperative hydrogen-bonding and Brønsted base interactions, the transformation proceeds with excellent stereochemical control.

This transformation proceeds under mild reaction conditions and enables the construction of bisspirocyclic architectures in moderate to good yields with excellent diastereo- and enantioselectivity. Notably, the methodology allows the simultaneous construction of two spiro stereocenters in a single operation, thereby demonstrating remarkable step economy and synthetic efficiency. It is noteworthy that, this study represents the first reported use of 3-aminooxindoles as nucleophiles in the asymmetric synthesis of 3,3′-pyrrolidinyl-bisspirooxindoles (Scheme 54).^111^

**Scheme 54:**
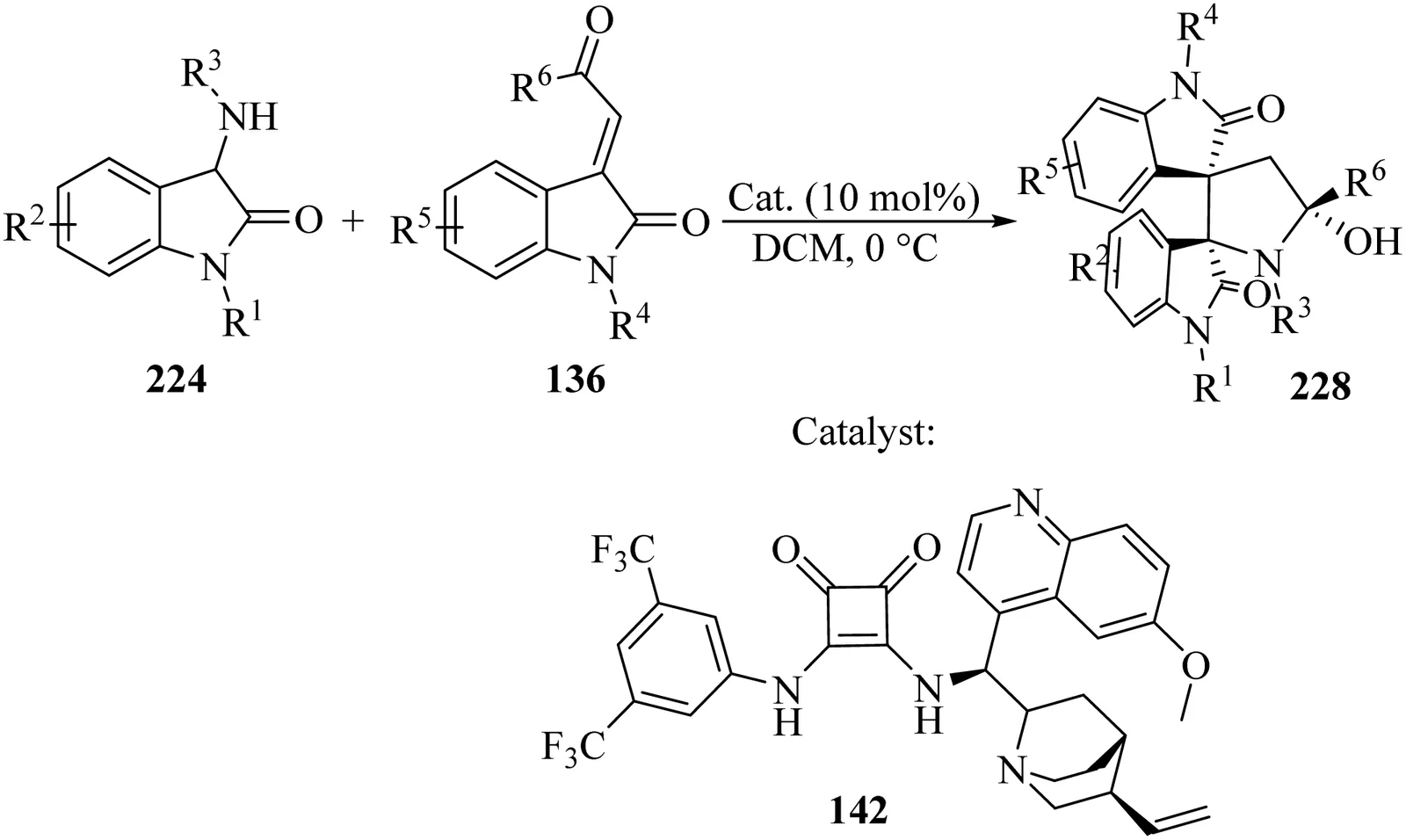
Organocatalytic asymmetric synthesis of 3,3′-pyrrolidinyl bisspirooxindoles.

## 3-Imino-oxindoles-derived bisspirooxindole syntheses

Owing to the presence of an electrophilic imine moiety at the C-3 position, 3-iminooxindoles have emerged as highly versatile synthetic intermediates in spirocyclic chemistry. Their bifunctional nature enables diverse cyclization and annulation reactions, providing efficient access to functionalized bisspirooxindole derivatives. The representative methodologies discussed in this section highlight the synthetic utility of 3-iminooxindoles as valuable precursors for the synthesis of bisspirooxindole frameworks under organocatalytic conditions.

In 2018, Song's^112^ research group worked on the asymmetric synthesis of CF_3_-containing bisspiro[oxindole-pyrrolidine-rhodanine] derivatives 231 using 2-thioxo-1,3-thiazolidin-4-one (rhodanine)^113–115^ derivatives 229 and *N*-(2,2,2-trifluoroethyl)isatin ketimines 230 (Scheme 55). This synthesis was achieved through a highly diastereo- and enantioselective domino Michael/Mannich [3+2] cycloaddition reaction. In this approach, the reaction was carried out in the presence of a squaramide tertiary amine catalyst 232. The final products, which contain four contiguous stereogenic centers including two vicinal spiro-quaternary chiral centers, were obtained with excellent results (up to 99% yield, >99 : 1 dr, and >99% ee). This synthetic route is valuable because spiropyrrolidine^116,117^ frameworks exhibit significant biological activities. Moreover, fluorinated organic compound, particularly those containing the trifluoromethyl (CF_3_) group,^118,119^ constitute important structural motifs in pharmaceutical agents and agrochemicals. Also, 2-thioxo-1,3-thiazolidin-4-one (commonly known as rhodanine) has attracted considerable attention owing to its diverse medicinal and pharmacological activities.

**Scheme 55:**
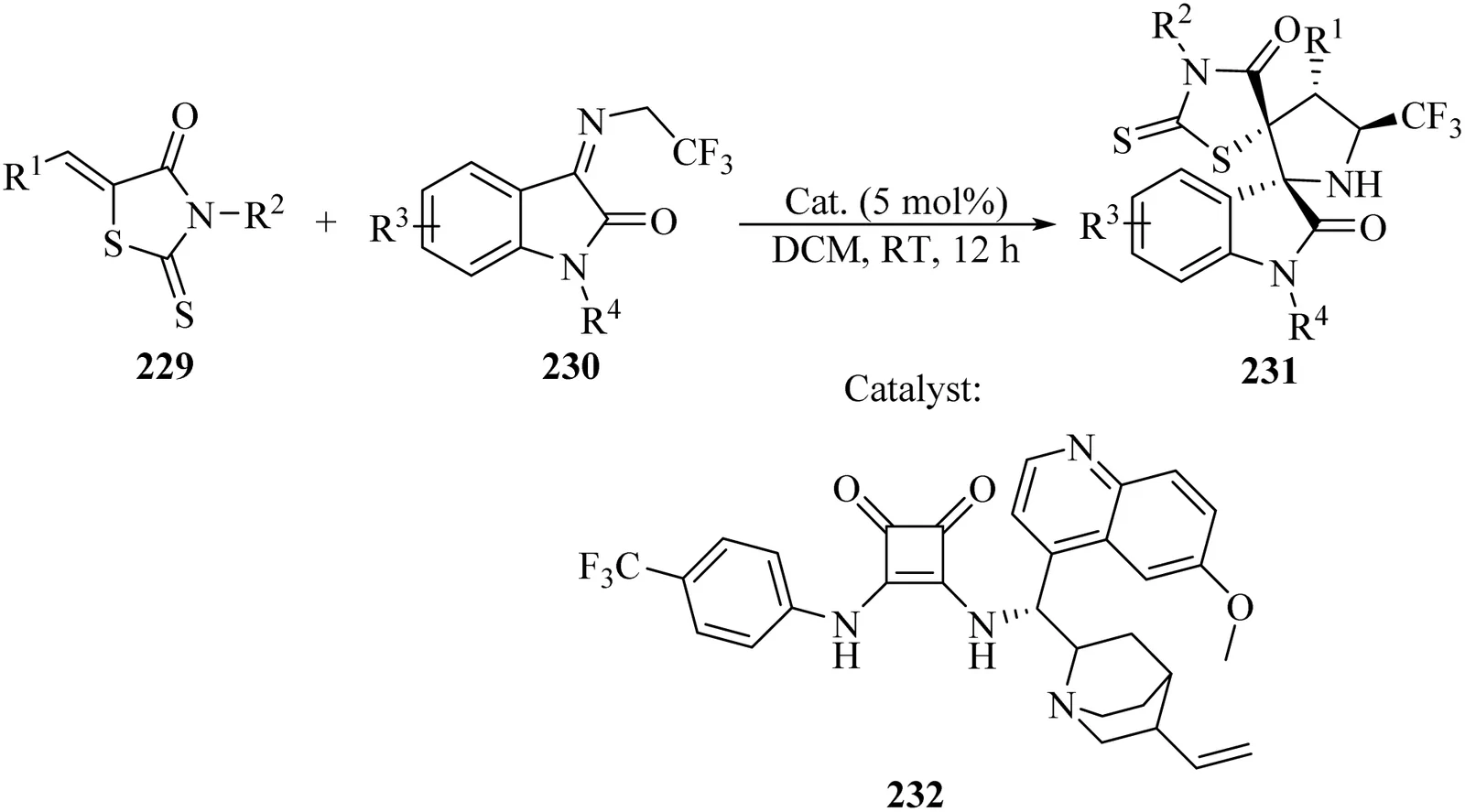
Squaramide-catalyzed asymmetric synthesis of bisspiro[oxindole-pyrrolidine rhodanine]s.

Zhao and Du presented a Mannich/cyclization cascade reaction between isatinimines 233 and isothiocyanates derived from 1-indanones 234. This catalytic heteroannulation, which employs a squaramide catalyst 135, enables the construction of bisspirocyclic indanone-thioimidazolidine-oxindoles 235 bearing two adjacent spiro-quaternary stereocenters in good to excellent yields with excellent diastereo- and enantioselectivities. In addition, this method showed that the final products can be transformed into other bioactive heterocyclic compounds with medicinal and pharmaceutical properties (Scheme 56).^120^

**Scheme 56:**
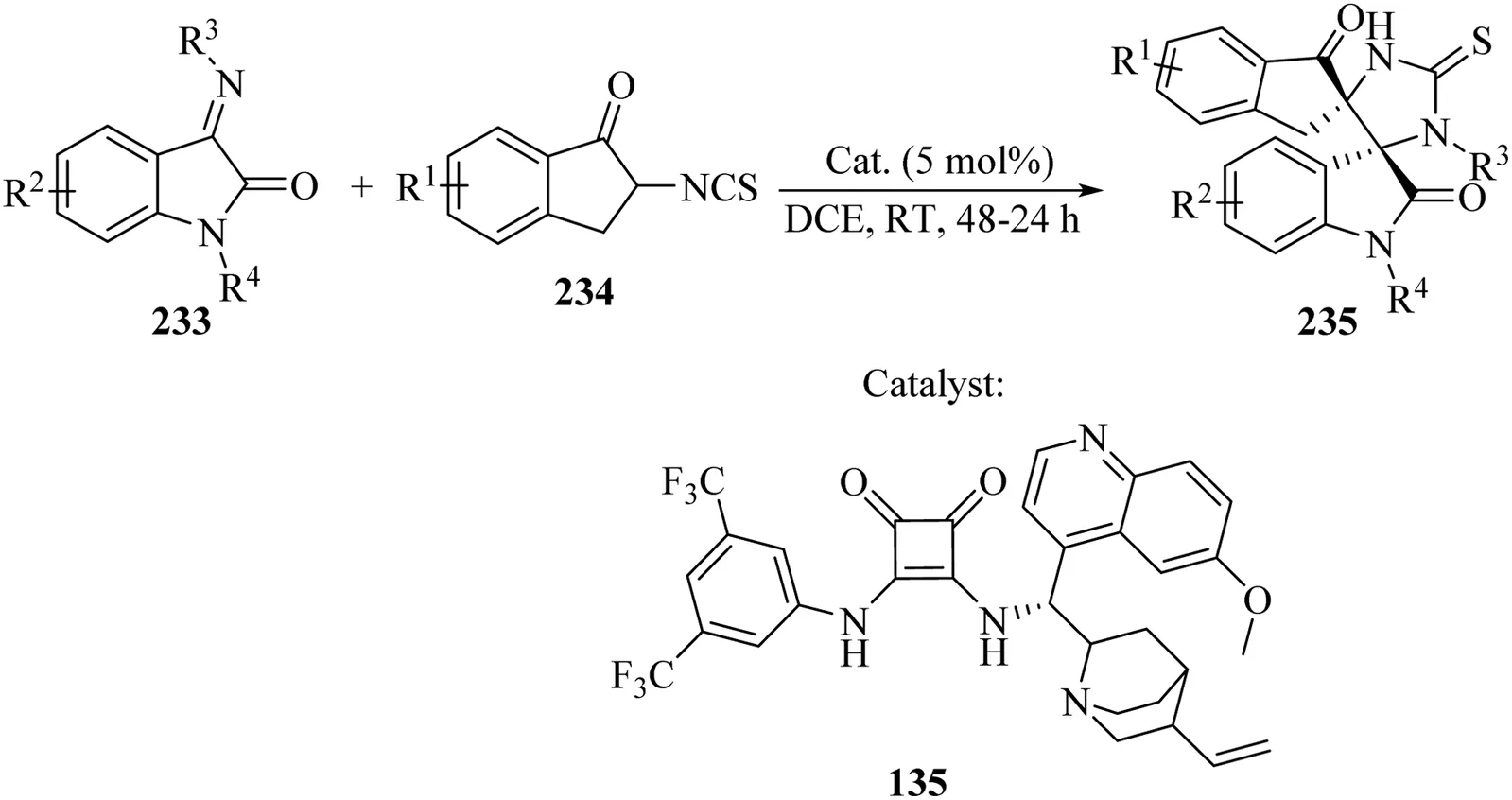
Catalytic asymmetric strategy for the synthesis of bisspirocyclic indanone–thioimidazolidine–oxindoles.

Zhao *et al.* disclosed an asymmetric synthesis of 3,3′-pyrrolidinyl-bisspirooxindoles 238 in 2016.^121^ They used a reaction between *N*-Boc-protected isatin-derived ketimines 236,^122–129^ and donor–Michael acceptor synthons 237,^130,131^ through an organocatalytic domino one-pot sequence involving of Mannich/Boc-deprotection/aza-Michael reactions to obtain the desired products. Using the commercially available (DHQD)_2_PHAL catalyst 239, this method efficiently generated products containing three stereogenic centers, including two vicinal spiro-stereocenters (Scheme 57). The desired compounds were obtained in good yields with excellent diastereo- and enantioselectivities, and the reaction could be readily scaled up.

**Scheme 57:**
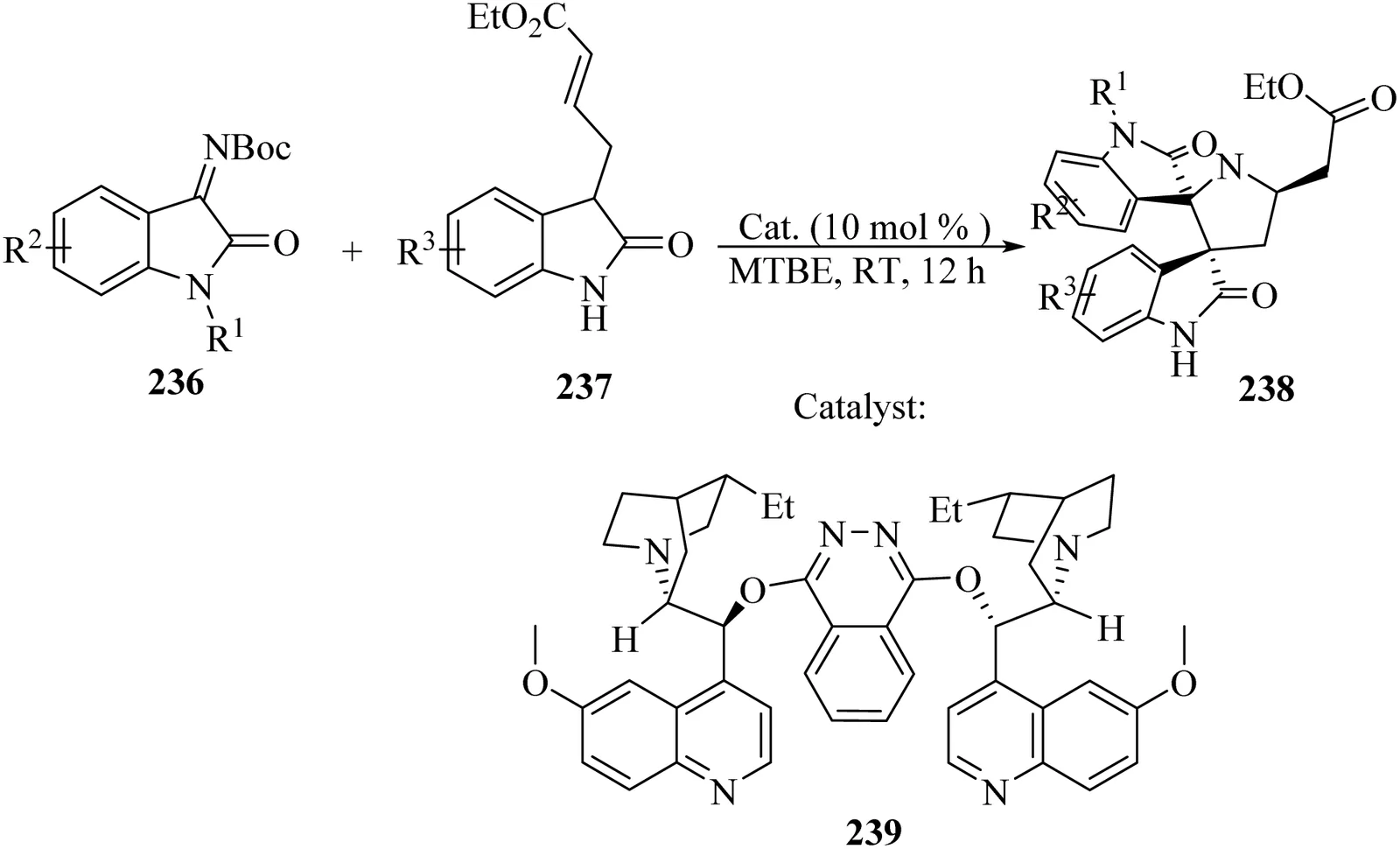
Stereoselective synthesis of functionalized 3,3′ pyrrolidinyl-bisspirooxindole derivatives.

## Transition-metal catalyst spirovinylcyclopropyl oxindoles transformation to bisspirooxindole

The synthetic approaches discussed in this category employ spirovinylcyclopropyl oxindoles as key starting materials for the construction of bisspirooxindole frameworks. Owing to their inherent ring strain and distinctive reactivity, spirovinylcyclopropyl oxindoles can undergo ring–opening reactions and followed by cyclization and annulation reactions with various reaction partners, enabling the efficient construction of structurally diverse bisspirooxindole cores through transition-metal catalyst approach.

In 2023, Debnath's research team reported a palladium-catalyzed (3+2) annulative C–C coupling reaction between *para*-quinone methides (*p*-QMs) 240 and spirovinylcyclopropyl oxindoles (SVCP) 241, enabling the efficient construction of bisspirooxindole frameworks 242. This methodology features mild reaction conditions, high diastereoselectivity, broad functional group tolerance, and is further supported by mechanistic insights derived from DFT calculations (Scheme 58).^132^

**Scheme 58:**
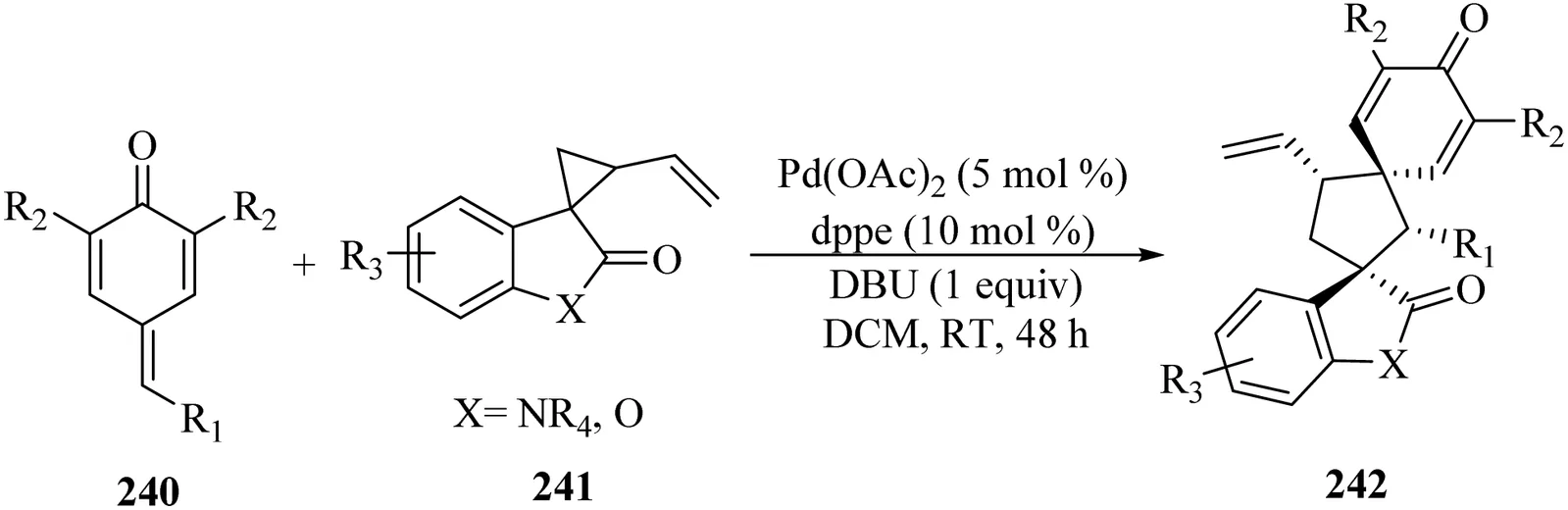
[3+2] cyclization of SVCP and *p*-QM.

In 2022, Zhang, Miao, and colleagues proposed a ligand-controlled strategy for the synthesis of heterocyclic compounds. Based on a palladium-catalyzed asymmetric annulation between spirovinylcyclopropyl oxindoles 243 and o-quinone methides 244, they employed different chiral ligands to selectively access two distinct classes of products with excellent control over both regioselectivity and stereoselectivity.^133^

In the first pathway (Scheme 59, Path A), the use of the chiral ligand Segphos 245 in a [4+3] cyclization reaction, produced spirooxindole-benzo[*b*]oxepine derivatives 246 in good yields (up to 78%) and excellent enantioselectivities (up to 98% ee).

**Scheme 59:**
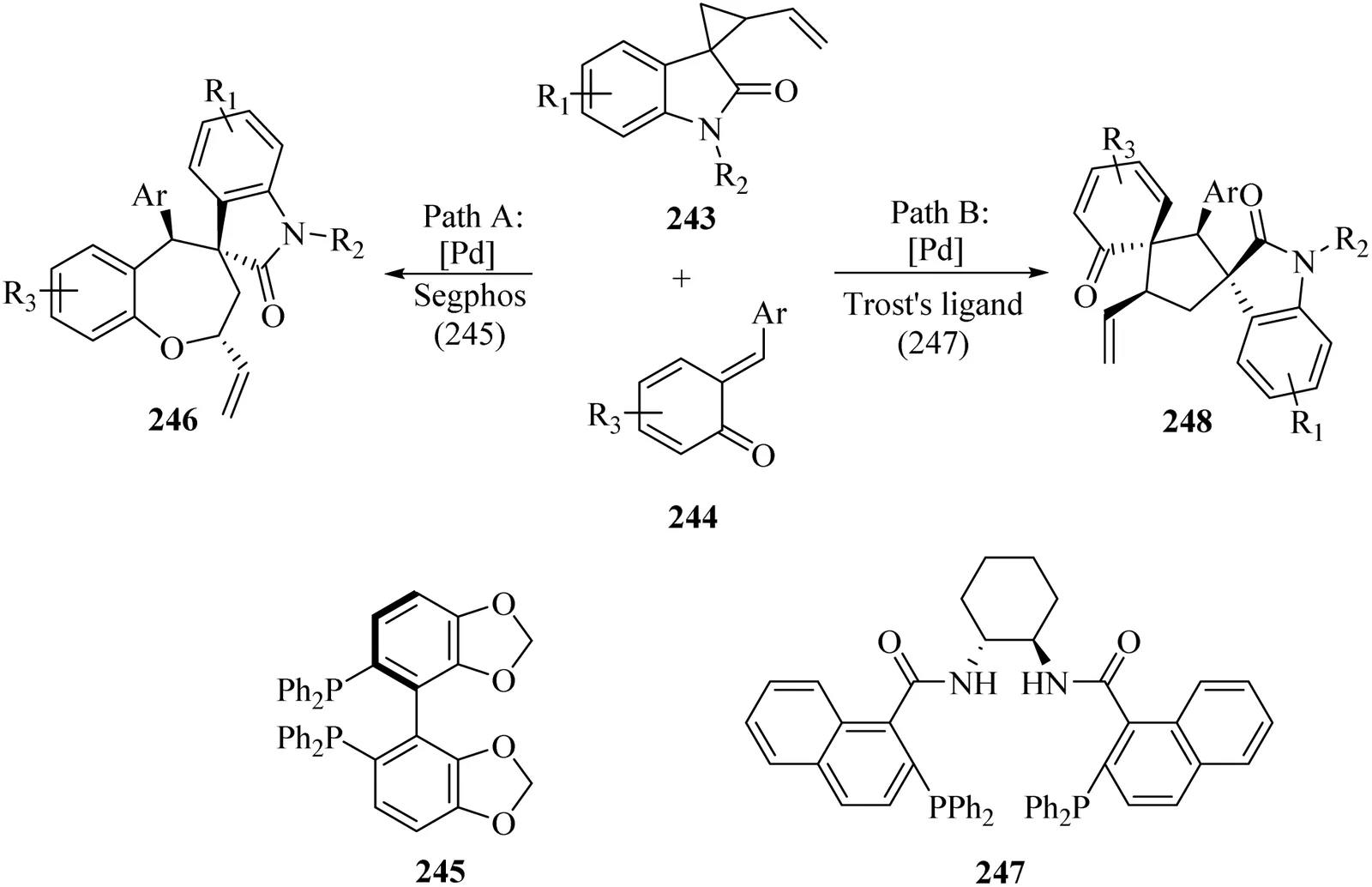
Palladium-catalyzed asymmetric sterategy for the synthesis of spirooxindole-benzo[*b*]oxepine derivatives.

In the second pathway (Scheme 59, Path B), Trost's ligand 247 was employed in a [2+3] cyclization reaction, affording bisspirooxindole derivatives 248 with good yields (up to 77%) and enantioselectivities up to 99% ee.

## Aniline-driven access to bisspirooxindole scaffolds

This category encompasses synthetic methods in which aniline derivatives serve as key starting materials for the construction of bisspirooxindole derivatives. The nucleophilic character of the amino group and the ability to participate in C–H and C–X (X = Br or I) activation and cyclization processes make aniline derivatives valuable building blocks for the development of diverse spirooxindole-forming strategies. The four studies discussed in this section illustrate the versatility of transition-metal catalyzed, aniline-based strategies for the synthesis of bisspirooxindole frameworks.

In 2022, Zhou reported a novel chemoselective decarboxylative annulation reaction between different aryl iodides 249–251 and cyclic β-bromoacrylic acids 252, which act as versatile C-1 or C-2 synthons and represent a new concept in annulation chemistry. This transformation proceeds *via* a Pd(0)/Pd(ii) catalytic cycle, in which a range of aryl iodides undergo oxidative addition in the presence of a palladium catalyst, leading to the formation of key palladacycle intermediates.

Also, cyclic β-bromoacrylic acids undergo decarboxylation under palladium catalysis, generating reactive vinyl–Pd or π–allyl-type intermediates. Notably, three distinct classes of aryl iodides, including 2-iodo-*N*-methacryloylbenzamides 249, 2-iodobiphenyls 250, and *N*-(2-iodophenyl)-2-phenylacrylamides 251, were successfully employed, enabling the selective construction of fused and spiro polycyclic frameworks 253–255 (Scheme 60).^134^ These polycyclic compounds, such as hexahydrodibenzoisoquinoline diones 253, spirofluorenes 254, and bisspirooxindoles 255, are important structural motifs that are widely found in bioactive molecules and functional materials.^135–137^

**Scheme 60:**
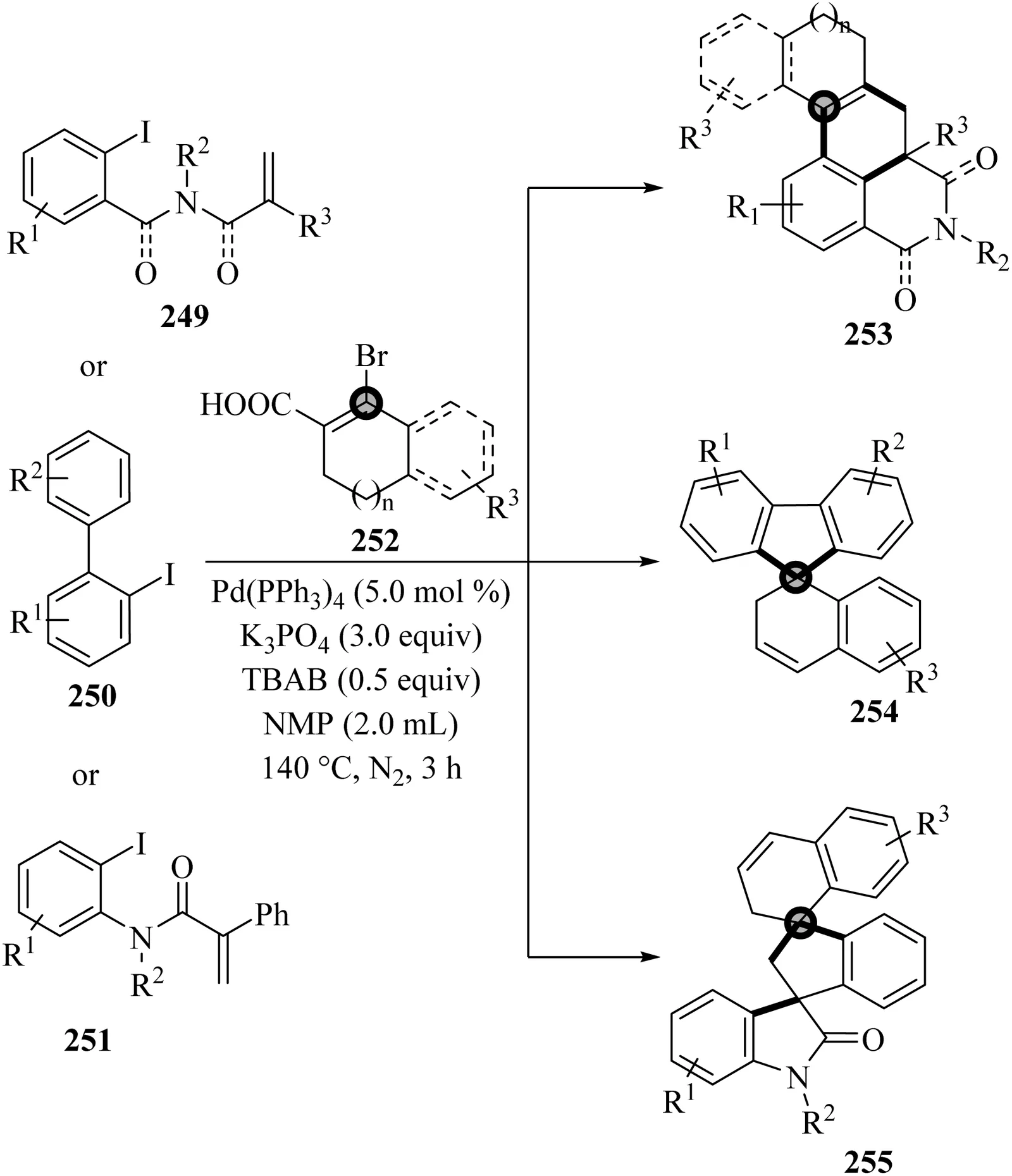
Chemoselective construction of fused or spiro polycycles.

In 2019, an efficient asymmetric approach was developed by Liu for the synthesis of bisspirotetrahydrofuranoxindoles derivatives 258 containing three contiguous stereogenic centers, including two spiro-quaternary chiral centers, with excellent diastereo- and enantioselectivities under mild conditions. The reaction proceeds through a bimetallic cooperative catalytic cascade involving Michael addition, hemiketalization, and Friedel–Crafts reaction between β,γ-unsaturated α-ketoamides 256 and 2-hydroxy-1-indanones 257. In this strategy, a dinuclear zinc–AzePhenol catalyst 259 was employed, in which the zinc centers play a dual role: one site activates the electrophilic and nucleophilic reaction partners, while the other controls stereochemistry. The reaction can be performed on a gram scale using only 2 mol% catalyst. Notably, ongoing studies are focused on evaluating the biological properties of these compounds (Scheme 61).^138^

**Scheme 61:**
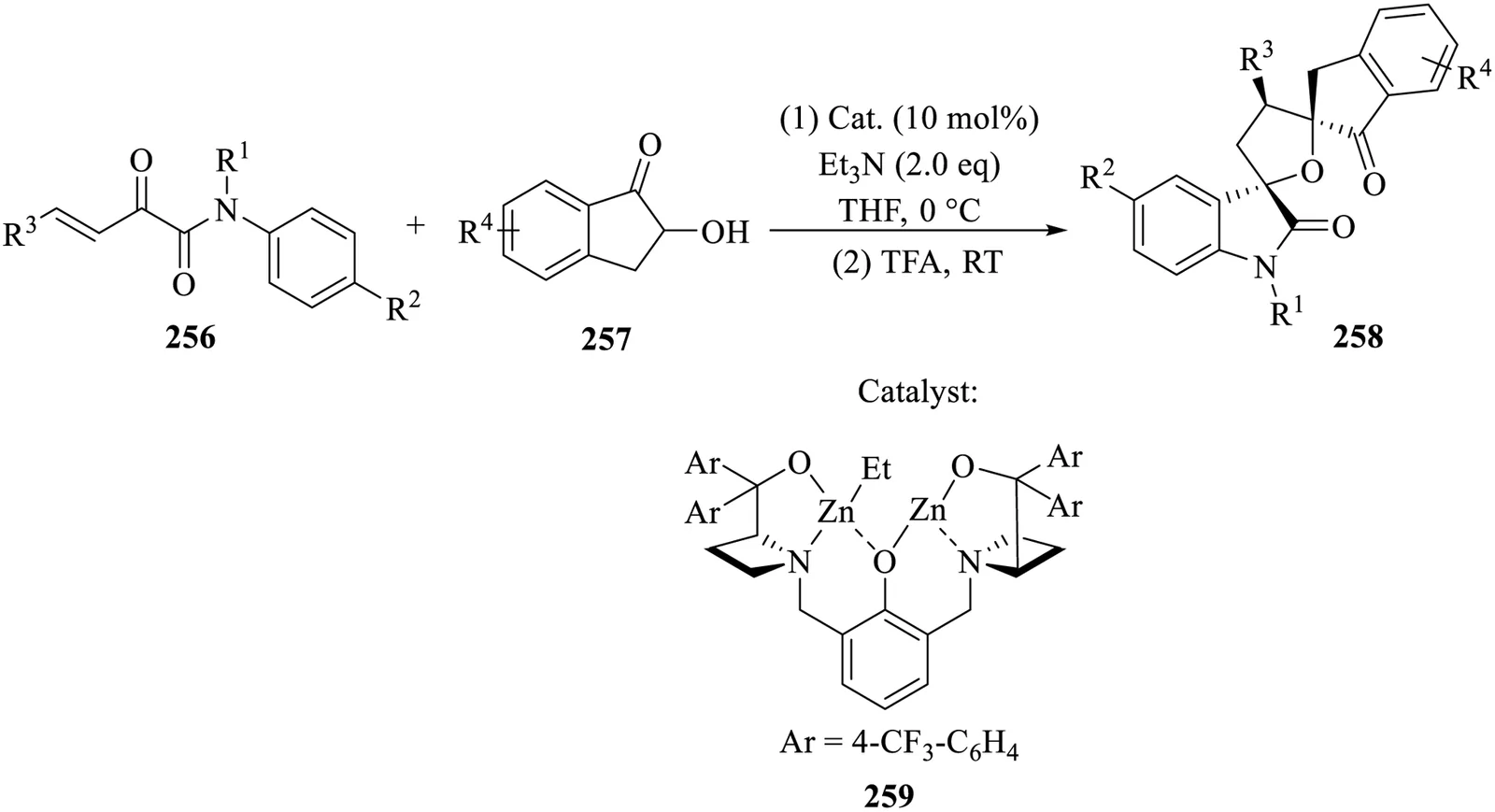
Access to chiral bispirotetrahydrofuranoxindoles.

In 2016, Liu *et al.* described a palladium-catalyzed arylative dearomatization/ring expansion cascade of furfurylcyclobutanols 260 to provide stereochemically defined bisspirooxindoles 261 (Scheme 62).^139^ The reaction proceeds *via* a spiro π–allyl palladium intermediate, enabling the formation of two quaternary carbon centers in a single step with excellent diastereospecificity and enantioselectivity. Furfuryl cyclobutanols bearing a 1-hydroxycyclobutyl group 260 serve as the starting materials, and the reaction is promoted by Pd_2_(dba)_3_ in the presence of bis[2-(diphenylphosphino)phenyl]ether 262 and K_2_CO_3_ in THF at 100 °C. This approach combines high step economy with structural complexity and enables the efficient construction of novel bisspirooxindoles that are otherwise challenging to access through conventional synthetic methodologies. Mechanistically, the formation of the spiro π–allyl palladium intermediate followed by ring expansion is key to achieving both diastereo- and enantioselectivity, representing the first report of this strategy for constructing bisspiro compounds. The resulting scaffolds are not only synthetically valuable but also biologically interesting, and further studies aimed at expanding the reaction scope are currently in progress.

**Scheme 62:**
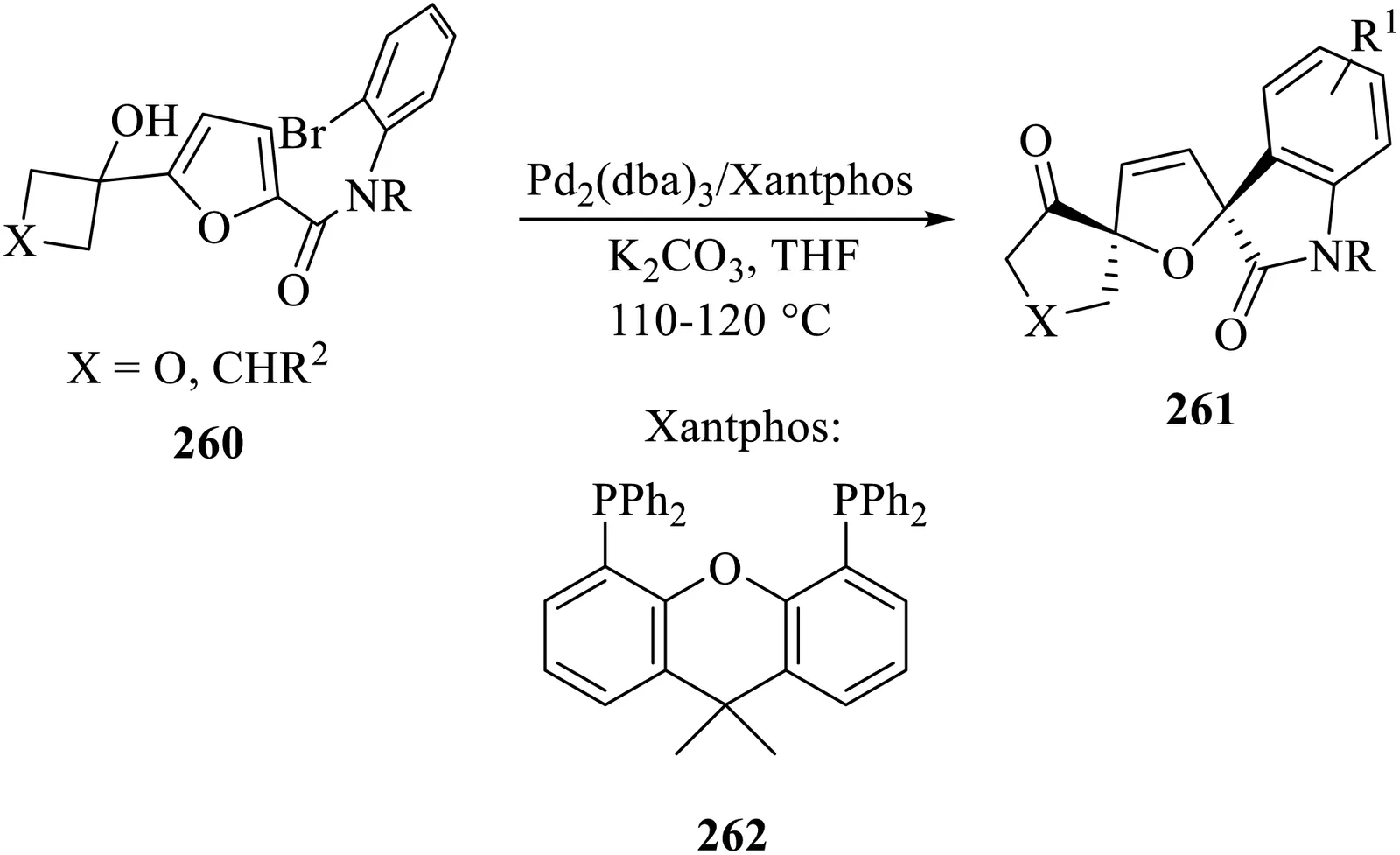
Pd-catalyzed cascade reaction for the synthesis of bisspirooxindoles.

Drouhin and co-workers reported in 2014 an efficient, one-pot, copper(ii)-mediated double C–H/Ar–H coupling strategy for the construction of structurally diverse spirocyclic bis-oxindole frameworks 264 using readily available bis-anilide precursors 263. This oxidative cyclization allows the diastereoselective formation of two all-carbon quaternary centers at the C-3 positions of the oxindole units in a single step (Scheme 63).^140^ The reaction was inspired by earlier Cu-mediated oxidative cyclizations developed by the Perry and Moody groups for the synthesis of oxindoles,^141–144^ and related heterocycles.^145,146^ Importantly, this method provides flexibility in the central linker unit, enabling access to various bisspirooxindole structures with increased molecular complexity.

**Scheme 63:**
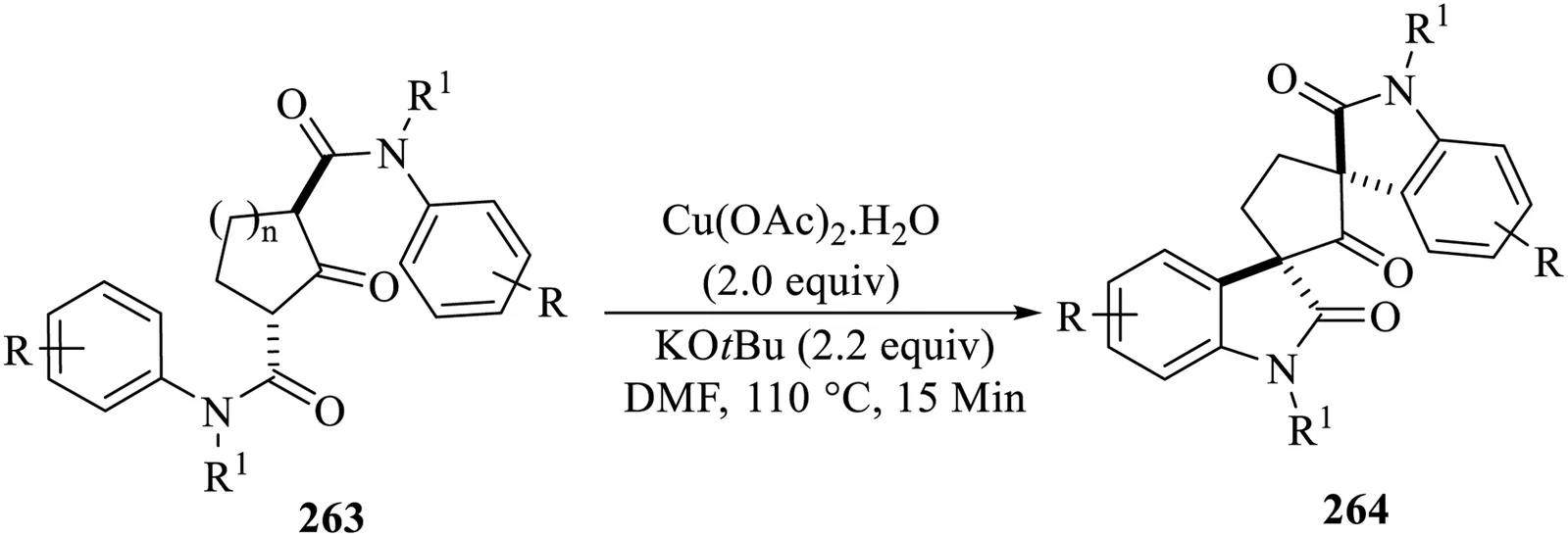
Double C–H, Ar–H coupling process for the synthesis of spirocyclicbis-oxindoles.

## Unconventional synthetic approaches to bisspirooxindole

Despite the broad range of bisspirooxindole syntheses that can be classified according to their key starting materials, a number of valuable methodologies do not fit conveniently within the categories discussed above. Two different studies are described in this section. In one study, diazooxindoles were employed as the starting materials, whereas in the other study a functionalized indole was utilized as the starting material. The first study reports a rhodium-catalyst approach to bisspirooxindole synthesis, whereas the second is based on a rearrangement strategy involving the conversion of an indole to a spirooxindole.

Recently, the Rh-catalyzed reaction of diazooxindoles 265 and thiolactones 266 has been reported for the synthesis of bisspirooxindole fused S-heterocycles 267–269 (Scheme 64).^147^ The use of diazooxindole derivatives and δ-thiolactone as starting materials in the presence of Rh_2_(OAc)_4_, enables the insertion of two diazooxindole units into the δ-thiolactone through a formal [1,3]-acyl migration reaction, providing medium-sized adjacent bisspirooxindole scaffolds 268 in excellent yields and with high levels of chemo- and diastereoselectivity.

**Scheme 64:**
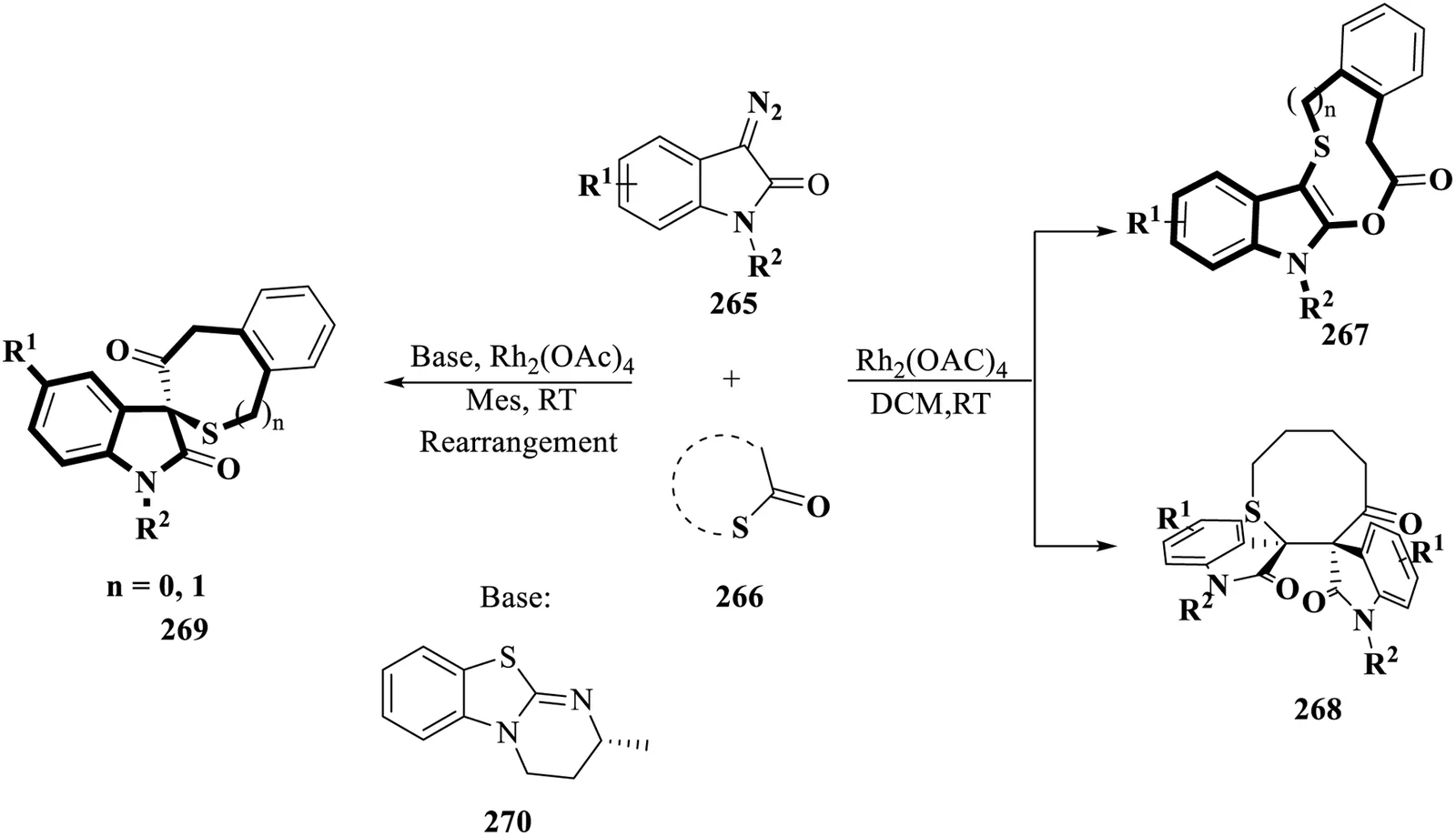
An efficient route to diverse indole/oxindole-annulated S-heterocycles.

Citrinadin A,^148,149^ a natural product isolated from a marine-derived fungus, has garnered significant interest from the synthetic chemistry community due to its intricate and architecturally challenging molecular structure. It has nine stereocenters, an R-epoxycarbonyl group, and a rare *N*,*N*-dimethylamino valine residue. The central five-membered ring is densely substituted, with almost all the carbon atoms fully substituted, including two adjacent quaternary centers. On top of that, this compound shows biological activity, being cytotoxic against murine leukemia and human epidermoid carcinoma cells.

In 2007, Martin and colleagues reported an efficient, stereoselective approach to the challenging synthesis of the spirooxindole moiety of citrinadin A based on an oxidative rearrangement. Dimethyldioxirane (DMDO) was used to oxidize indole 271 to oxindole 273 while an 8-phenylmenthol chiral auxiliary was attached to the indole nitrogen. This method enabled the construction of two adjacent quaternary centers in good yield and with high stereoselectivity, and importantly, it was the first example of an enantioselective oxidative rearrangement of an indole to a spirooxindole (Scheme 65).^150^

**Scheme 65:**
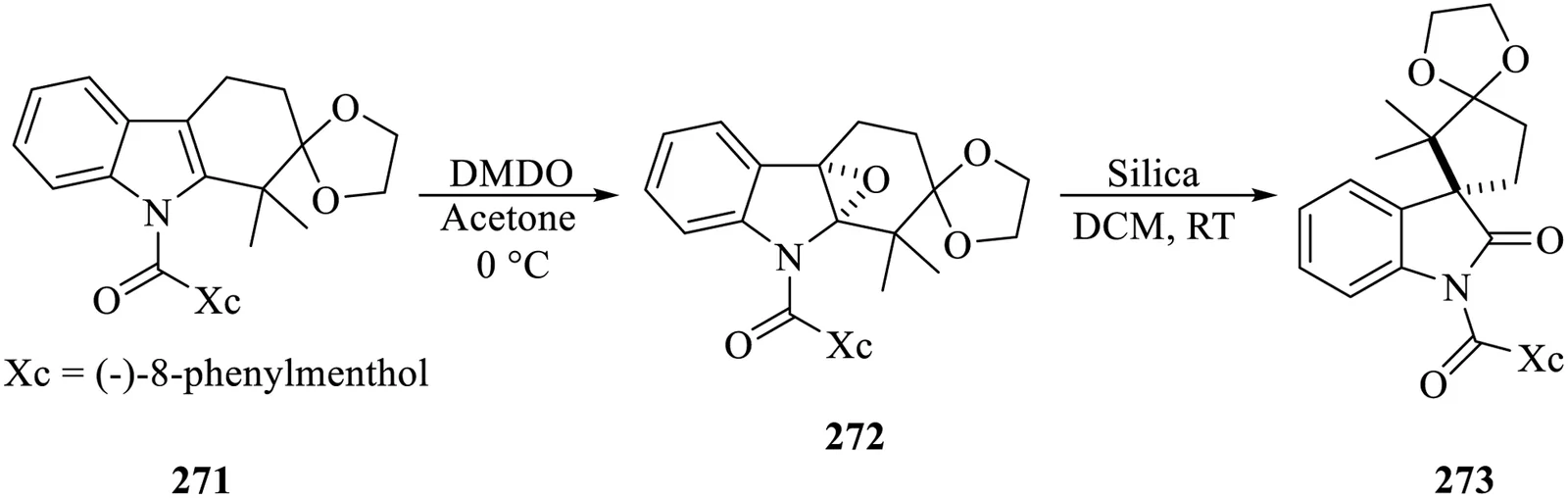
Stereoselective synthesis of spirooxindole ring system of citrinadin A.

## Synthesis of bisspirooxindole structure connected by a linker

The formation of molecular frameworks containing two spiroindole moieties linked through a spacer or bridging moiety has attracted considerable attention from both synthetic and biological perspectives. Due to their structural diversity achievable through variation of both the spirooxindole units and the connecting linker, various synthetic methods have been developed for their preparation. This section summarizes different synthetic strategies for the preparation of bisspirooxindole scaffolds, highlighting key reaction designs, linker diversity, substrate scope, and potential applications.

Wagh and coworkers, in 2019, developed a CsF-promoted, one-pot multicomponent strategy for the synthesis of spirooxindole-pyran derivatives 276. Following the successful synthesis of mono-spiro compounds, the methodology was subsequently extended to the construction of bisspirooxindole-pyran derivatives *via* the reaction of isatins or bis-isatins 274 with malononitrile 86 and various C–H-activated compounds 275, providing the corresponding products in excellent yields and with notably short reaction times (Scheme 66).^151^

**Scheme 66:**
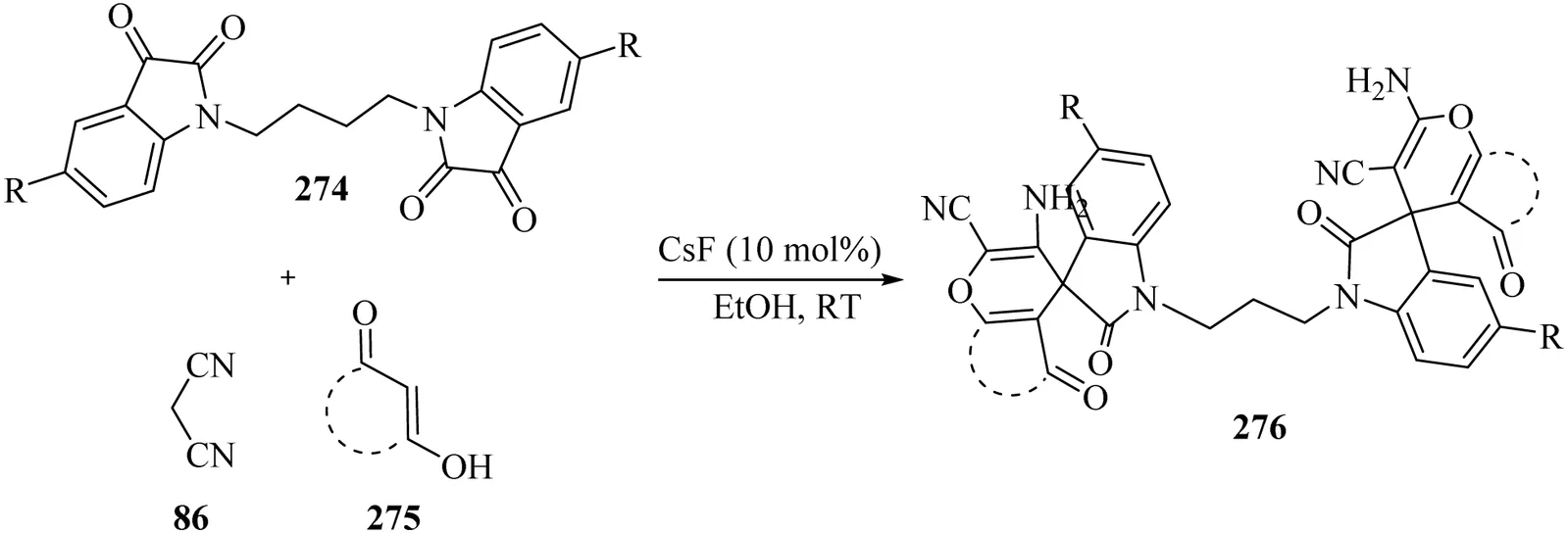
Multicomponent access to bisspirooxindole-pyran derivatives.

Notably, the products were purified by simple filtration without chromatographic purification. Bisspiro frameworks are of considerable interest, as introducing different substituents on the two heterocyclic rings can lead to enhanced biological activity.

In 2018, Hasaninejad and Beyrati reported an efficient and environmentally friendly strategy for the synthesis of asymmetrical bisspirooxindole derivatives 281*via* a one-pot, two-step, four-component reaction involving *N*-alkyl isatin 277, isatin derivatives 78, alkyl malonates 279, and C–H-activated carbonyl compounds 280. The reaction was carried out at room temperature in the presence of polyethylene glycol 400 (PEG-400) as a green, biodegradable, and eco-friendly reaction medium, while potassium carbonate (K_2_CO_3_) was used as a mild base catalyst. The desired products were obtained in good to excellent yields (Scheme 67).^152^

**Scheme 67:**
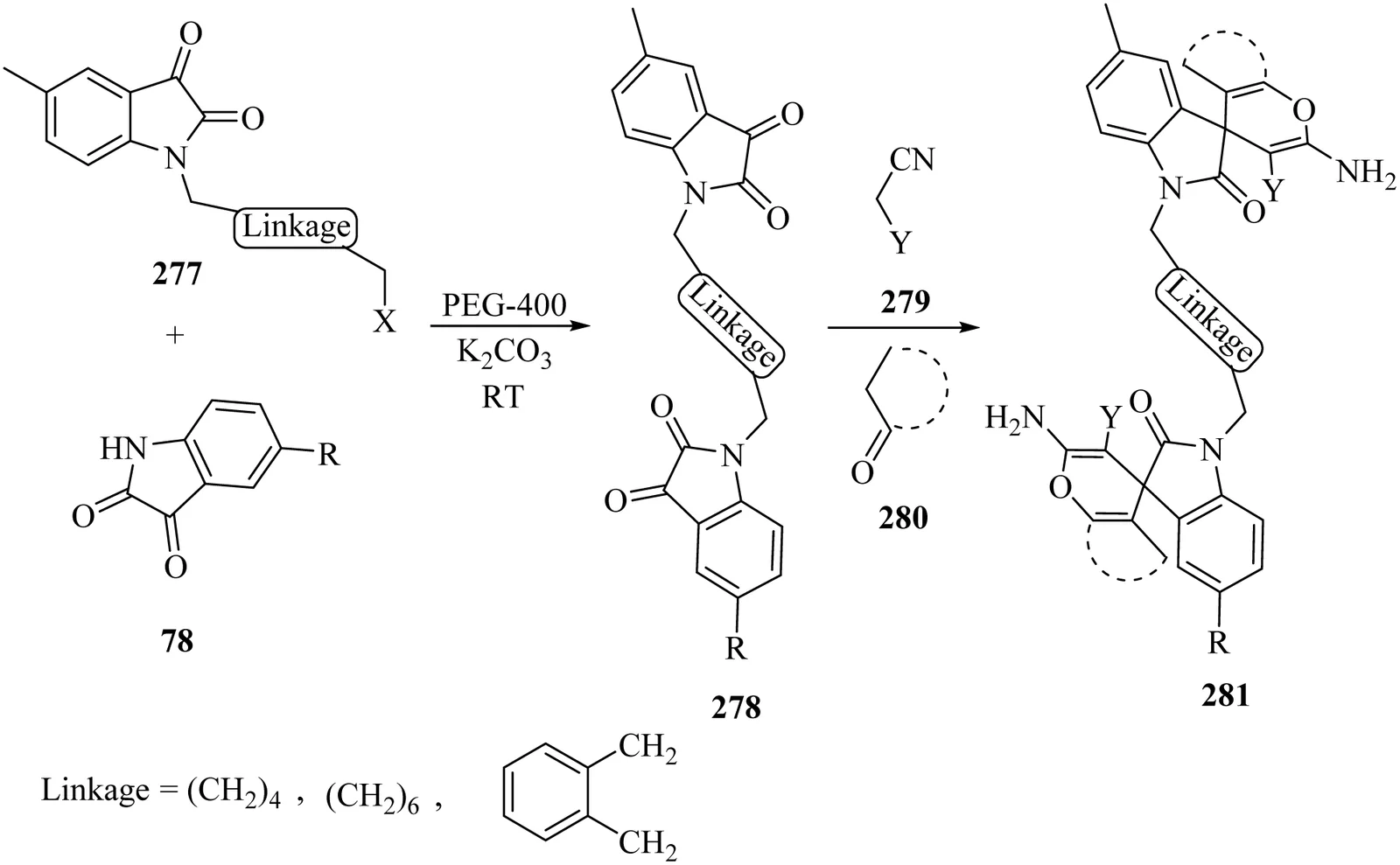
A straightforward approach to the synthesis of asymmetrical bisspirooxindole derivatives.

According to the World Health Organization (WHO), infectious diseases continue to represent a major global cause of mortality.^153^ The effective treatment of these diseases has become progressively more difficult as a consequence of the ongoing emergence of drug-resistant bacterial strains.^154^ Consequently, the design and development of new medicinal compounds is considered an important and urgent objective in medicinal chemistry. In this context, bisspirooxindoles, dispirooxindoles, and bisspiroindole frameworks have attracted considerable attention as promising scaffolds for the development of efficient antimicrobial agents.

Syumka *et al.*^155^ described an efficient method for the synthesis of ethylene-linked bisspirooxindole derivatives containing a pyrrolo[3,4-*c*]pyrrole core *via* a [3+2] cycloaddition reaction of azomethine ylides generated *in situ* from isatins and amino acids. The reaction employed *N*,*N*′-di(3-carboxypropenoyl)-1,2-ethylenediamine as the dipolarophile, a compound structurally related to the antibacterial drug ethambutol.^156^ Biological evaluation revealed significant antimicrobial activity against several Gram-positive and Gram-negative bacterial strains, as well as antifungal activity against *Candida albicans*.

Multicomponent reactions (MCRs) are suitable and efficient methods for synthesizing heterocyclic compounds due to their rapid synthesis, atom economy, and selectivity.^157–164^ These strategies are also appropriate for generating complex structures such as bis-heterocyclic compounds.^165–168^^.^

In a study reported by Ghozlan in 2017, bis(indoline-2,3-diones) 282 were used as versatile precursors in one-pot three-component reactions with malononitrile 86 and 1,3-dimethylbarbituric acid 283 or dimedone 284. The method led to the formation of bis(spirooxindoles) containing pyrano[2,3-*d*]pyrimidine-6-carbonitrile 285 and 4*H*-chromene-3-carbonitrile 286 frameworks in good to excellent yields. Moreover, the advantages of this approach include readily available reactants, a simple procedure, and structurally diverse products (Scheme 68).^169^

**Scheme 68:**
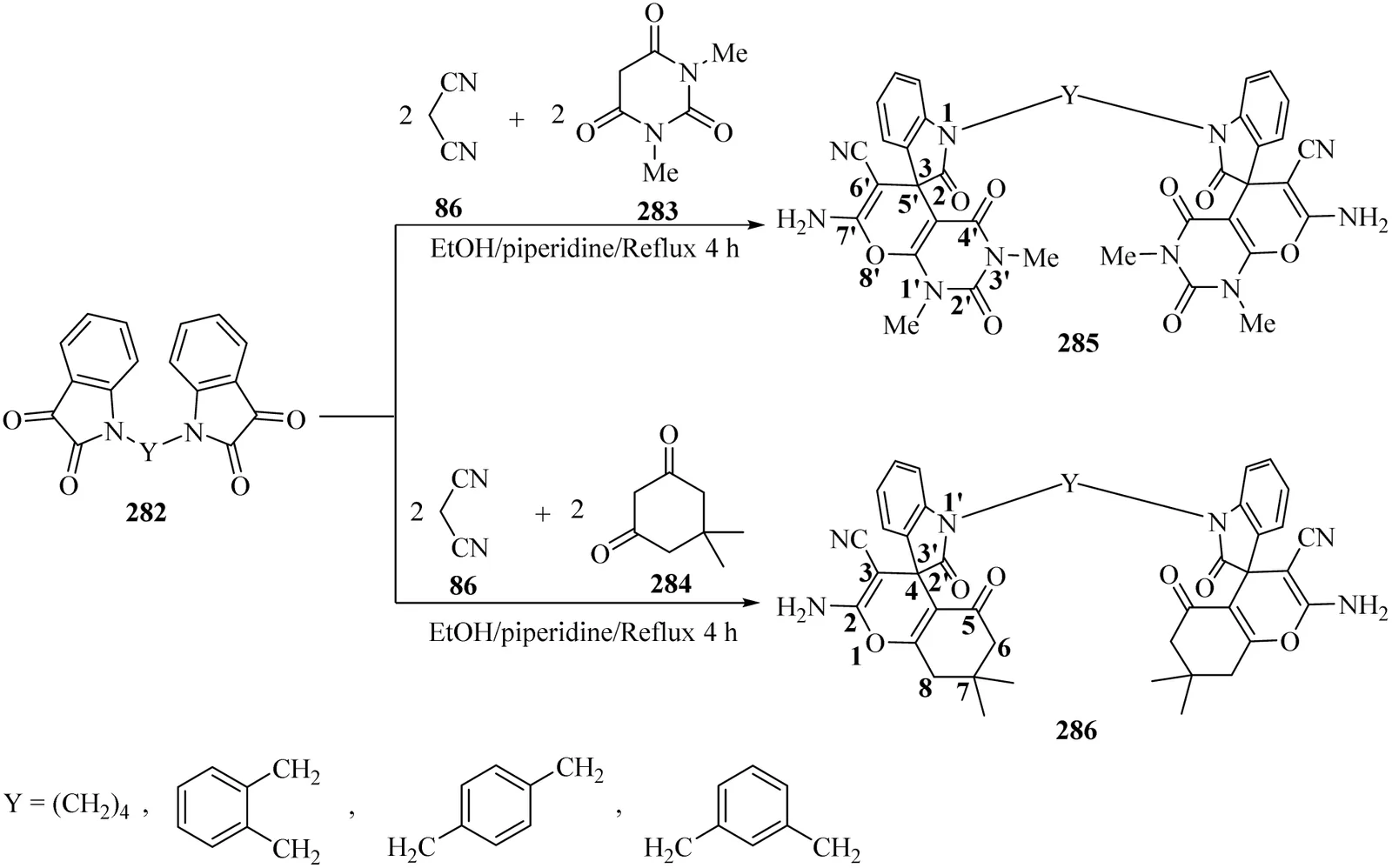
Synthesis of bisspirooxindoles containing 4*H*-chromene-3-carbonitrile and pyrano[2,3-*d*]pyrimidine-6-carbonitrile frameworks.

A three-component and facile synthetic protocol was developed by Sun *et al.* in 2012, for the synthesis of complex bisspirooxindole-fused heterocycles. The synthetic protocol is based on a domino 1,4-dipolar addition and Diels–Alder cycloaddition sequence utilizing Huisgen 1,4-dipoles generated *in situ* through the reaction of 4-dimethylaminopyridine 287 and acetylenedicarboxylate 288 with 3-phenacylideneoxindoles 136. The operationally simple protocol, combined with short reaction times and readily accessible substrates (DMAP, acetylenedicarboxylates, and 3-phenacylideneoxindoles), renders this domino transformation a valuable tool for the construction of structurally diverse heterocyclic frameworks 289. The synthetic utility of this methodology may prove significant in organic synthesis and medicinal chemistry (Scheme 69).^170^

**Scheme 69:**
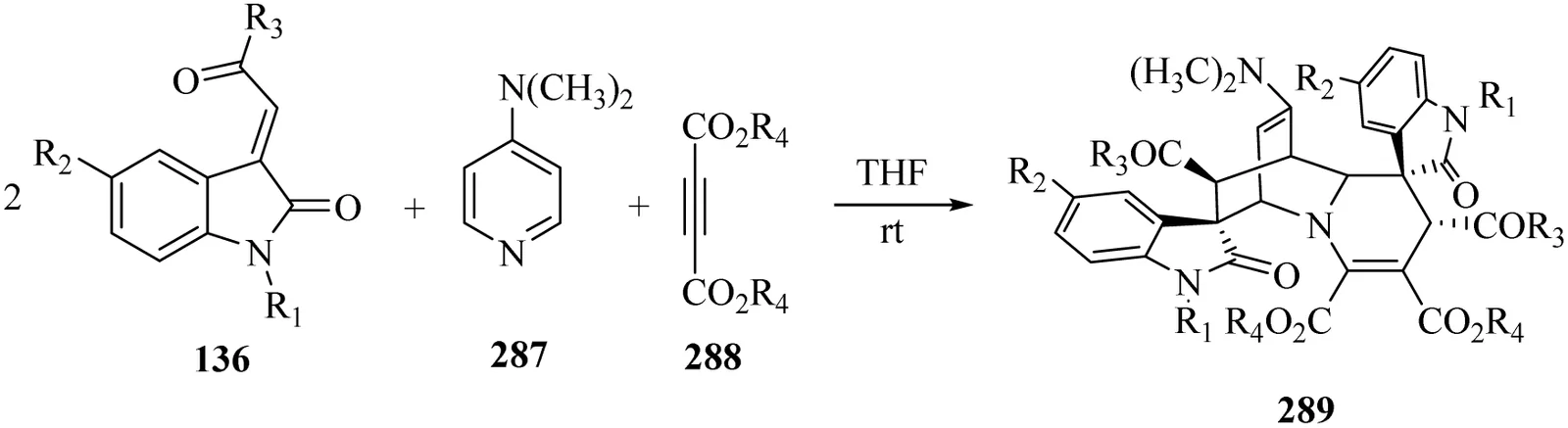
An efficient route to complex bisspirooxindole-fused heterocycles.

A sequential multi-component reaction of isatins, dihalides, malono derivatives 279 and C–H activated carbonyl compounds 291 or ketene aminal derivatives 293 was developed by Safari's research group for the synthesis of novel symmetrical bisspirooxindole derivatives (Scheme 70).^171^ Among the key advantages of this protocol are the use of PEG-400 as a biodegradable solvent, short reaction times, and the easy separation and purification of the desired products. Another notable strength of this strategy is its broad substrate scope, which enables the synthesis of structurally diverse bisspirooxindole derivatives, including bisspiro-indoline-pyranopyranes, bisspiroindoline-chromenes, bisspiro-indoline-pyrido-pyrimidines, bisspiro-indoline-imidazo-pyridines and bisspiro-indoline-pyridines.

**Scheme 70:**
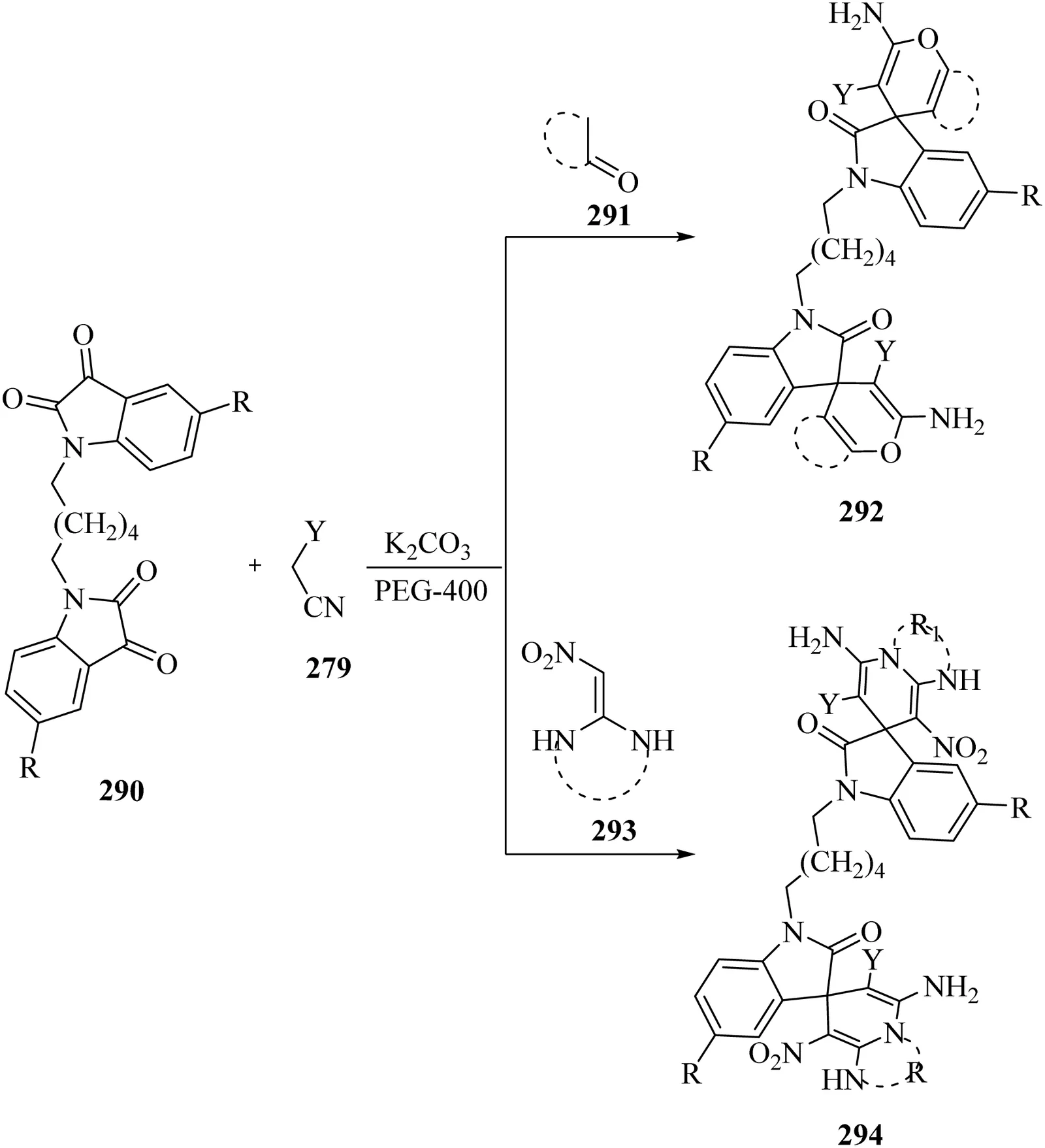
A versatile strategy for the synthesis of bis spirooxindoles with skeletal diversity *via* sequential multi-component reaction in PEG-400 as a biodegradable solvent.

The synthesis of mono- and bisspiro-2-amino-4H-pyrans through a multicomponent reaction using a heterogeneous catalyst was reported by Karimi *et al.* in 2014. In this methodology, the desired products were synthesized in good yields using S-alkyl O-hydrogen sulfothioate functionalized silica-coated magnetic nanoparticles 300 as a heterogeneous catalyst under ultrasound irradiation. This catalyst is of considerable interest due to its convenient handling and efficient recovery *via* magnetic separation (Scheme 71).^172^

**Scheme 71:**
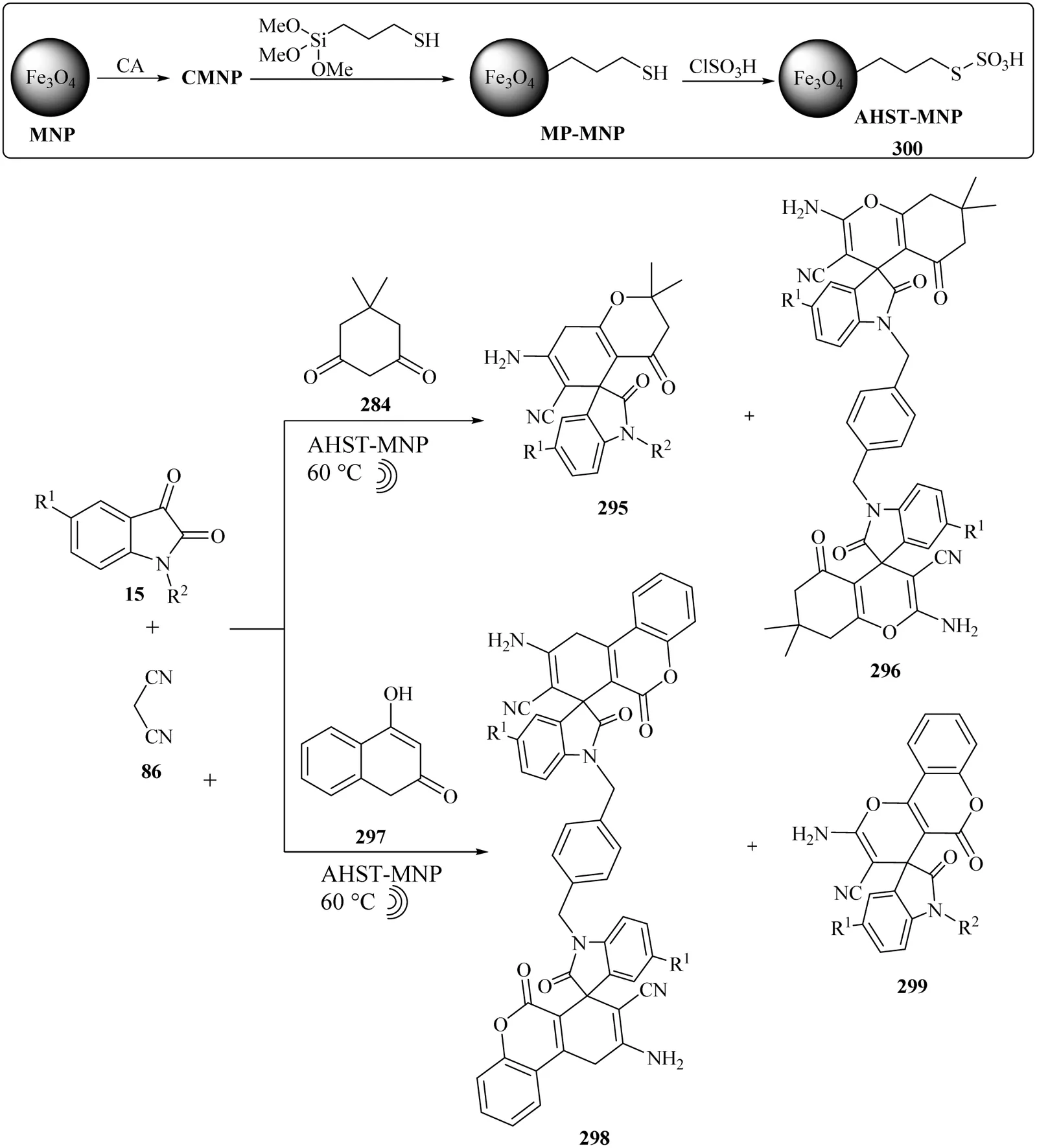
An efficient heterogeneous catalytic access to mono- and bisspiro-2-amino-4H-pyrans under ultrasound irradiation.

In 2007, Azizian and co-workers developed an efficient microwave-assisted one-pot protocol for the synthesis of novel spiro [indoline-3,2-thiazolidine]-2,4′(1*H*)-diones and bisspiro [indoline-3,2-thiazolidine]-2,4′(1*H*)-diones 303*via* a condensation reaction of isatin 6, mercaptoacetic acid 301, and aromatic amines 302 (Scheme 72).^173^ Following this work, Jarrahpour and Khalili described the first example of the synthesis of bis-spiro-β-lactams 305 from the reaction of di-imines and ketenes derived from phenoxy and phthaloylglycyl chlorides. In this study, some new mono- and bisspiro-β-lactams of benzylisatin were prepared by Staudinger's ketene–imine [2+2] cycloaddition reaction (Scheme 73).^174^ The unique structural features of these spiro-β-lactams make them promising precursors for the synthesis of modified β-amino acids, β-peptides, and monobactam analogues.

**Scheme 72:**
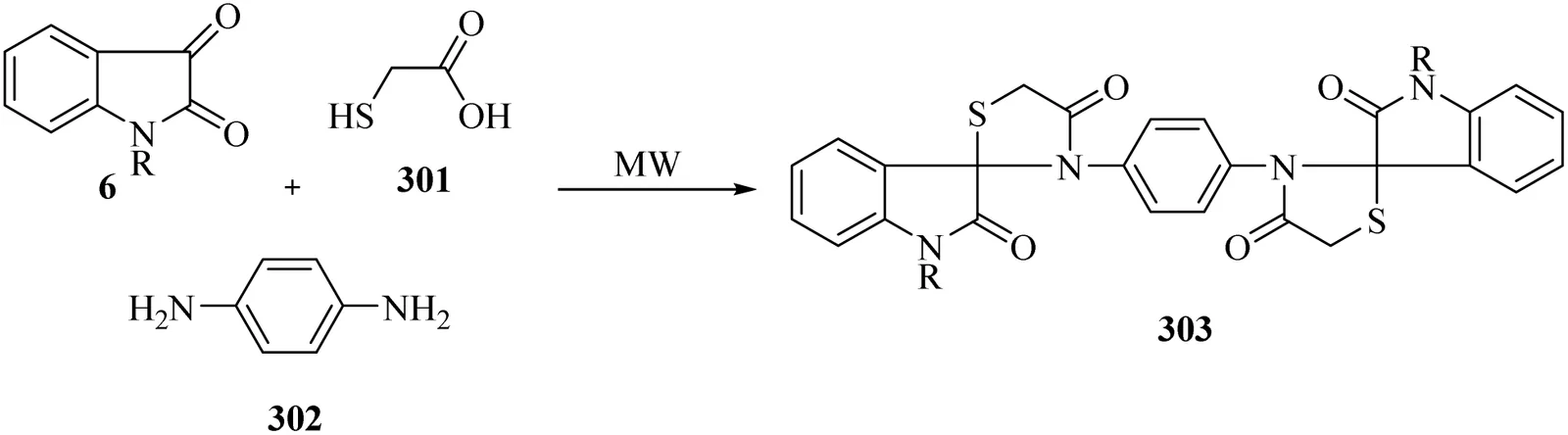
Microwave-induced synthesis of spiro[indoline-3,2′ thiazolidine]-2,4′(1H)-diones and bis [spiro[indoline-3,2′ thiazolidine]-2,4′(1H)-diones].

**Scheme 73:**
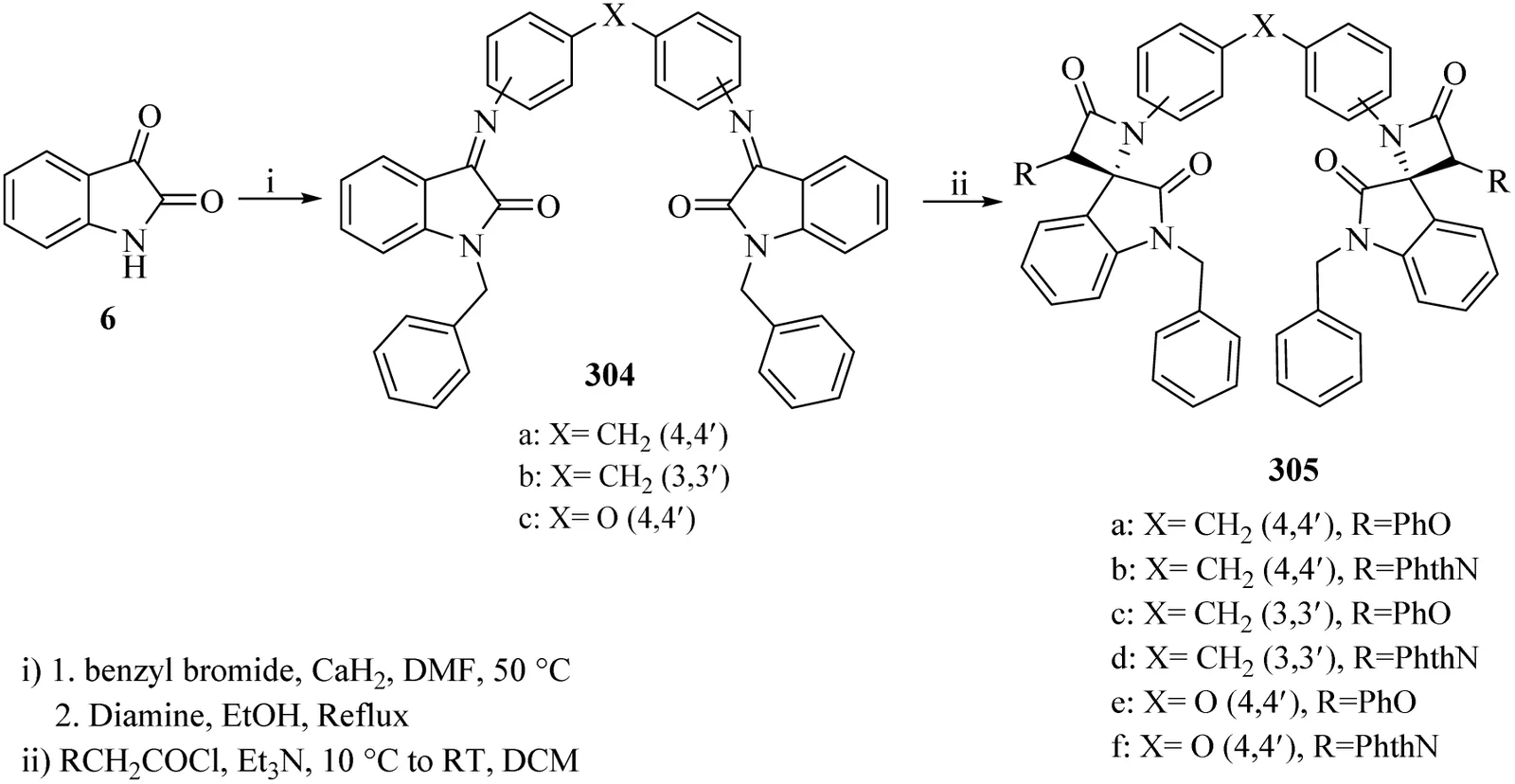
Construction of some mono- and bisspiro-β-lactams of benzylisatin.

## Conclusion

In conclusion, this review provides a comprehensive analysis of the synthetic strategies, mechanistic features, and biological properties of bisspirooxindole derivatives, highlighting major advances and remaining challenges in this rapidly developing field. The collected literature demonstrates that cycloaddition and multicomponent reactions (MCRs) are among the most widely employed and synthetically powerful approaches for constructing bisspirooxindole frameworks. Their ability to generate structurally complex architectures and multiple stereogenic centers in a convergent manner, often in a single synthetic operation, makes them particularly attractive for the efficient preparation of bisspirooxindole libraries. Among these methodologies, cycloaddition reactions involving azomethine ylied-based strategies appear particularly compatible with the principles of green chemistry, owing to their convergent nature, efficient construction of molecular complexity, and potential to reduce synthetic steps, intermediate isolation, and waste generation. In addition, the use of catalytic conditions, mild reaction parameters, and readily accessible starting materials further supports their potential for sustainable synthesis. Nevertheless, future studies should focus more on environmentally preferable solvents, reduced catalyst and reagent loadings, improved energy efficiency, minimized waste and purification requirements, and, importantly, validation at gram or multigram scales. Thus, future methodological development should place emphasis not solely on synthetic yield and stereoselectivity but also on green-chemistry metrics, process efficiency, and scalability.

From a pharmacological perspective, the reviewed studies confirm the considerable biological potential of the bisspirooxindole scaffold across several therapeutic areas. However, biological studies remain uneven, with important gaps in the structure–activity relationship (SAR) landscape. In particular, antitubercular activity remains comparatively underexplored in bisspirooxindole research. The limited number of systematically investigated derivatives and comprehensive SAR studies hinders a clear understanding of how substitution patterns, stereochemical features, and structural modifications influence antitubercular activity. This gap provides an important opportunity for future research, particularly through the systematic design and biological evaluation of structurally diverse bisspirooxindole analogues. Such studies should ideally combine SAR optimization with mechanistic investigations and standardized biological assays to identify the structural determinants responsible for antimycobacterial activity.

Overall, the literature indicates that the future development of bisspirooxindole chemistry lies at the intersection of efficient synthetic methodology and systematic medicinal chemistry. Cycloaddition and multicomponent approaches provide effective platforms for the rapid and stereoselective generation of structurally diverse bisspirooxindole scaffolds, while their optimization toward greener, more economical, and scalable processes remains an important research objective. In parallel, expanding biological investigations into underexplored areas, particularly antitubercular applications, and establishing robust SAR through systematic analogue development could enhance the medicinal potential of this scaffold. Therefore, future efforts should move beyond the synthesis and preliminary biological screening of individual compounds toward integrated strategies combining sustainable synthesis, scalable methodology, stereochemical control, systematic SAR studies, and mechanistic biological evaluation. Such an approach could open new chemical and pharmacological opportunities for bisspirooxindole-based molecular design.

## Author contributions

Fereshteh Ahmadi: investigation, writing – review & editing. Zahra Abdi: investigation, writing – original draft. Mohammad Javadi: investigation, preparation of reaction schemes.

## Conflicts of interest

There are no conflicts to declare.

## Data Availability

No new data were generated in this work. All data discussed in this review are available in the cited published literature.
